# Identification of CT radiomic features robust to acquisition and segmentation variations for improved prediction of radiotherapy-treated lung cancer patient recurrence

**DOI:** 10.1038/s41598-024-58551-4

**Published:** 2024-04-19

**Authors:** Thomas Louis, François Lucia, François Cousin, Carole Mievis, Nicolas Jansen, Bernard Duysinx, Romain Le Pennec, Dimitris Visvikis, Malik Nebbache, Martin Rehn, Mohamed Hamya, Margaux Geier, Pierre-Yves Salaun, Ulrike Schick, Mathieu Hatt, Philippe Coucke, Pierre Lovinfosse, Roland Hustinx

**Affiliations:** 1 Division of Nuclear Medicine and Oncological Imaging, University Hospital of Liège, Liège, Belgium; 2 Radiation Oncology Department, University Hospital of Brest, Brest, France; 3 LaTIM, INSERM, UMR 1101, University of Brest, Brest, France; 4 Department of Radiotherapy Oncology, University Hospital of Liège, Liège, Belgium; 5 Division of Pulmonology, University Hospital of Liège, Liège, Belgium; 6 Nuclear Medicine Department, University Hospital of Brest, Brest, France; 7 GETBO INSERM UMR 1304, University of Brest, UBO, Brest, France; 8 Medical Oncology Department, University Hospital of Brest, Brest, France

**Keywords:** Lung cancer, Statistics, Image processing

## Abstract

The primary objective of the present study was to identify a subset of radiomic features extracted from primary tumor imaged by computed tomography of early-stage non-small cell lung cancer patients, which remain unaffected by variations in segmentation quality and in computed tomography image acquisition protocol. The robustness of these features to segmentation variations was assessed by analyzing the correlation of feature values extracted from lesion volumes delineated by two annotators. The robustness to variations in acquisition protocol was evaluated by examining the correlation of features extracted from high-dose and low-dose computed tomography scans, both of which were acquired for each patient as part of the stereotactic body radiotherapy planning process. Among 106 radiomic features considered, 21 were identified as robust. An analysis including univariate and multivariate assessments was subsequently conducted to estimate the predictive performance of these robust features on the outcome of early-stage non-small cell lung cancer patients treated with stereotactic body radiation therapy. The univariate predictive analysis revealed that robust features demonstrated superior predictive potential compared to non-robust features. The multivariate analysis indicated that linear regression models built with robust features displayed greater generalization capabilities by outperforming other models in predicting the outcomes of an external validation dataset.

## Introduction

Medical imaging plays a key role in the diagnostic, staging and follow-up processes of early-stage non-small cell lung cancer (ES-NSCLC). The inclusion of image acquisitions in the planning and dose calculation steps of radiotherapy treatments enhance their effectiveness while limiting the dose exposition for the patient. Stereotactic body radiation therapy (SBRT) is the standard of care for inoperable ES-NSCLC^1,2^. Computed tomography (CT), thanks to its high geometrical accuracy, is routinely employed to perform reliable dose calculations for SBRT^3^. Fluorodeoxyglucose positron emission tomography CT ([^18^F]FDG PET/CT) is a molecular imaging technique combining metabolic and anatomical evaluation. This dual approach enhances diagnostic accuracy, refines lung cancer staging and enables better treatment optimization and therapy response monitoring^4^. In the ES-NSCLC radiotherapy planning phase, [^18^F]FDG PET/CT complements the planning CT, facilitating the accurate delineation of the target volume and the preservation of organs at risk^5^.

The extraction of quantitative, high-dimensional information from medical images with data-characterization algorithms, is commonly referred to as “Radiomics”^6^. This process has been increasingly used in recent years to analyze and predict clinical outcomes and has been efficiently applied to study NSCLC^7–10^. Radiomics is considered less robust than other omics approaches because of the different processing steps undergone by an image from its acquisition to the feature extraction^11–13^. Additionally, the lack of standardization in clinical CT acquisition protocols introduces considerable variability, which emphasizes this lack of robustness^14,15^. Post-extraction harmonization techniques such as ComBat were developed to limit the variability of the data by correcting batch effect^16,17^. Such strategies nevertheless require meeting several specific assumptions to be efficient, which limits the application on real-world datasets^18^. Despite the efforts of organizations such as the Image Biomarker Standardisation Initiative (IBSI) to develop consistent radiomics analysis workflows and identify reliable features^19,20^, it remains important to take one step further and come up with a robust approach specific to ES NCSLC treatment. This approach needs to be unaffected by the imaging protocol and segmentation and deliver unbiased information related to the patients rather than the process artifacts.

Numerous strategies have been considered to assess the robustness of radiomic procedures while minimizing patient radiation exposure from multiple CT imaging. Some studies were based on phantom models^21,22^. In vivo studies utilized several scans acquired at intentionally reduced dose levels^23^ or modeling to generate images with different acquisition parameters from a single CT scan per patient^24–28^. While these studies mainly focused on understanding the influence of operational parameters on the radiomic features and on defining optimal imaging and analysis workflows^29^, they may not represent the reality of the clinical situation, for which numerous criteria can differ between acquisitions. Additionally, many of these previous works suffer from using small dataset, which is a recurring limitation in radiomic studies.

This study capitalizes on the planning protocol of radiotherapy for ES-NSCLC, which includes both high-dose dosimetric CT and low-dose CT of PET/CT scans for each patient. This specific scenario constitutes a great opportunity to provide a realistic insight into the effects of protocol modification and uncontrolled operating variations on radiomic feature robustness. The primary objective of this work was to transcend the influence of operational conditions and to identify features which are robust to variations in imaging protocols and lung cancer segmentations. Furthermore, a multicentric preliminary assessment of the predictive properties of these robust features was conducted. The analysis was focused on the occurrence of regional or distant relapse in patients treated with SBRT. Distant metastases are more frequent than local relapse^2^ and harder to predict which justifies the interest of studying these endpoints^30,31^.

## Methods

**Figure 1:**
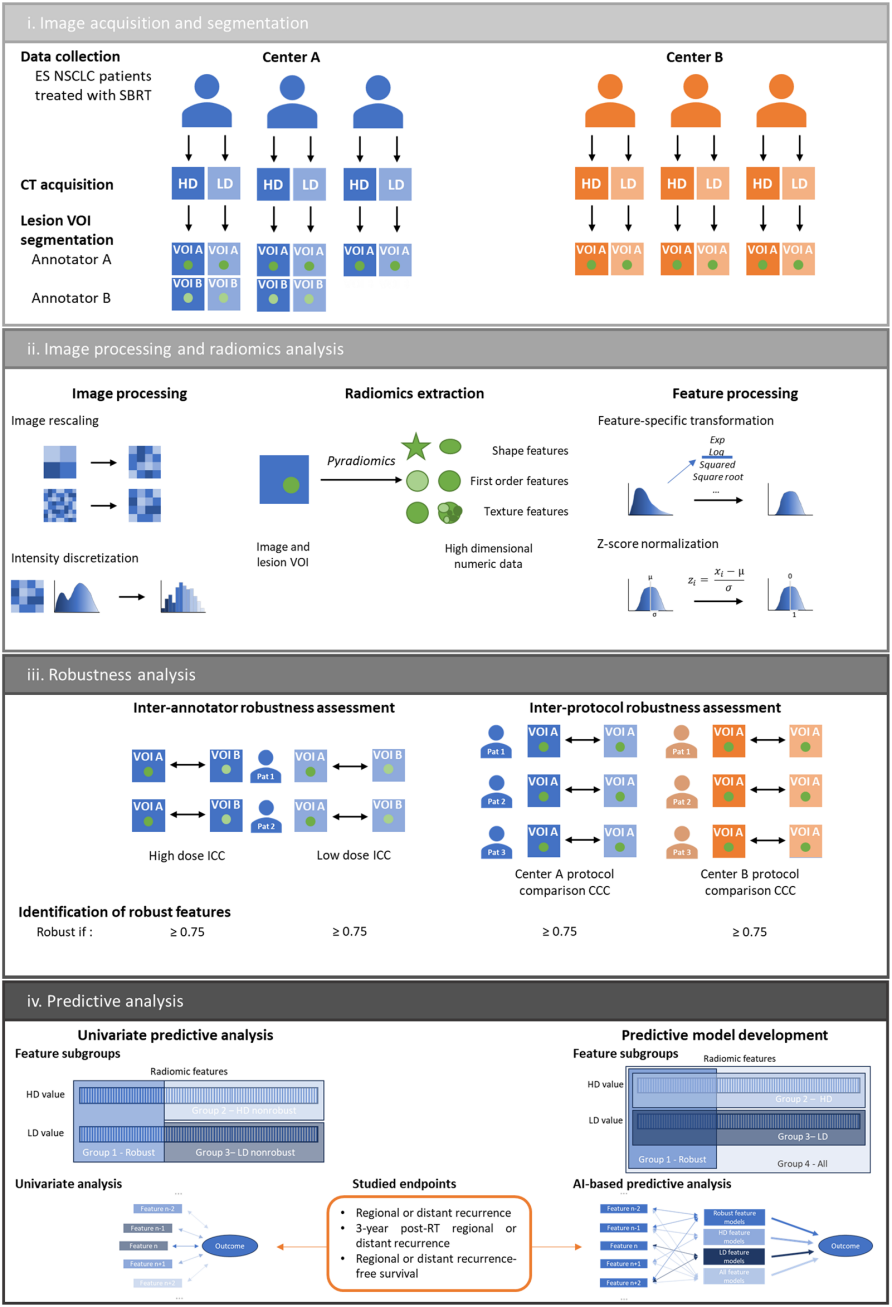
Full methodology chart. i. High dose and low dose chest computed tomography (CT) scans were conducted on patients of both centers following center’s specific imaging protocols. In each scan, the lung lesion is delineated in 3D by the annotator A. The lung lesion of 50 randomly drawn center A patients were segmented by annotator B. ii. CT scans were rescaled to 1 × 1x1mm voxel size. CT scan intensity was discretized to a fix bin count of 64. Pyradiomics was used to extract 106 features. The radiomic feature values were submitted to a feature-specific transformation selected to fit a Gaussian distribution over all center A value. A Z-score normalization was conducted on the updated feature values. iii. Inter-annotator robustness of the radiomic features was assessed by calculating the Intraclass Correlation Coefficient (ICC) (3,1) between the values extracted from the segmentations of annotator A and annotator B in high dose and low dose CT scan. Inter-protocol robustness of radiomic features was assessed by calculating Lin's Concordance Correlation Coefficient (CCC) between the values extracted from the high dose CT scan and the low dose CT scan of each patient In both centers. Robust features were identified as features with all coefficient values higher than 0.75. iv. Univariate analysis was conducted on radiomic features divided into three groups: robust features, high-dose (HD) value of non-robust features, low-dose (LD) value of non-robust features. Individual feature ability to predict regional or distant recurrence, regional or distant recurrence at 3 years and regional or distant progression free survival was studied. Distribution of performances between groups were studied. Generalized linear models (GLM) and Cox Proportional-Hazards (CoxPH) models were developed using features from four groups: Robust features, low dose features, high dose features, all features. The trained models were used to attempt at predicting the three previously cited outcomes. Performance of the models originating from the different groups were compared. Figure abbreviations: Non-Small Cell Lung Cancer (NSCLC), Radiation therapy (RT), Stereotactic Body Radiation Therapy (SBRT), Volume of interest (VOI), (AI) Artificial Intelligence.

The procedure workflow followed in this study is illustrated and described in Fig. 1.

### Patients’ selection

Patients with diagnosed ES-NSCLC T1 (< 3 cm) and T2 (3–5 cm) treated with lung SBRT between April 2010 and December 2020 were retrospectively collected in the databanks of two institutions: University Hospital (CHU) of Liège in Belgium—the center A—and University Hospital of Brest in France—the center B. This study was conducted in accordance with the Declaration of Helsinki and approved by the Institutional Review Board of CHU of Liège (protocol code 2022/285; date of approval: 08 November 2022). The requirement of written informed consent from enrolled subjects was waived by the Institutional Review Board of CHU of Liège due to the retrospective study design.

Of the 597 eligible patients, patients were not included because of [^18^F]FDG PET/CT was not available (n = 50), poor-quality of PET/CT or CT images (n = 57), or a delay between PET/CT and planning CT imaging longer than 8 weeks (n = 56). A total of 401 patients were eventually selected at center A and 33 at center B (see patient selection flowchart in Supplementary Fig. S1).

### Endpoints

Regional recurrence was defined as lymph node metastasis in the bilateral hilar, mediastinal, or supraclavicular lymph node stations. Distant recurrence was defined as failure in the same pulmonary lobe (farther than 1.5 cm from the primary tumor), in other lung lobes (ipsi or contralateral lung) or in other organs. These recurrences had to be confirmed by histology or by a multidisciplinary committee on the basis of CT and [^18^F]FDG PET/CT. Recurrence was distinguished from second primary lung tumors by considering the pathology results, the interval between the recurrence and primary tumor, and the location of the recurrence in relation to the SBRT field^32^. Regional or distant recurrence (binary), recurrence after 3 years from the first day of RT (binary) and recurrence-free survival (survival) were considered as endpoints in this study.

### Imaging modalities

High-dose (HD) CT scans of planning CT studies were captured with 2 types of scanners and 2 types of acquisition. In the CHU of Liège, expiration CT studies were acquired with a Philips Brilliance Big Bore CT (Philips Healthcare, Andover, MA, USA) and reconstructed with E kernel and a 1 mm slice thickness. In the CHU of Brest, free-breathing CT studies were performed with a Siemens Somatom (Siemens Healthcare, Malvern, PA, USA). No contrast-enhancing agent was used for the planning CT scan and reconstructed with filtered back projection B30f. kernel and a 2 mm slice thickness.

Low-dose (LD) CT scans from PET-CT studies were acquired with 3 types of scanners and 3 types of acquisition. In the CHU of Liège, studies were acquired using cross-calibrated Philips Gemini TF or BB (Philips Healthcare, Andover, MA, USA) and reconstructed with B kernel and a 3 mm slice thickness. In the CHU of Brest studies were performed with a Siemens Biograph mCT between 2016 and 2018 and with a Siemens digital Biograph Vision 600 between 2019 and 2020 (Siemens Healthcare, Malvern, PA, USA) and reconstructed using iterative reconstruction I30f kernel and a 2 mm slice thickness (see Supplementary Table S1 online for detailed acquisition information).

### Volume segmentation

All pulmonary tumor were semi-automatically segmented on HD and LD CT scans following a previously validated semi-automatic approach exploiting an open-source software, *3D Slicer* (Slicer.org), and the Growcut algorithm by an experienced radiation oncologist (F.L.) —annotator A^33^. To evaluate the influence of the lesion volume of interest (VOI) variation on radiomic feature values, and hereby the inter-annotator robustness, the tumor of 50 randomly-selected patients was segmented by another expert (F.C.)—annotator B—using a semi-automatic approach on the open-source software ITK-SNAP (itksnap.org) (see Supplementary Fig. S2 for segmentation examples).

### Image preprocessing

CT scans and related segmentations were resampled to 1 × 1 × 1 mm^3^ voxels using cubic spline interpolation and nearest neighbor interpolation respectively. CT scan voxel intensity was converted to Hounsfield units (HU).

### Radiomic feature extraction

The open-source python package *PyRadiomics* v3.0.1 was used to extract radiomic features from the CT scans after an intensity discretization with a fixed bin count of 64^29,34^. Pyradiomics enabled the extraction of a total of 106 features from a 3D segmentation in the original non-derived image (see detailed feature list given in Supplementary Table S2 online). For each patient, 106 features were extracted from the lesion volume of the high-dose CT scan and 106 features from the lesion volume of the low-dose CT scan for a total of 212 features.

### Robustness and predictive analysis

All the subsequent analyses were performed using R (v4.2.2) through RStudio (v 2023.06.1) IDE.

#### Inter-annotator robustness assessment

Dice Similarity Coefficient was used to compare segmentation between annotators^35^. The intraclass correlation coefficient (3,1) (ICC) was evaluated for each feature between annotator A and annotator B segmentation values^36^. Features with an ICC higher than 0.75 were considered as inter-annotator robust^37,38^. To express the influence of the patient’s segmentation Dice score on the variation of extracted radiomic feature value, Z-score normalization was applied on each feature and the absolute variation of Z-score between radiomic values extracted from annotator A segmentation and from annotator B segmentation was calculated.

#### Inter-protocol robustness assessment

Lin’s concordance correlation coefficient (CCC) was calculated for each feature between HD CT scan and LD CT scan values^39^. Features with an CCC higher than 0.75 were considered as inter-protocol robust following Altman’s approach.

#### Radiomic feature processing

The features were submitted to a transformation specific to the feature distribution. First, it was assessed if the feature value distribution across all center A patients followed a gaussian distribution using Shapiro’s test^40^. If not, it was assessed if the feature value distribution across all center A patients subjected to one of the following transformations followed a gaussian distribution: exponential, logarithmic, squared, cubed, square root, cubic root. If one of the transformed value distributions is significant to the Shapiro’s test, the transformation is applied to the feature values of all center A and center B patients (see the list of transformation specific to each feature in Supplementary Table S3 online). All radiomic features were normalized using Z-score normalization. ComBat harmonization method was used on all radiomic features of center A and center B patients with center A patients considered as the reference set^16^.

#### Univariate predictive analysis

Radiomic features were divided into three groups: the robust ones which featured an inter-annotator ICC and an inter-protocol CCC higher than 0.75, the HD feature value of the non-robust ones and the LD feature value of the non-robust ones. The ability of radiomic features to individually describe the regional or distant recurrence, regional or distant recurrence at 3 years and the regional or distant recurrence-free survival endpoints was evaluated. To do so, for each feature, one univariate prediction model was trained and tested on the whole center A set and one on the whole center B to predict endpoints. Generalized linear models (GLM) were used for binary endpoints and Cox progressional-hazards (Cox PH) model for the survival endpoint. The areas under the curve (AUC) of the ROC curve were calculated as performance metrics for binary endpoints and concordance for the survival endpoint. The oriented odds ratio (OR) of standardized radiomic feature for binary endpoints and hazard ratio (HR) for the survival endpoint were evaluated for each center by applying Z-score normalization over the whole set and calculating the OR/HR or the inverse of the OR/HR if it was less than one to ensure a greater-than-one value. The distribution of AUC/concordance and OR/HR between the different groups of radiomic features were then compared using Wilcoxon–Mann–Whitney tests with Bonferroni–Holm correction^41,42^.

#### Multivariate predictive analysis

The data available for each patient was distributed into four categories: Robust radiomic features, all radiomic features extracted from the HD CT scan, all radiomic features extracted from the LD CT scan, both HD and LD CT scan radiomic features. Center A dataset was first stratified in a train set and an internal validation set with a 70/30 split. A fivefold cross validation approach was used on the train set for the training and signature selection steps. For each fold, the training phase started with a feature selection using the redundancy maximum relevance (mRMR) (F-test correlation quotient (FCQ) variation for binary endpoints and Wald-test correlation quotient (WCQ) variation for the survival endpoints) on the train subset and keeping the 10 first selected features for the next step^43^. When studying binary endpoints, correction for unbalanced data was conducted on the train subset using a combination of Synthetic Minority Over-sampling Technique (SMOTE) oversampling and Tomek links undersampling^44,45^. GLM models (resp. CoxPH models) with all possible combinations of the 10 selected features as signatures were trained on the train subset to predict binary (resp. survival) endpoints. The chosen predictive model for a specific outcome and a subset of features was selected among all signatures using the one standard error rule based on the Akaike information criterion (AIC) over the fivefold cross^46,47^. The performance metrics (AUC for binary endpoints and concordance for survival endpoint) of the trained model were evaluated on the whole train set, the internal validation set and the center B dataset used as external validation set. The whole process was repeated 10 times to evaluate the stability of the signatures selected and the predictive results. The distribution of AUC of the models built to a predict binary endpoint over the 10 reps and the distribution of concordance for the survival models over 10 reps were compared between the four feature subsets using Wilcoxon-Mann–Whitney tests with Holm correction.

## Results

Among 434 selected patients who underwent SBRT for ES-NSCLC, regional and distant recurrence were found in 72 (17%) and 113 patients (26%), respectively, without significant differences between cohorts (see Supplementary Table S4 for full patient characteristics).

### Inter-annotator robustness

**Figure 2:**
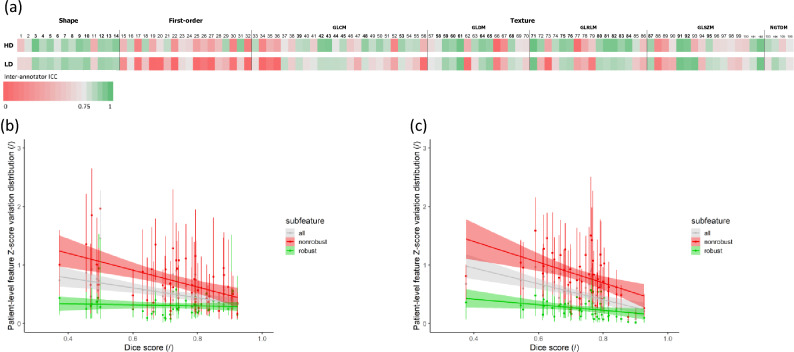
Radiomic feature inter-annotator robustness assessment. (**a**) Inter-annotator Intraclass Correlation Coefficient (ICC) of each radiomic feature in high dose and low dose CT scans. Inter-annotator robust features (highlighted in bold) were identified as features with both ICC values higher than 0.75. (**b**) Influence of the segmentation Dice score on feature Z-score variation in high dose scans. (**c**) Influence of the segmentation Dice score on feature Z-score variation in low dose scans.

The comparison of the segmentations from both annotators of the lung tumors of 50 randomly drawn patients resulted in a median Dice similarity coefficient of 0.74 (Q1–Q3: 0.66–0.83) for HD scans and 0.73 (Q1–Q3: 0.66–0.79) for LD scans. ICC (3,1) was calculated between feature values from annotator A’s and annotator B’s segmentations (given in Table 1). Out of the 106 extracted features, 59 (56%) had an ICC (3,1) greater than or equal to 0.75 for HD scans; 48 out of 106 (45%) had an ICC (3,1) greater than or equal to 0.75 for LD scans; 40 out of 106 (38%) had an ICC (3,1) greater than or equal to 0.75 for both acquisition protocols (Fig. 2.a). With a median value of 0.80 for HD CT scans (Q1–Q3: 0.53–0.89) and 0.71 for LD CT scans (Q1–Q3: 0.44–0.84), radiomic features exhibited an overall higher robustness in HD CT scans. The inter-annotator robust features were composed of shape and texture features, while no first-order intensity feature was selected. As shown in Fig. 2.b and 2.c presenting the distribution of absolute variation of radiomic feature Z-score for each patient in function of his/her segmentation Dice Score, the robust features were less influenced by the segmentation similarity than the non-robust ones.

**Table 1:**
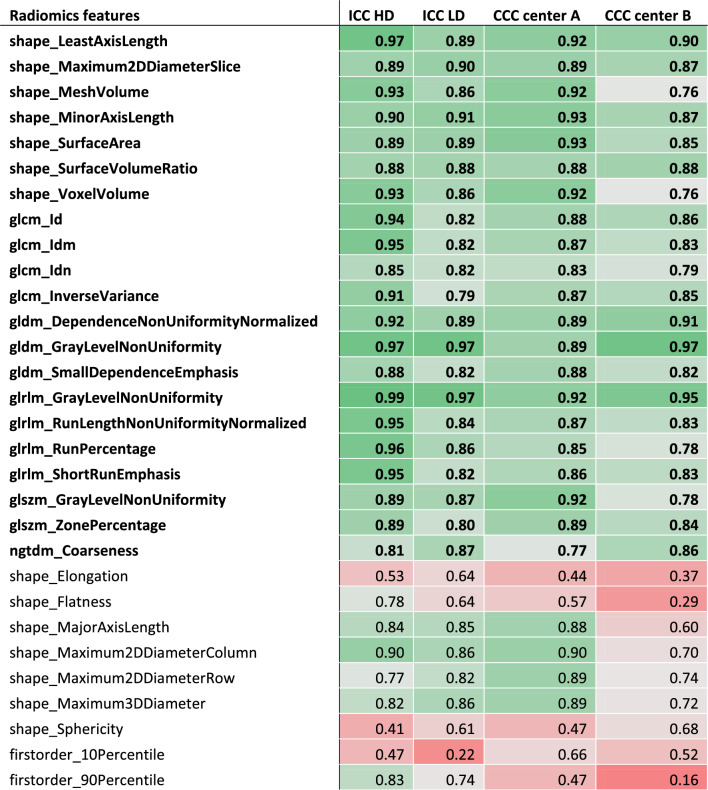

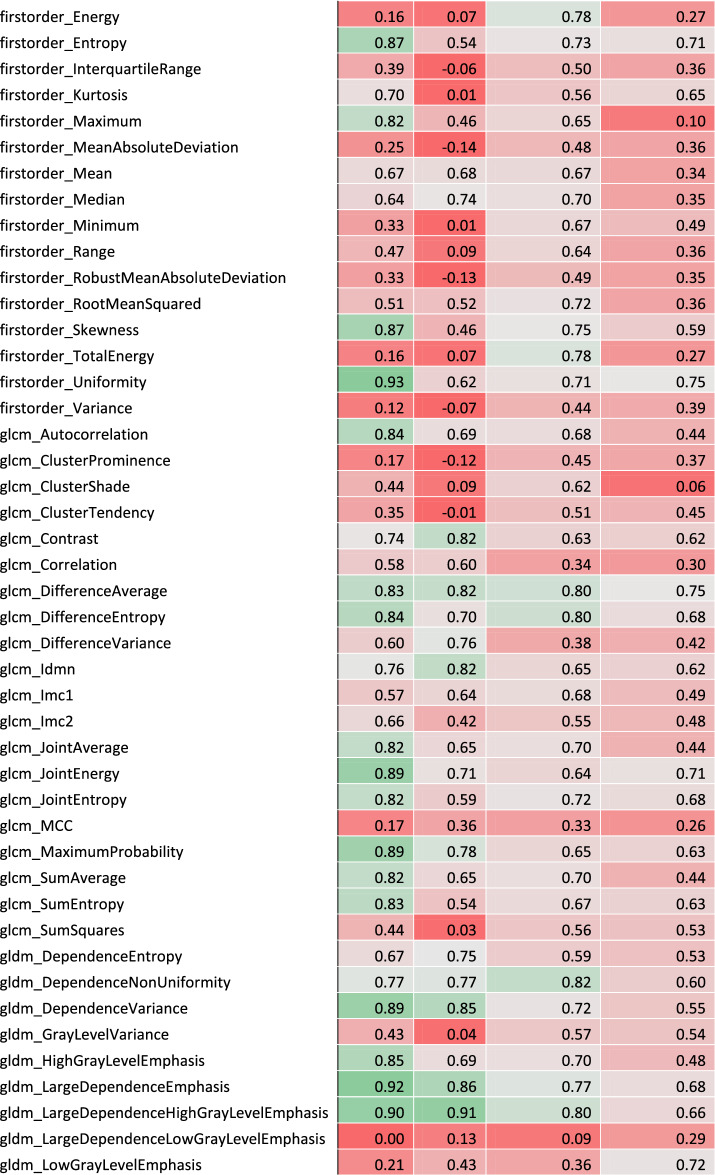

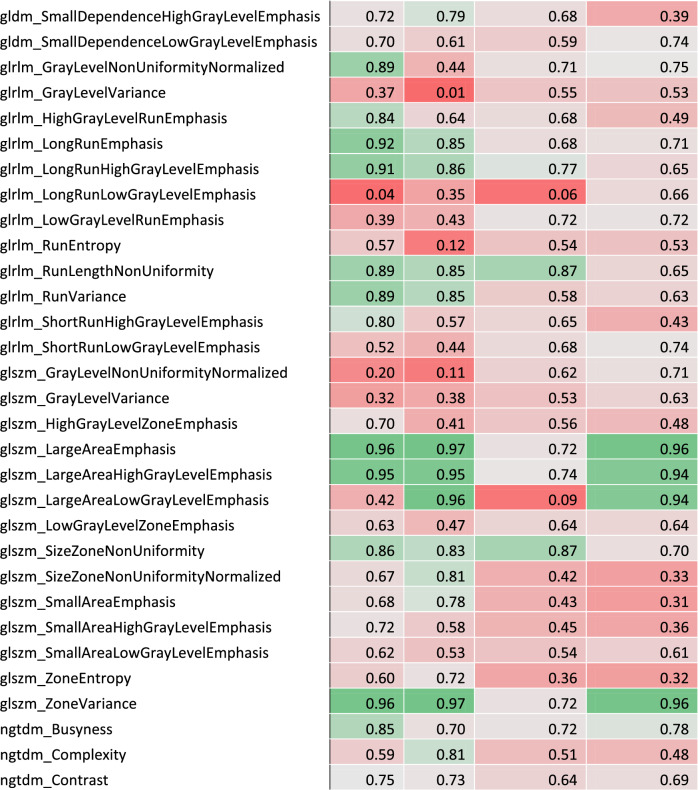
Radiomic feature coefficient table.

Complete list of intraclass correlation coefficient (ICC) and Lin's concordance correlation coefficient (CCC) for each feature. Robust features are highlighted in bold and placed at the beginning of the table.

### Inter-protocol robustness

**Figure 3:**
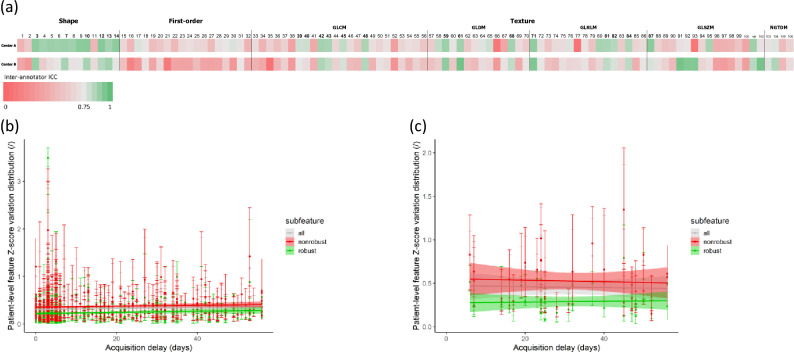
Radiomic feature inter-protocol robustness assessment. (**a**) Inter-protocol Lin's Concordance Correlation Coefficient (CCC) of each radiomic feature in patients from center A and from center B. Inter-protocol robust features (highlighted in bold) were identified as features with both CCC values higher than 0.75. (**b**) Influence of the delay between acquisitions on feature Z-score variation in center A. (**c**) Influence of the delay between acquisitions on feature Z-score variation in center B.

Lin’s CCC was calculated for each radiomic feature between HD and LD scan values in center A and center B (shown in Fig. 3a and given in Table 1). Out of the 106 extracted features, 35 (33%) had a CCC greater than or equal to 0.75 in center A; 21 out of 106 (20%) had a CCC greater than or equal to 0.75 in center B; 21 out of 106 (20%) had a CCC greater than or equal to 0.75 in both centers. With a median value of 0.68 (Q1–Q3: 0.56–0.81) in center A and 0.63 (Q1–Q3: 0.44–0.75) in center B, radiomic features exhibited an overall higher robustness in center A. The inter-protocol robust features were composed of shape and texture features, while no first-order intensity feature was selected. All inter-protocol robust features were also inter-annotator robust. As shown in Fig. 3b,c, the delay between the two scans, which was limited to 56 days, has little influence on the absolute variation of radiomic feature Z-score between the scans independently of the feature robustness status.

### Univariate predictive analysis

**Figure 4:**
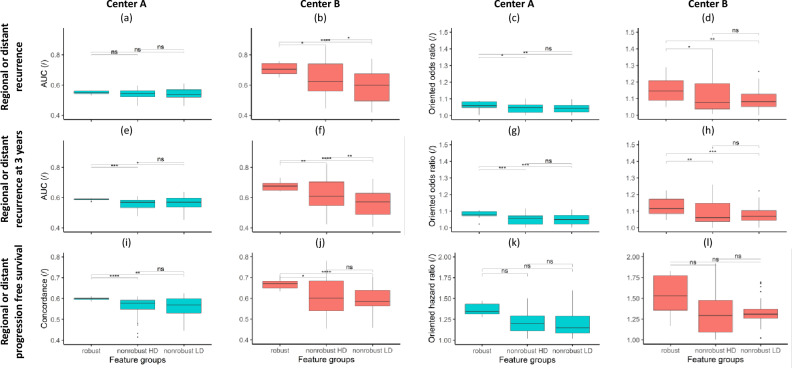
Univariate predictive analysis. Distributions of univariate AUC from the different feature groups for the prediction of regional or distant recurrence in center A (**a**) and center B (**b**), for the prediction of regional or distant recurrence at three years in center A (**e**) and center B (**f**). Distributions of odds ratio from the different feature groups for the prediction of regional or distant recurrence in center A (**c**) and center B (**d**), for the prediction of regional or distant recurrence at three years in center A (**g**) and center B (**h**). Distributions of univariate concordance from the different feature groups for the prediction of regional or distant progression free survival in center A (**i**) and center B (**j**). Distributions of hazard ratio from the different feature groups for the prediction of regional or distant progression free survival in center A (**k**) and center B (**l**).

The predictive performances of the features distributed into the three groups labeled robust, non-robust HD and non-robust LD were evaluated in center A and in center B. The AUC and the oriented OR were calculated for the regional or distant recurrence start endpoint. The comparison of distributions of AUC and oriented OR between the three groups in center A and center B are shown in Fig. 4a–d. Slightly higher AUC and oriented OR were observed in robust features compared to HD and LD non-robust ones in centers A and B.

Univariate results for the regional or distant recurrence at 3 years post-RT endpoint are shown in Fig. 4e–h. Significantly higher AUC and oriented OR were observed in robust features compared to HD and LD non-robust ones in centers A and B.

The univariate analysis of the feature ability to predict regional or distant recurrence free survival gave analogous results (shown in Fig. 4i–l). Significantly higher concordance and higher oriented HR were observed in robust features compared to HD and LD non-robust ones in centers A and B.

See Supplementary Data S1 for a comprehensive statistical description of the univariate analysis results. See Supplementary Table S5a–c for detailed univariate analysis results on regional or distant recurrence prediction, 3-year post-RT regional or distant recurrence prediction and regional or distant recurrence free survival respectively.

The 21 features eventually selected were least axis length, maximum 2D diameter slice, mesh volume, minor axis length, surface area, surface volume ratio, voxel volume, Gray Level Co-occurrence Matrix (GLCM) inverse difference, GLCM inverse difference moment, GLCM inverse difference normalized, GLCM inverse variance, Gray Level Dependence Matrix (GLDM) dependence non-uniformity normalized, GLDM grey-level non-uniformity, GLDM small dependence emphasis, Gray Level Run Length Matrix (GLRLM) grey-level non uniformity, GLRLM run length non-uniformity normalized, GLRLM run percentage, GLRLM short run emphasis, Gray Level Size Zone Matrix (GLSZM) gray-level non-uniformity, GLSZM zone percentage and Neighboring Gray Tone Difference Matrix (NGTDM) coarseness.

### Multivariate predictive analysis

**Figure 5:**
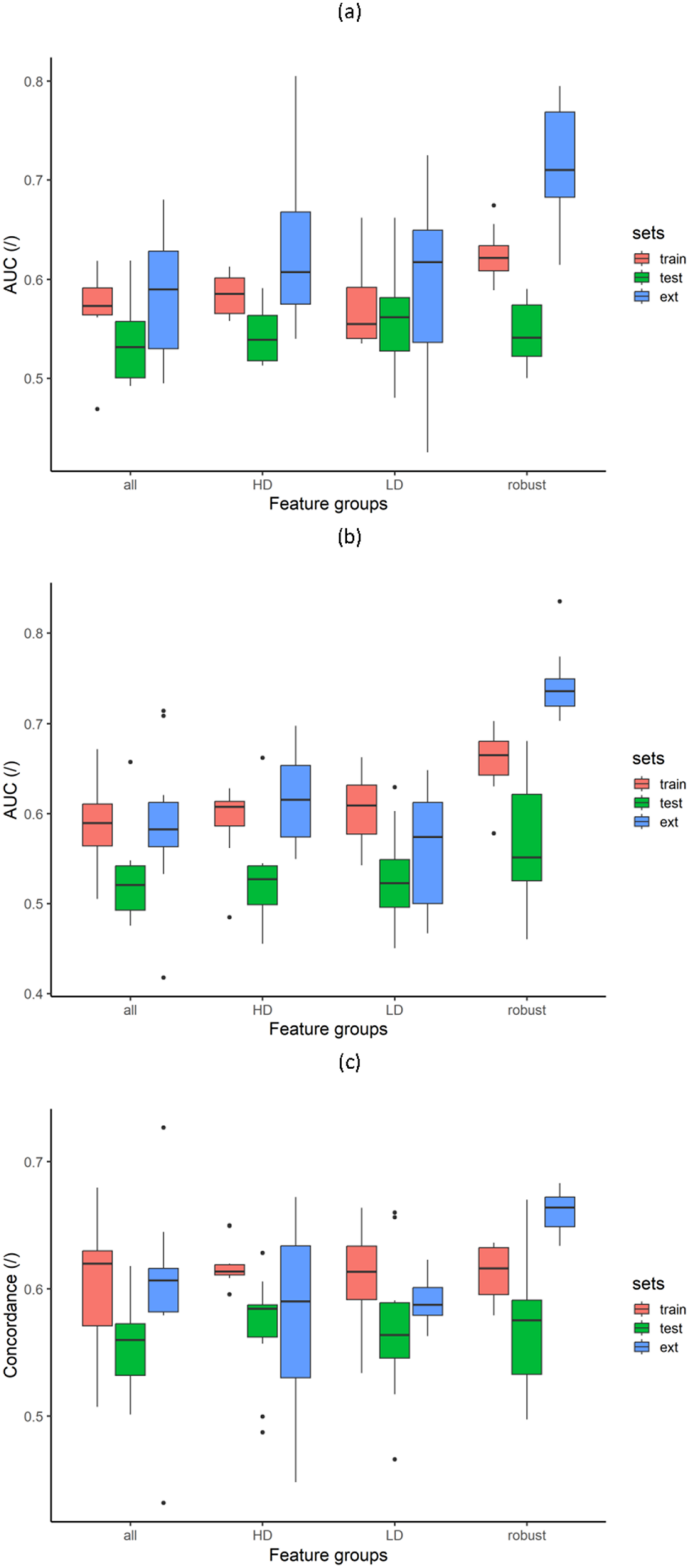
Multivariate predictive analysis. Prediction of region or distant recurrence (**a**), 3-year post-RT regional or distant recurrence (**b**) and regional or distant progression free survival (**c**) using generalized linear models (GLM), GLM and Cox Proportional-Hazards (CoxPH) models respectively.

For each subset of features and each endpoint, at each iteration of the five folds and of the ten repetitions, ${2}^{10}-1$ signatures were generated with the 10 features selected by mRMR. The GLM and Cox PH models based on these signatures were trained and tested in the fivefold cross-validation and one signature was selected with the one standard error rule based on the AIC. The distributions of AUC and concordance of the selected models used on the train, internal validation and external validation sets to predict regional or distant recurrence, 3-year post-RT regional or distant recurrence and regional or distant recurrence free survival are shown in Fig. 5a–c respectively. A comprehensive description of the multivariate analysis results is given in Supplemental Data S2.

Wilcoxon–Mann–Whitney tests with Holm correction were additionally performed to compare results on the train, internal validation and external validation sets separately (see full comparison results in Supplementary Table S6a–c online). The predictive performance of the models built with robust features on the train and internal validation set demonstrated similar-to-greater tendencies compared to other features groups. These robust feature-only models significantly outperformed the all-feature, HD-feature and LD-feature models in predicting the three considered endpoints in the external validation set.

See Supplementary Table S7a–c online for the individual performance of the model selected at each of the 10 repetitions for each feature subset to predict regional or distant recurrence prediction, 3-year post-RT regional or distant recurrence prediction and regional or distant recurrence free survival respectively.

## Discussion

This study identified a subset of features invariant to imaging protocol variation and lesion segmentation quality. These features demonstrated a greater predictive potential for regional or distant recurrence in ES-NSCLC patients treated with SBRT through univariate and multivariate analyses.

Out of the 106 CT radiomic features studied, 40 (38%) had HD ICC(3,1) and LD ICC(3,1) values greater than or equal to 0.75 and were labeled as inter-annotator robust. The globally lower ICC in LD scans can be explained by a greater impact of segmentation variations on radiomic features extracted from lower resolution images. The features identified as robust does not seem to be influenced by the segmentation Dice score both in HD and LD scans even in poor cases (Dice score < 0.5). It means that these features are highly invariant to the segmentation quality and that the texture features should be less influenced by intensity values at the border of the segmentation. Shape features identified as robust between annotators were primarily determined by the size of the segmentations, whereas non-robust features were affected by their shapes. The diminished impact of the Dice score on robust shape features suggests that while the segmentations from the two annotators were similar in size, they must have exhibited slight variations in shape.

Features value variation analysis between HD and LD scans in center A and center B led to the selection of 21 (20%) inter-protocol robust features. The variation of value for both robust and non-robust features between HD and LD scans does not seem to be influenced by the delay between acquisition in the 8-week limit range. The 21 features selected as robust regarding the imaging protocol are also robust regarding the annotator.

Amongst the 21 features eventually selected, 7 are shape factors and the others are texture features, including 4 GLCM features, 3 GLDM features, 4 GLRLM, 2 GLSZM features and 1 NGTDM feature. The high proportion of shape features identified as robust (7 out of 14 shape features initially extracted) could be expected since the shape of the lesion should not be greatly influenced by the imaging protocol. No first-order intensity feature was selected as robust. This contradicted other studies which claimed that intensity features, along with shape features, are more robust than texture ones^48,49^. The high variability of first order statistics may nevertheless be explained by the significant difference in intensity between the tumor and its neighboring area leading to major intensity fluctuations at the border of the lesion. GLDM gray-level non-uniformity, GLSZM gray-level non-uniformity were already identified as reproducible in response to dose and kernel variations^24^. GLRLM gray level non-uniformity was reported as promising feature for NSCLC prognosis^50^.

The decision to consider the regional or distant recurrence after a fixed follow-up time of 3 years as an endpoint came from the high variability of follow-up time and time of occurrence between patients. A delay of 3 years after radiotherapy allowed to include most patients while featuring a sufficient level of occurrence.

Robust features exhibited higher AUC/concordance and oriented OR/HR than non-robust ones to predict regional or distant recurrence. This addresses the concern that, by only considering features with common information between protocols, we neglect the subtle but relevant information in the images^51,52^. Non-robust HD features exhibited better results than non-robust LD features indicating that higher dose images globally contain more information. While the median individual AUCs and concordance for the center A dataset were between 0.55 and 0.6, indicating an overall limited univariate predictive power, this study preferably focused on the generalization of the selected features and the greater predictive potential which they demonstrated.

The multivariate predictive analysis resulted in similar tendencies for all endpoints. The similar-to-greater performances of the robust features-only models were consistent with what was observed in the univariate analysis. Even if the other feature groups also included the robust features in the multivariate analysis, the presence of the other features could dilute the information and the feature selection step before the model development was not sufficient to properly identify the relevant ones. The higher AUC/concordance of the robust feature-only model on the external validation set translated the better generalization ability of the robust radiomic features : while results for the train and test subsets, which both consisted of center A patients, were similar for signatures including robust and non-robust features, the greater performance of robust-feature only signatures on the external validation dataset composed of center B patients proved that non-robust features were not able to maintain their predictive potential when applied to datasets with other image acquisition conditions.

The present study differs from the literature by describing a multicentric approach which includes high-dose dosimetric CT and low-dose CT of PET/CT scans for each patient by benefiting from the planning protocol of radiotherapy for ES-NSCLC. It goes one step further than studies relying on phantom models^21,22^ or artificially-perturbated scans^24–28^, as it uses authentic unaltered patient scans with distinct acquisition protocols to identify both inter-annotator and inter-protocol robust radiomic features with a comprehensive and complete approach. The added value of the identified robust features is then brought forward by univariate and multivariate analyses.

It nevertheless suffers from some limitations. The dataset of CHU Brest is small and numerous patients were additionally excluded due to too much delay between high-dose and low-dose acquisition. The limited number of patients could have influenced the selection of features identified as robust in the inter-protocol robustness analysis. The CCC values were however globally lower for center B than center A resulting in a stricter feature selection due to center B. The higher predictive power of robust features in center B could also be interpreted with caution but similar relative tendencies between groups are observed for center A. While the feature selection could be generalized to other studies with ES-NSCLC patients, the results and discussions related to the univariate and multivariate predictive analysis are specific to patient response to ESBR treatment. Additional studies must be conducted to evaluate the generalization of these results to other clinical strategies.

To conclude, in this proof-of-concept study, we have identified a subgroup of features that are not affected by the segmentation quality and the imaging protocols. This group of features demonstrated greater predictive performance on outcomes of ES-NSCLC patients treated with SBRT. Limiting the model development to 21 features may overlook valuable information in more standardized acquisition protocols and more elaborate machine learning models. These features are nevertheless a solid basis for the development of models in multicentric studies.

### Supplementary Information

Supplementary Information.

## Data Availability

The datasets generated during and/or analyzed during the current study are available from the corresponding author on reasonable request.
