# Supplementary material for: Identification of CT radiomic features robust to acquisition and segmentation variations for improved prediction of radiotherapy-treated lung cancer patient recurrence
Source: Sci Rep. 2024 Apr 19;14:9028. doi: 10.1038/s41598-024-58551-4 (PMC11031577; doi:10.1038/s41598-024-58551-4)
Supplement: Supplementary file 1 — Supplementary Information. [file 41598_2024_58551_MOESM1_ESM.docx]

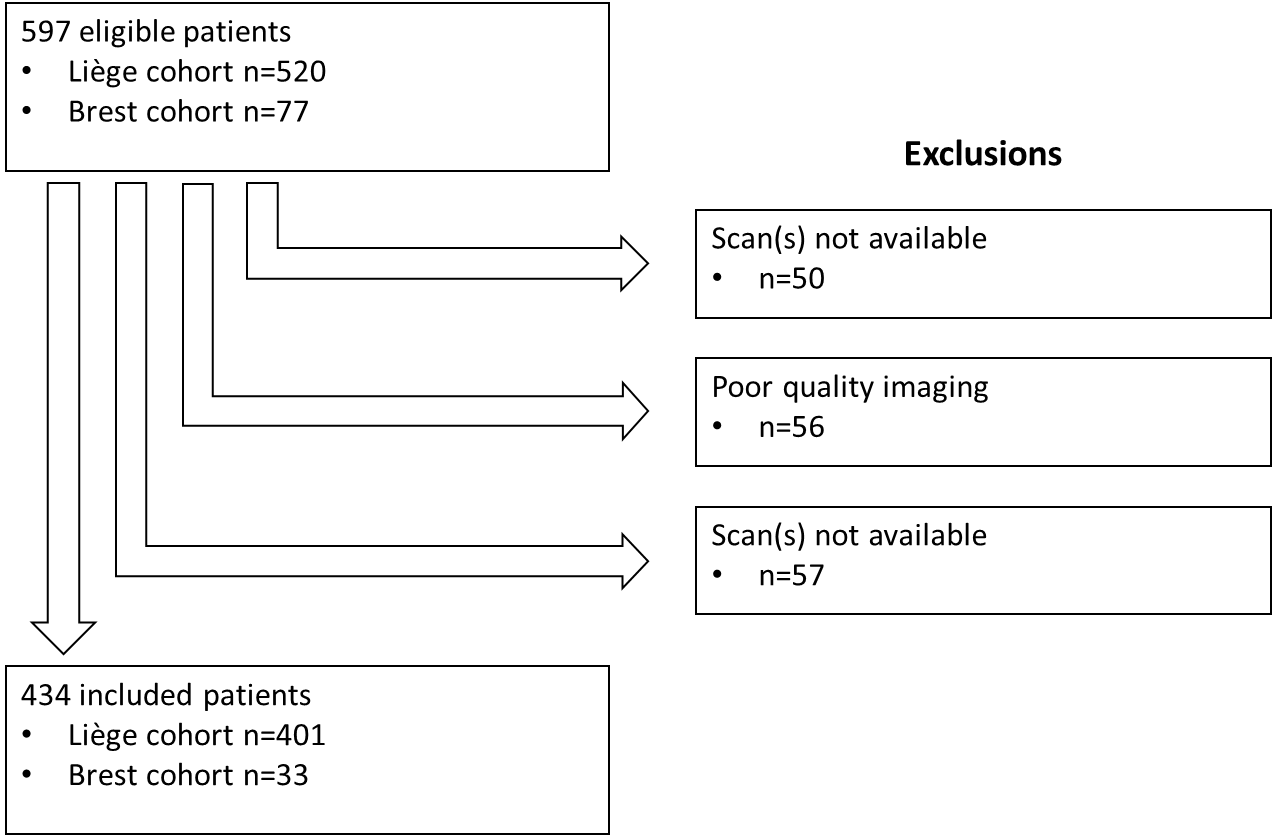


Supplementary Figure S1 –Patient selection flowchart.

| (a)  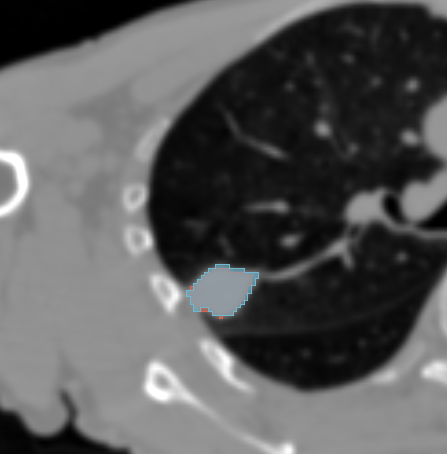 | (b)  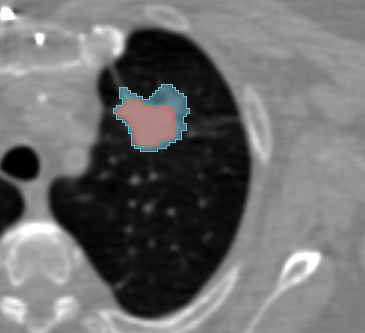 |
| --- | --- |
| (c)  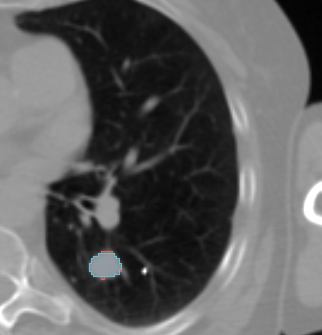 | (d)  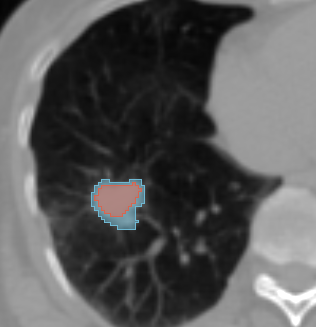 |

Supplementary Figure S2 – Examples of CT lung tumor segmentation in high dose scans ((a) and (b)) and low dose scans ((c) and(d)) with high ((a) and (c)) and low Dice scores ((b) and (d)). Annotator A segmentations are illustrated in blue. Annotator B segmentations are illustrated in red. Segmentations in (a), (b), (c) and (d) gave a Dice score of 0.92, 0.50, 0.93 and 0.55 respectively.

**Supplementary Data S1 – Comprehensive description of univariate analysis statistical results**

**Regional or distant recurrence prediction**

With a median AUC of 0.55 (Q1-Q3: 0.54-0.56) in center A and 0.70 (Q1-Q3: 0.68-0.74) in center B, robust features exhibited a slightly higher predictive power than non-robust HD features (0.55 (Q1-Q3: 0.52-0.56) in center A and 0.63 (Q1-Q3: 0.56-0.74) in center B) and non-robust LD features (0.54 (Q1-Q3: 0.52-0.57) in center A and 0.60 (Q1-Q3: 0.50-0.68) in center B). This tendency was also observed with the oriented odds ratio in center A (1.06 (Q1-Q3: 1.05-1.08) for robust features against 1.05 (Q1-Q3: 1.02-1.06) for non-robust HD features and 1.04 (Q1-Q3: 1.02-1.06) for non-robust LD features) and center B (1.14 (Q1-Q3: 1.09-1.21) for robust features against 1.08 (Q1-Q3: 1.04-1.20) for non-robust HD features and 1.08 (Q1-Q3: 1.05-1.13) for non-robust LD features). In center B, these differences in AUC and oriented OD were highlighted by significant MW with Holm correction tests.

**Regional or distant recurrence at 3 years post-RT prediction**

Significant differences were observed between robust and non-robust HD and LD features in AUC (0.58 (Q1-Q3: 0.54-0.61) for robust against 0.57 (Q1-Q3: 0.53-0.58) for non-robust HD and 0.57 (Q1-Q3: 0.54-0.60) for non-robust LD in center A; 0.68 (Q1-Q3: 0.65-0.70) for robust against 0.60 (Q1-Q3: 0.54-0.70) for non-robust HD and 0.57 (Q1-Q3: 0.49-0.63) for non-robust LD in center B) and in oriented odds ratio (1.08 (Q1-Q3: 1.07 -1.1) for robust against 1.06 (Q1-Q3: 1.02-1.07) for non-robust HD and 1.05 (Q1-Q3: 1.02-1.07) for non-robust LD in center A; 1.12 (Q1-Q3: 1.08-1.17) for robust against 1.06 (Q1-Q3: 1.04-1.15) for non-robust HD and 1.07 (Q1-Q3: 1.04-1.10) for non-robust LD in center B).

**Regional or distant recurrence-free survival prediction**

Significantly higher concordance was observed for robust features (0.60 (Q1-Q3: 0.60-0.60) in center A and 0.67 (Q1-Q3: 0.65-0.68) in center B) compared to non-robust HD features (0.58 (Q1-Q3: 0.55-0.59) in center A and 0.61 (Q1-Q3: 0.54-0.69) in center B) and non-robust LD features (0.57 (Q1-Q3: 0.53-0.60) in center A and 0.59 (Q1-Q3: 0.56-0.64) in center B). Higher oriented hazard ratio was observed for robust features (1.35 (Q1-Q3: 1.31-1.44) in center A and 1.83 (Q1-Q3: 1.38-2.24) in center B) compared to non-robust HD features (1.23 (Q1-Q3: 1.11-1.31) in center A and 1.43 (Q1-Q3: 1.19-2.27) in center B) and non-robust LD features (1.15 (Q1-Q3: 1.08-1.26) in center A and 1.37 (Q1-Q3: 1.28-1.68) in center B).

**Supplementary Data S2 – Comprehensive description of multivariate analysis statistical results**

**Regional or distant recurrence prediction**

Selected signatures for regional or distant recurrence prediction resulted in a median AUC of 0.62 (Q1-Q3: 0.61-0.63) for robust features, of 0.57 (Q1-Q3: 0.56-0.59) for all features, of 0.59 (Q1-Q3: 0.57-0.60) for HD features, of 0.55 (Q1-Q3: 0.54-0.59) for LD features in the train set. In the internal and external validation sets, the median AUC were of 0.54 (Q1-Q3: 0.52-0.57) and 0.71 (Q1-Q3: 0.68-0.77) for robust features, of 0.53 (Q1-Q3: 0.50-0.56) and 0.59 (Q1-Q3: 0.53-0.63) for all features, of 0.54 (Q1-Q3: 0.52-0.56) and 0.61 (Q1-Q3: 0.58-0.67) for HD features, of 0.56 (Q1-Q3: 0.53-0.58) and 0.62 (Q1-Q3: 0.54-0.65) for LD features.

**Regional or distant recurrence at 3 years post-RT prediction**

Selected signatures for 3-year post-RT regional or distant recurrence prediction resulted in a median AUC of 0.67 (Q1-Q3: 0.64-0.68) for robust features, of 0.59 (Q1-Q3: 0.56-0.61) for all features, of 0.61 (Q1-Q3: 0.59-0.61) for HD features, of 0.61 (Q1-Q3: 0.58-0.63) for LD features in the train set. In the internal and external validation sets, the median AUC were of 0.55 (Q1-Q3: 0.53-0.62) and 0.74 (Q1-Q3: 0.72-0.75) for robust features, of 0.52 (Q1-Q3: 0.49-0.54) and 0.58 (Q1-Q3: 0.56-0.61) for all features, of 0.53 (Q1-Q3: 0.50-0.54) and 0.61 (Q1-Q3: 0.57-0.65) for HD features, of 0.52 (Q1-Q3: 0.50-0.55) and 0.57 (Q1-Q3: 0.50-0.61) for LD features.

**Regional or distant recurrence-free survival prediction**

The multivariate analysis on the prediction of regional or distant recurrence free survival gave a median concordance of 0.62 (Q1-Q3: 0.60-0.63) for robust features, of 0.62 (Q1-Q3: 0.57-0.63) for all features, of 0.61 (Q1-Q3: 0.61-0.62) for HD features, of 0.61 (Q1-Q3: 0.59-0.63) for LD features in the train set. In the internal and external validation sets, the median concordance were of 0.58 (Q1-Q3: 0.53-0.59) and 0.66 (Q1-Q3: 0.65-0.67) for robust features, of 0.56 (Q1-Q3: 0.53-0.57) and of 0.61 (Q1-Q3: 0.58-0.62) for all features, of 0.58 (Q1-Q3: 0.56-0.59) and 0.59 (Q1-Q3: 0.53-0.63) for HD features, of 0.56 (Q1-Q3: 0.55-0.59) and 0.59 (Q1-Q3: 0.58-0.60) for LD features.

|  | **Center A**  **High dose CT** | **Center A**  **Low dose CT** | **Center B**  **High dose CT** | **Center B**  **Low dose CT** |
| --- | --- | --- | --- | --- |
| **Equipment** | Philips Brilliance Big Bore | Philips Gemini TF or Philips Gemini BB | Siemens Somatom | Siemens Biograph mCT or  Siemens digital Biograph Vision 600 |
| **Dose** | High | Low | High | Low |
| **Breathing** | Expiration | Free-breathing | Free-breathing | Free-breathing |
| **Slice thickness (mm)** | 1 | 3 | 2 | 2 |
| **Slice spacing (mm)** | 1.36 | 1.36 | 0.97 | 0.97 |
| **Recon. Kernel** | E (hard) | B (soft) | B30f (soft) | I30f (soft) |
| **Tube voltage (kV)**  Median [range] | 120 | 120 | 120 | 100-120 (mCT)  100-140 (V600) |
| **Tube current (mA)**  Median [range] | 490 | 110 [69-110] | 40 [40-80] | 21 [19-26.5] (mCT)  34.5 [21-88] (V600) |
| **CTDIvol (mGy)**  Median [range] | 11.9 | 4.2 | - | 0.66 [0.47-0.83] (mCT)  0.84 [0.5-3.24] (V600) |

Supplementary Table S1 – Imaging protocol operating parameters

|  | **Feature Name** |
| --- | --- |
| 1 | shape Elongation |
| 2 | shape Flatness |
| 3 | shape LeastAxisLength |
| 4 | shape MajorAxisLength |
| 5 | shape Maximum2DDiameterColumn |
| 6 | shape Maximum2DDiameterRow |
| 7 | shape Maximum2DDiameterSlice |
| 8 | shape Maximum3DDiameter |
| 9 | shape MeshVolume |
| 10 | shape MinorAxisLength |
| 11 | shape Sphericity |
| 12 | shape SurfaceArea |
| 13 | shape SurfaceVolumeRatio |
| 14 | shape VoxelVolume |
| 15 | firstorder 10Percentile |
| 16 | firstorder 90Percentile |
| 17 | firstorder Energy |
| 18 | firstorder Entropy |
| 19 | firstorder InterquartileRange |
| 20 | firstorder Kurtosis |
| 21 | firstorder Maximum |
| 22 | firstorder MeanAbsoluteDeviation |
| 23 | firstorder Mean |
| 24 | firstorder Median |
| 25 | firstorder Minimum |
| 26 | firstorder Range |
| 27 | firstorder RobustMeanAbsoluteDeviation |
| 28 | firstorder RootMeanSquared |
| 29 | firstorder Skewness |
| 30 | firstorder TotalEnergy |
| 31 | firstorder Uniformity |
| 32 | firstorder Variance |
| 33 | glcm Autocorrelation |
| 34 | glcm ClusterProminence |
| 35 | glcm ClusterShade |
| 36 | glcm ClusterTendency |
| 37 | glcm Contrast |
| 38 | glcm Correlation |
| 39 | glcm DifferenceAverage |
| 40 | glcm DifferenceEntropy |
| 41 | glcm DifferenceVariance |
| 42 | glcm Id |
| 43 | glcm Idm |
| 44 | glcm Idmn |
| 45 | glcm Idn |
| 46 | glcm Imc1 |
| 47 | glcm Imc2 |
| 48 | glcm InverseVariance |
| 49 | glcm JointAverage |
| 50 | glcm JointEnergy |
| 51 | glcm JointEntropy |
| 52 | glcm MCC |
| 53 | glcm MaximumProbability |
| 54 | glcm SumAverage |
| 55 | glcm SumEntropy |
| 56 | glcm SumSquares |
| 57 | gldm DependenceEntropy |
| 58 | gldm DependenceNonUniformity |
| 59 | gldm DependenceNonUniformityNormalized |
| 60 | gldm DependenceVariance |
| 61 | gldm GrayLevelNonUniformity |
| 62 | gldm GrayLevelVariance |
| 63 | gldm HighGrayLevelEmphasis |
| 64 | gldm LargeDependenceEmphasis |
| 65 | gldm LargeDependenceHighGrayLevelEmphasis |
| 66 | gldm LargeDependenceLowGrayLevelEmphasis |
| 67 | gldm LowGrayLevelEmphasis |
| 68 | gldm SmallDependenceEmphasis |
| 69 | gldm SmallDependenceHighGrayLevelEmphasis |
| 70 | gldm SmallDependenceLowGrayLevelEmphasis |
| 71 | glrlm GrayLevelNonUniformity |
| 72 | glrlm GrayLevelNonUniformityNormalized |
| 73 | glrlm GrayLevelVariance |
| 74 | glrlm HighGrayLevelRunEmphasis |
| 75 | glrlm LongRunEmphasis |
| 76 | glrlm LongRunHighGrayLevelEmphasis |
| 77 | glrlm LongRunLowGrayLevelEmphasis |
| 78 | glrlm LowGrayLevelRunEmphasis |
| 79 | glrlm RunEntropy |
| 80 | glrlm RunLengthNonUniformity |
| 81 | glrlm RunLengthNonUniformityNormalized |
| 82 | glrlm RunPercentage |
| 83 | glrlm RunVariance |
| 84 | glrlm ShortRunEmphasis |
| 85 | glrlm ShortRunHighGrayLevelEmphasis |
| 86 | glrlm ShortRunLowGrayLevelEmphasis |
| 87 | glszm GrayLevelNonUniformity |
| 88 | glszm GrayLevelNonUniformityNormalized |
| 89 | glszm GrayLevelVariance |
| 90 | glszm HighGrayLevelZoneEmphasis |
| 91 | glszm LargeAreaEmphasis |
| 92 | glszm LargeAreaHighGrayLevelEmphasis |
| 93 | glszm LargeAreaLowGrayLevelEmphasis |
| 94 | glszm LowGrayLevelZoneEmphasis |
| 95 | glszm SizeZoneNonUniformity |
| 96 | glszm SizeZoneNonUniformityNormalized |
| 97 | glszm SmallAreaEmphasis |
| 98 | glszm SmallAreaHighGrayLevelEmphasis |
| 99 | glszm SmallAreaLowGrayLevelEmphasis |
| 100 | glszm ZoneEntropy |
| 101 | glszm ZonePercentage |
| 102 | glszm ZoneVariance |
| 103 | ngtdm Busyness |
| 104 | ngtdm Coarseness |
| 105 | ngtdm Complexity |
| 106 | ngtdm Contrast |

Supplementary Table S2 – List of radiomic features extracted by PyRadiomics and their reference numbers.

| **feature** | **-** | **Exp** | **Log** | **Squared** | **Cubed** | **Square root** | | **Cubic root** | **selected distribution** |
| --- | --- | --- | --- | --- | --- | --- | --- | --- | --- |
| shape Elongation | 0.970 | 0.985 | 0.951 | 0.987 | 0.984 | 0.948 | 0.937 | | - |
| shape Flatness | 0.986 | 0.995 | 0.972 | 0.993 | 0.970 | 0.965 | 0.954 | | Exp |
| shape LeastAxisLength | 0.911 | 0.022 | 0.995 | 0.743 | 0.557 | 0.967 | 0.980 | | Log |
| shape MajorAxisLength | 0.913 | 0.022 | 0.994 | 0.754 | 0.575 | 0.967 | 0.979 | | Log |
| shape Maximum2DDiameterColumn | 0.913 | 0.022 | 0.994 | 0.751 | 0.570 | 0.967 | 0.979 | | Log |
| shape Maximum2DDiameterRow | 0.900 | 0.028 | 0.993 | 0.730 | 0.563 | 0.961 | 0.975 | | Log |
| shape Maximum2DDiameterSlice | 0.915 | 0.022 | 0.989 | 0.777 | 0.624 | 0.963 | 0.974 | | - |
| shape Maximum3DDiameter | 0.907 | 0.028 | 0.991 | 0.753 | 0.593 | 0.961 | 0.974 | | - |
| shape MeshVolume | 0.565 | 0.000 | 0.992 | 0.213 | 0.087 | 0.829 | 0.905 | | Log |
| shape MinorAxisLength | 0.903 | 0.022 | 0.992 | 0.739 | 0.563 | 0.960 | 0.974 | | Log |
| shape Sphericity | 0.987 | 0.991 | 0.982 | 0.991 | 0.988 | 0.982 | 0.980 | | - |
| shape SurfaceArea | 0.730 | 0.000 | 0.992 | 0.423 | 0.257 | 0.899 | 0.942 | | Log |
| shape SurfaceVolumeRatio | 0.981 | 0.944 | 0.994 | 0.897 | 0.770 | 0.997 | 0.997 | | Square root |
| shape VoxelVolume | 0.566 | 0.000 | 0.992 | 0.213 | 0.088 | 0.829 | 0.904 | | Log |
| firstorder 10Percentile | 0.988 | 0.022 | 0.605 | 0.912 | 0.789 | 0.933 | 0.803 | | - |
| firstorder 90Percentile | 0.614 | 0.022 | 0.275 | 0.742 | 0.173 | 0.491 | 0.544 | | - |
| firstorder Energy | 0.262 | 0.000 | 0.981 | 0.081 | 0.056 | 0.650 | 0.804 | | - |
| firstorder Entropy | 0.819 | 0.948 | 0.752 | 0.867 | 0.899 | 0.788 | 0.777 | | - |
| firstorder InterquartileRange | 0.983 | 0.000 | 0.827 | 0.953 | 0.833 | 0.934 | 0.905 | | - |
| firstorder Kurtosis | 0.257 | 0.062 | 0.717 | 0.129 | 0.104 | 0.457 | 0.545 | | - |
| firstorder Maximum | 0.254 | 0.000 | 0.598 | 0.136 | 0.116 | 0.448 | 0.561 | | - |
| firstorder MeanAbsoluteDeviation | 0.983 | 0.022 | 0.866 | 0.975 | 0.894 | 0.945 | 0.924 | | - |
| firstorder Mean | 0.944 | 0.022 | 0.569 | 0.987 | 0.485 | 0.866 | 0.842 | | - |
| firstorder Median | 0.914 | 0.023 | 0.572 | 0.947 | 0.447 | 0.855 | 0.841 | | - |
| firstorder Minimum | 0.943 | 0.022 | 0.638 | 0.550 | 0.975 | 0.917 | 0.809 | | - |
| firstorder Range | 0.404 | 0.000 | 0.674 | 0.226 | 0.155 | 0.542 | 0.590 | | - |
| firstorder RobustMeanAbsoluteDeviation | 0.981 | 0.022 | 0.830 | 0.960 | 0.848 | 0.933 | 0.905 | | - |
| firstorder RootMeanSquared | 0.963 | 0.000 | 0.985 | 0.823 | 0.648 | 0.995 | 0.997 | | Square root |
| firstorder Skewness | 0.809 | 0.106 | 0.809 | 0.613 | 0.246 | 0.807 | 0.802 | | - |
| firstorder TotalEnergy | 0.262 | 0.000 | 0.981 | 0.081 | 0.056 | 0.650 | 0.804 | | - |
| firstorder Uniformity | 0.650 | 0.620 | 0.675 | 0.344 | 0.185 | 0.795 | 0.832 | | - |
| firstorder Variance | 0.976 | 0.000 | 0.881 | 0.795 | 0.536 | 0.985 | 0.967 | | - |
| glcm Autocorrelation | 0.963 | 0.000 | 0.708 | 0.989 | 0.957 | 0.882 | 0.836 | | - |
| glcm ClusterProminence | 0.907 | 0.000 | 0.844 | 0.510 | 0.214 | 0.969 | 0.950 | | - |
| glcm ClusterShade | 0.969 | 0.000 | 0.200 | 0.955 | 0.206 | 0.858 | 0.717 | | - |
| glcm ClusterTendency | 0.987 | 0.000 | 0.800 | 0.864 | 0.630 | 0.950 | 0.913 | | - |
| glcm Contrast | 0.983 | 0.038 | 0.885 | 0.832 | 0.628 | 0.985 | 0.965 | | - |
| glcm Correlation | 0.978 | 0.991 | 0.961 | 0.994 | 0.995 | 0.961 | 0.954 | | Cubed |
| glcm DifferenceAverage | 0.994 | 0.231 | 0.892 | 0.960 | 0.867 | 0.965 | 0.945 | | - |
| glcm DifferenceEntropy | 0.874 | 0.988 | 0.796 | 0.927 | 0.960 | 0.838 | 0.825 | | - |
| glcm DifferenceVariance | 0.992 | 0.036 | 0.846 | 0.946 | 0.826 | 0.953 | 0.925 | | - |
| glcm Id | 0.924 | 0.888 | 0.947 | 0.812 | 0.672 | 0.961 | 0.971 | | - |
| glcm Idm | 0.911 | 0.869 | 0.936 | 0.750 | 0.573 | 0.963 | 0.975 | | - |
| glcm Idmn | 0.988 | 0.988 | 0.986 | 0.989 | 0.989 | 0.987 | 0.987 | | - |
| glcm Idn | 0.990 | 0.987 | 0.992 | 0.987 | 0.983 | 0.991 | 0.992 | | Log |
| glcm Imc1 | 0.825 | 0.860 | 0.795 | 0.930 | 0.525 | 0.700 | 0.901 | | - |
| glcm Imc2 | 0.900 | 0.921 | 0.881 | 0.924 | 0.943 | 0.887 | 0.883 | | - |
| glcm InverseVariance | 0.953 | 0.938 | 0.964 | 0.868 | 0.766 | 0.979 | 0.984 | | - |
| glcm JointAverage | 0.906 | 0.123 | 0.744 | 0.974 | 0.992 | 0.838 | 0.810 | | Cubed |
| glcm JointEnergy | 0.496 | 0.478 | 0.513 | 0.178 | 0.106 | 0.783 | 0.858 | | - |
| glcm JointEntropy | 0.886 | 0.939 | 0.813 | 0.935 | 0.964 | 0.853 | 0.841 | | - |
| glcm MCC | 0.953 | 0.973 | 0.928 | 0.979 | 0.988 | 0.930 | 0.921 | | - |
| glcm MaximumProbability | 0.672 | 0.625 | 0.709 | 0.276 | 0.146 | 0.901 | 0.948 | | - |
| glcm SumAverage | 0.906 | 0.049 | 0.744 | 0.974 | 0.992 | 0.838 | 0.810 | | Cubed |
| glcm SumEntropy | 0.861 | 0.976 | 0.803 | 0.903 | 0.932 | 0.834 | 0.824 | | - |
| glcm SumSquares | 0.991 | 0.022 | 0.796 | 0.910 | 0.729 | 0.944 | 0.907 | | - |
| gldm DependenceEntropy | 0.974 | 0.988 | 0.964 | 0.982 | 0.988 | 0.970 | 0.968 | | - |
| gldm DependenceNonUniformity | 0.665 | 0.000 | 0.992 | 0.272 | 0.124 | 0.883 | 0.934 | | Log |
| gldm DependenceNonUniformityNormalized | 0.984 | 0.977 | 0.986 | 0.913 | 0.801 | 0.983 | 0.975 | | - |
| gldm DependenceVariance | 0.817 | 0.022 | 0.973 | 0.435 | 0.180 | 0.957 | 0.979 | | - |
| gldm GrayLevelNonUniformity | 0.285 | 0.000 | 0.970 | 0.051 | 0.028 | 0.663 | 0.795 | | - |
| gldm GrayLevelVariance | 0.976 | 0.022 | 0.742 | 0.962 | 0.853 | 0.904 | 0.861 | | - |
| gldm HighGrayLevelEmphasis | 0.977 | 0.000 | 0.740 | 0.986 | 0.940 | 0.907 | 0.863 | | - |
| gldm LargeDependenceEmphasis | 0.721 | 0.022 | 0.985 | 0.365 | 0.205 | 0.907 | 0.948 | | - |
| gldm LargeDependenceHighGrayLevelEmphasis | 0.778 | 0.000 | 0.978 | 0.383 | 0.155 | 0.947 | 0.977 | | - |
| gldm LargeDependenceLowGrayLevelEmphasis | 0.090 | 0.022 | 0.219 | 0.039 | 0.027 | 0.199 | 0.298 | | - |
| gldm LowGrayLevelEmphasis | 0.193 | 0.175 | 0.209 | 0.055 | 0.033 | 0.530 | 0.692 | | - |
| gldm SmallDependenceEmphasis | 0.978 | 0.993 | 0.958 | 0.986 | 0.937 | 0.929 | 0.902 | | Exp |
| gldm SmallDependenceHighGrayLevelEmphasis | 0.947 | 0.000 | 0.573 | 0.993 | 0.962 | 0.830 | 0.760 | | Squared |
| gldm SmallDependenceLowGrayLevelEmphasis | 0.738 | 0.736 | 0.740 | 0.297 | 0.102 | 0.937 | 0.977 | | - |
| glrlm GrayLevelNonUniformity | 0.375 | 0.000 | 0.976 | 0.096 | 0.042 | 0.709 | 0.824 | | - |
| glrlm GrayLevelNonUniformityNormalized | 0.671 | 0.656 | 0.685 | 0.428 | 0.263 | 0.785 | 0.817 | | - |
| glrlm GrayLevelVariance | 0.966 | 0.024 | 0.744 | 0.981 | 0.915 | 0.890 | 0.850 | | - |
| glrlm HighGrayLevelRunEmphasis | 0.976 | 0.000 | 0.727 | 0.989 | 0.941 | 0.901 | 0.855 | | - |
| glrlm LongRunEmphasis | 0.555 | 0.090 | 0.802 | 0.304 | 0.174 | 0.692 | 0.733 | | - |
| glrlm LongRunHighGrayLevelEmphasis | 0.787 | 0.000 | 0.984 | 0.309 | 0.089 | 0.949 | 0.976 | | - |
| glrlm LongRunLowGrayLevelEmphasis | 0.064 | 0.023 | 0.110 | 0.027 | 0.023 | 0.223 | 0.375 | | - |
| glrlm LowGrayLevelRunEmphasis | 0.476 | 0.470 | 0.483 | 0.157 | 0.077 | 0.772 | 0.859 | | - |
| glrlm RunEntropy | 0.816 | 0.940 | 0.786 | 0.844 | 0.870 | 0.801 | 0.796 | | - |
| glrlm RunLengthNonUniformity | 0.659 | 0.000 | 0.994 | 0.338 | 0.202 | 0.874 | 0.932 | | Log |
| glrlm RunLengthNonUniformityNormalized | 0.883 | 0.914 | 0.855 | 0.921 | 0.946 | 0.858 | 0.849 | | - |
| glrlm RunPercentage | 0.843 | 0.875 | 0.813 | 0.882 | 0.909 | 0.819 | 0.811 | | - |
| glrlm RunVariance | 0.486 | 0.116 | 0.701 | 0.173 | 0.104 | 0.799 | 0.885 | | - |
| glrlm ShortRunEmphasis | 0.834 | 0.857 | 0.813 | 0.860 | 0.881 | 0.820 | 0.815 | | - |
| glrlm ShortRunHighGrayLevelEmphasis | 0.939 | 0.000 | 0.640 | 0.991 | 0.976 | 0.837 | 0.782 | | - |
| glrlm ShortRunLowGrayLevelEmphasis | 0.676 | 0.673 | 0.678 | 0.343 | 0.188 | 0.876 | 0.929 | | - |
| glszm GrayLevelNonUniformity | 0.697 | 0.022 | 0.992 | 0.389 | 0.241 | 0.885 | 0.935 | | Log |
| glszm GrayLevelNonUniformityNormalized | 0.539 | 0.533 | 0.545 | 0.375 | 0.262 | 0.629 | 0.659 | | - |
| glszm GrayLevelVariance | 0.977 | 0.022 | 0.885 | 0.992 | 0.956 | 0.943 | 0.926 | | Squared |
| glszm HighGrayLevelZoneEmphasis | 0.958 | 0.000 | 0.736 | 0.986 | 0.931 | 0.878 | 0.838 | | - |
| glszm LargeAreaEmphasis | 0.112 | 0.000 | 0.954 | 0.029 | 0.023 | 0.445 | 0.650 | | - |
| glszm LargeAreaHighGrayLevelEmphasis | 0.072 | 0.000 | 0.979 | 0.024 | 0.022 | 0.423 | 0.673 | | - |
| glszm LargeAreaLowGrayLevelEmphasis | 0.050 | 0.000 | 0.441 | 0.025 | 0.022 | 0.184 | 0.357 | | - |
| glszm LowGrayLevelZoneEmphasis | 0.559 | 0.554 | 0.564 | 0.212 | 0.112 | 0.810 | 0.878 | | - |
| glszm SizeZoneNonUniformity | 0.717 | 0.000 | 0.997 | 0.293 | 0.122 | 0.919 | 0.961 | | Log |
| glszm SizeZoneNonUniformityNormalized | 0.919 | 0.945 | 0.892 | 0.966 | 0.990 | 0.884 | 0.870 | | - |
| glszm SmallAreaEmphasis | 0.870 | 0.897 | 0.844 | 0.907 | 0.936 | 0.849 | 0.841 | | - |
| glszm SmallAreaHighGrayLevelEmphasis | 0.951 | 0.000 | 0.729 | 0.996 | 0.969 | 0.869 | 0.829 | | Squared |
| glszm SmallAreaLowGrayLevelEmphasis | 0.661 | 0.658 | 0.664 | 0.295 | 0.150 | 0.868 | 0.920 | | - |
| glszm ZoneEntropy | 0.982 | 0.914 | 0.986 | 0.976 | 0.969 | 0.984 | 0.985 | | - |
| glszm ZonePercentage | 0.974 | 0.991 | 0.950 | 0.984 | 0.939 | 0.920 | 0.891 | | - |
| glszm ZoneVariance | 0.111 | 0.000 | 0.970 | 0.029 | 0.023 | 0.448 | 0.664 | | - |
| ngtdm Busyness | 0.409 | 0.032 | 0.634 | 0.068 | 0.029 | 0.747 | 0.836 | | - |
| ngtdm Coarseness | 0.822 | 0.820 | 0.825 | 0.493 | 0.289 | 0.967 | 0.991 | | - |
| ngtdm Complexity | 0.989 | 0.000 | 0.892 | 0.933 | 0.802 | 0.965 | 0.946 | | - |
| ngtdm Contrast | 0.987 | 0.953 | 0.989 | 0.863 | 0.699 | 0.970 | 0.934 | | - |

Supplementary Table S3 – Shapiro-Wilk test statistic W of radiomic feature distribution subjected to different transformations. The selected transformation was **none** if the Shapiro-Wilk test is nonsignificant for the non-transformed distribution, the transformation which gave a nonsignificant Shapiro-Wilk test and the higher W in case of several transformation with nonsignificant test, and **none** if no transformation was nonsignificant.

| **Group** | **Brest** | **Liege** | **pValue** |
| --- | --- | --- | --- |
| Observations |  |  |  |
|  | 33 | 401 |  |
| Age |  |  |  |
| Mean (SD) | 72 (10) | 71 (9.7) | 0.68 |
| Gender |  |  |  |
| Male | 82% (27) | 61% (244) | 0.028 |
| Female | 18% (6) | 39% (157) |  |
| PS |  |  |  |
| 0 | 36% (12) | 17% (67) | 0.035 |
| 1 | 52% (17) | 60% (239) |  |
| 2 | 12% (4) | 23% (91) |  |
| 3 | 0% (0) | 0.75% (3) |  |
| Tobacco |  |  |  |
| No | 15% (5) | 5.2% (21) | 0.054 |
| Yes | 85% (28) | 95% (380) |  |
| Previous thoracic radiation therapy |  |  |  |
| No | 88% (29) | 96% (384) | 0.11 |
| Yes | 12% (4) | 4.2% (17) |  |
| Previous thoracic surgery |  |  |  |
| No | 82% (27) | 91% (364) | 0.18 |
| Yes | 18% (6) | 9.2% (37) |  |
| Operability |  |  |  |
| No | 97% (32) | 95% (380) | 0.89 |
| Yes | 3% (1) | 5.2% (21) |  |
| Histology |  |  |  |
|  | 9.1% (3) | 0% (0) | <0.001 |
| carcinome epidermoïde | 3% (1) | 0% (0) |  |
| adenocarcinome | 6.1% (2) | 0% (0) |  |
| adenocarcinome | 3% (1) | 0% (0) |  |
| adenocarcinome lepidique et acinaire | 3% (1) | 0% (0) |  |
| adenocarcinome TTF1+ | 3% (1) | 0% (0) |  |
| ADK | 36% (12) | 42% (170) |  |
| carcinome a grandes cellules | 0% (0) | 0.75% (3) |  |
| carcinome adeno-squameux | 0% (0) | 0.25% (1) |  |
| Carcinome bronchiolo-alveolaire de type non mucineux | 0% (0) | 0.25% (1) |  |
| carcinome indifferencie | 3% (1) | 0% (0) |  |
| Carcinome indifferencie | 0% (0) | 0.25% (1) |  |
| carcinome NOS | 0% (0) | 1% (4) |  |
| Carcinome NOS | 0% (0) | 0.75% (3) |  |
| carcinome peu differencie | 0% (0) | 0.5% (2) |  |
| Carcinome peu differencie | 0% (0) | 0.25% (1) |  |
| carcinome peu differencie a grandes cellules | 0% (0) | 0.25% (1) |  |
| CE | 24% (8) | 34% (138) |  |
| NaN | 9.1% (3) | 18% (74) |  |
| NSCLC | 0% (0) | 0.25% (1) |  |
| tumeur carcinoide | 0% (0) | 0.25% (1) |  |
| Tumor stage |  |  |  |
| T1a | 12% (4) | 13% (54) | 0.52 |
| T1b | 42% (14) | 50% (201) |  |
| T1c | 33% (11) | 23% (91) |  |
| T2a | 12% (4) | 10% (40) |  |
| T2b | 0% (0) | 3.7% (15) |  |
| missing | 0% (0) | 0% (0) |  |
| TDM diameter in mm |  |  |  |
| Mean (SD) | 21 (8.4) | 18 (8.9) | 0.18 |
| GTV volume in cc |  |  |  |
| Mean (SD) | 7.7 (7.4) | 11 (13) | 0.6 |
| PTV volume in cc |  |  |  |
| Mean (SD) | 32 (26) | 27 (25) | 0.044 |
| Recurrence |  |  |  |
| No | 73% (24) | 63% (251) | 0.33 |
| Yes | 27% (9) | 37% (150) |  |
| Regional recurrence |  |  |  |
| No | 88% (29) | 83% (333) | 0.64 |
| Yes | 12% (4) | 17% (68) |  |
| Distant recurrence |  |  |  |
| No | 82% (27) | 73% (294) | 0.39 |
| Yes | 18% (6) | 27% (107) |  |
| Regional or distant recurrence |  |  |  |
| No | 76% (25) | 67% (267) | 0.38 |
| Yes | 24% (8) | 33% (134) |  |
| Three year post RT regional or distant recurrence |  |  |  |
| No | 79% (26) | 74% (295) | 0.65 |
| Yes | 21% (7) | 26% (106) |  |
| Cancer specific death |  |  |  |
| No | 85% (28) | 74% (295) | 0.22 |
| Yes | 15% (5) | 26% (106) |  |

Supplementary Table S4 – Patient characteristics by center.

|  | **Center A** | | | | | **Center B** | | | | |
| --- | --- | --- | --- | --- | --- | --- | --- | --- | --- | --- |
| **feature** | **AUC** | **OR** | **CI** | **p-val** | **sign** | **AUC** | **OR** | **CI** | **p-val** | **sign** |
| Robust shape LeastAxisLength | 0.549931 | 1.042 | [0.998-1.089] | 0.063709 |  | 0.59867 | 1.059 | [0.944-1.188] | 0.330365 |  |
| Robust shape Maximum2DDiameterSlice | 0.566047 | 1.046 | [1.001-1.093] | 0.043404 | * | 0.592018 | 1.083 | [0.967-1.214] | 0.175137 |  |
| Robust shape MinorAxisLength | 0.565761 | 1.056 | [1.011-1.103] | 0.01477 | * | 0.594235 | 1.056 | [0.944-1.181] | 0.344861 |  |
| Robust shape SurfaceArea | 0.563495 | 1.053 | [1.008-1.1] | 0.020887 | * | 0.607539 | 1.071 | [0.955-1.201] | 0.247621 |  |
| Robust shape SurfaceVolumeRatio | 0.564585 | 0.944 | [0.904-0.986] | 0.010176 | * | 0.59867 | 0.939 | [0.837-1.053] | 0.285562 |  |
| Robust glcm DifferenceAverage | 0.553789 | 0.947 | [0.907-0.989] | 0.014986 | * | 0.614191 | 0.923 | [0.82-1.039] | 0.189327 |  |
| Robust glcm Id | 0.56808 | 1.075 | [1.03-1.123] | 0.001045 | ** | 0.580931 | 1.046 | [0.928-1.178] | 0.464801 |  |
| Robust glcm Idm | 0.570329 | 1.077 | [1.031-1.124] | 0.000865 | *** | 0.574279 | 1.037 | [0.92-1.169] | 0.554613 |  |
| Robust glcm Idn | 0.555934 | 1.057 | [1.012-1.104] | 0.01216 | * | 0.618625 | 1.082 | [0.96-1.219] | 0.20375 |  |
| Robust glcm InverseVariance | 0.553201 | 1.053 | [1.008-1.1] | 0.019929 | * | 0.572062 | 1.04 | [0.927-1.168] | 0.506505 |  |
| Robust gldm DependenceNonUniformityNormalized | 0.567266 | 0.944 | [0.904-0.985] | 0.009032 | ** | 0.583149 | 0.946 | [0.84-1.066] | 0.369152 |  |
| Robust gldm GrayLevelNonUniformity | 0.567457 | 1.062 | [1.017-1.109] | 0.006534 | ** | 0.572062 | 1.024 | [0.873-1.202] | 0.771348 |  |
| Robust gldm SmallDependenceEmphasis | 0.564913 | 0.938 | [0.898-0.98] | 0.004083 | ** | 0.62306 | 0.926 | [0.823-1.042] | 0.206679 |  |
| Robust glrlm GrayLevelNonUniformity | 0.566332 | 1.058 | [1.013-1.105] | 0.011633 | * | 0.569845 | 1.019 | [0.904-1.149] | 0.760475 |  |
| Robust glrlm RunLengthNonUniformityNormalized | 0.571228 | 0.929 | [0.89-0.97] | 0.000909 | *** | 0.56541 | 0.969 | [0.858-1.094] | 0.615661 |  |
| Robust glrlm RunPercentage | 0.579291 | 0.921 | [0.882-0.961] | 0.00019 | *** | 0.56541 | 0.969 | [0.857-1.095] | 0.616041 |  |
| Robust glrlm ShortRunEmphasis | 0.57192 | 0.926 | [0.887-0.967] | 0.000573 | *** | 0.56541 | 0.971 | [0.861-1.096] | 0.64036 |  |
| Robust glszm GrayLevelNonUniformity | 0.550173 | 1.042 | [0.998-1.089] | 0.061871 |  | 0.600887 | 1.066 | [0.949-1.198] | 0.285903 |  |
| Robust glszm ZonePercentage | 0.563737 | 0.937 | [0.898-0.979] | 0.003547 | ** | 0.611973 | 0.938 | [0.835-1.054] | 0.286962 |  |
| HD nonrobust shape Elongation | 0.514 | 0.985 | [0.943-1.029] | 0.512 |  | 0.534 | 0.974 | [0.869-1.091] | 0.649 |  |
| HD nonrobust shape Flatness | 0.533 | 0.974 | [0.933-1.018] | 0.246 |  | 0.523 | 0.976 | [0.87-1.094] | 0.675 |  |
| HD nonrobust shape MajorAxisLength | 0.571 | 1.062 | [1.017-1.109] | 0.007 | ** | 0.656 | 1.075 | [0.959-1.204] | 0.221 |  |
| HD nonrobust shape Maximum2DDiameterColumn | 0.562 | 1.054 | [1.009-1.101] | 0.017 | * | 0.590 | 1.058 | [0.943-1.187] | 0.345 |  |
| HD nonrobust shape Maximum2DDiameterRow | 0.573 | 1.059 | [1.014-1.106] | 0.010 | ** | 0.611 | 1.062 | [0.943-1.195] | 0.328 |  |
| HD nonrobust shape Maximum3DDiameter | 0.565 | 1.053 | [1.008-1.099] | 0.021 | * | 0.625 | 1.065 | [0.948-1.197] | 0.296 |  |
| HD nonrobust shape MeshVolume | 0.564 | 1.056 | [1.011-1.103] | 0.014 | * | 0.599 | 1.07 | [0.954-1.199] | 0.255 |  |
| HD nonrobust shape Sphericity | 0.493 | 0.996 | [0.953-1.04] | 0.841 |  | 0.539 | 0.961 | [0.849-1.088] | 0.533 |  |
| HD nonrobust shape VoxelVolume | 0.564 | 1.056 | [1.011-1.103] | 0.014 | * | 0.600 | 1.07 | [0.954-1.199] | 0.254 |  |
| HD nonrobust firstorder 10Percentile | 0.547 | 1.036 | [0.992-1.082] | 0.110 |  | 0.563 | 0.949 | [0.841-1.07] | 0.397 |  |
| HD nonrobust firstorder 90Percentile | 0.518 | 1.032 | [0.988-1.078] | 0.158 |  | 0.554 | 0.975 | [0.87-1.093] | 0.669 |  |
| HD nonrobust firstorder Energy | 0.534 | 1.02 | [0.976-1.065] | 0.385 |  | 0.650 | 1.111 | [0.988-1.25] | 0.084 |  |
| HD nonrobust firstorder Entropy | 0.552 | 0.936 | [0.896-0.978] | 0.003 | ** | 0.490 | 1.038 | [0.921-1.169] | 0.544 |  |
| HD nonrobust firstorder InterquartileRange | 0.530 | 0.967 | [0.926-1.011] | 0.137 |  | 0.539 | 1.036 | [0.918-1.169] | 0.573 |  |
| HD nonrobust firstorder Kurtosis | 0.544 | 1.084 | [1.039-1.132] | 0.000 | *** | 0.443 | 0.939 | [0.827-1.067] | 0.338 |  |
| HD nonrobust firstorder Maximum | 0.556 | 1.092 | [1.046-1.14] | 0.000 | *** | 0.608 | 0.937 | [0.799-1.1] | 0.434 |  |
| HD nonrobust firstorder MeanAbsoluteDeviation | 0.485 | 0.977 | [0.936-1.021] | 0.303 |  | 0.543 | 1.028 | [0.909-1.162] | 0.663 |  |
| HD nonrobust firstorder Mean | 0.552 | 1.046 | [1.001-1.092] | 0.044 | * | 0.552 | 0.962 | [0.85-1.088] | 0.539 |  |
| HD nonrobust firstorder Median | 0.549 | 1.046 | [1.002-1.093] | 0.043 | * | 0.548 | 0.968 | [0.855-1.096] | 0.610 |  |
| HD nonrobust firstorder Minimum | 0.515 | 1.02 | [0.976-1.065] | 0.380 |  | 0.532 | 0.988 | [0.875-1.115] | 0.842 |  |
| HD nonrobust firstorder Range | 0.528 | 1.083 | [1.037-1.131] | 0.000 | *** | 0.512 | 0.972 | [0.848-1.115] | 0.688 |  |
| HD nonrobust firstorder RobustMeanAbsoluteDeviation | 0.524 | 0.971 | [0.93-1.014] | 0.189 |  | 0.545 | 1.035 | [0.916-1.17] | 0.578 |  |
| HD nonrobust firstorder RootMeanSquared | 0.543 | 0.96 | [0.919-1.002] | 0.065 |  | 0.563 | 1.049 | [0.932-1.18] | 0.431 |  |
| HD nonrobust firstorder Skewness | 0.513 | 1.027 | [0.983-1.073] | 0.233 |  | 0.561 | 1.044 | [0.925-1.179] | 0.486 |  |
| HD nonrobust firstorder TotalEnergy | 0.534 | 1.02 | [0.976-1.065] | 0.385 |  | 0.650 | 1.111 | [0.988-1.25] | 0.084 |  |
| HD nonrobust firstorder Uniformity | 0.557 | 1.076 | [1.031-1.124] | 0.001 | *** | 0.532 | 0.965 | [0.856-1.088] | 0.562 |  |
| HD nonrobust firstorder Variance | 0.504 | 0.994 | [0.951-1.038] | 0.774 |  | 0.534 | 1.026 | [0.903-1.166] | 0.699 |  |
| HD nonrobust glcm Autocorrelation | 0.508 | 0.98 | [0.938-1.024] | 0.365 |  | 0.463 | 0.968 | [0.863-1.087] | 0.588 |  |
| HD nonrobust glcm ClusterProminence | 0.519 | 0.98 | [0.938-1.024] | 0.363 |  | 0.585 | 1.065 | [0.948-1.197] | 0.292 |  |
| HD nonrobust glcm ClusterShade | 0.508 | 0.99 | [0.947-1.034] | 0.640 |  | 0.534 | 0.988 | [0.883-1.106] | 0.839 |  |
| HD nonrobust glcm ClusterTendency | 0.534 | 0.962 | [0.921-1.005] | 0.082 |  | 0.583 | 1.078 | [0.953-1.219] | 0.239 |  |
| HD nonrobust glcm Contrast | 0.546 | 0.958 | [0.917-1] | 0.052 |  | 0.621 | 0.917 | [0.817-1.03] | 0.151 |  |
| HD nonrobust glcm Correlation | 0.508 | 1 | [0.957-1.045] | 0.999 |  | 0.705 | 1.129 | [1.012-1.26] | 0.034 | * |
| HD nonrobust glcm DifferenceEntropy | 0.552 | 0.937 | [0.897-0.979] | 0.004 | ** | 0.605 | 0.933 | [0.828-1.05] | 0.254 |  |
| HD nonrobust glcm DifferenceVariance | 0.534 | 0.964 | [0.923-1.007] | 0.098 |  | 0.630 | 0.921 | [0.819-1.035] | 0.174 |  |
| HD nonrobust glcm Idmn | 0.548 | 1.046 | [1.001-1.092] | 0.045 | * | 0.621 | 1.091 | [0.97-1.226] | 0.152 |  |
| HD nonrobust glcm Imc1 | 0.521 | 1.025 | [0.982-1.071] | 0.260 |  | 0.625 | 0.957 | [0.853-1.074] | 0.460 |  |
| HD nonrobust glcm Imc2 | 0.564 | 0.949 | [0.909-0.991] | 0.019 | * | 0.645 | 1.077 | [0.951-1.219] | 0.246 |  |
| HD nonrobust glcm JointAverage | 0.506 | 0.989 | [0.947-1.034] | 0.630 |  | 0.545 | 0.962 | [0.857-1.078] | 0.507 |  |
| HD nonrobust glcm JointEnergy | 0.575 | 1.085 | [1.039-1.132] | 0.000 | *** | 0.492 | 0.977 | [0.865-1.104] | 0.710 |  |
| HD nonrobust glcm JointEntropy | 0.548 | 0.942 | [0.902-0.983] | 0.007 | ** | 0.483 | 1.032 | [0.915-1.163] | 0.613 |  |
| HD nonrobust glcm MCC | 0.513 | 0.998 | [0.955-1.042] | 0.916 |  | 0.703 | 1.13 | [1.008-1.267] | 0.041 | * |
| HD nonrobust glcm MaximumProbability | 0.588 | 1.094 | [1.048-1.142] | 0.000 | *** | 0.528 | 0.995 | [0.882-1.122] | 0.934 |  |
| HD nonrobust glcm SumAverage | 0.506 | 0.989 | [0.947-1.034] | 0.630 |  | 0.545 | 0.962 | [0.857-1.078] | 0.507 |  |
| HD nonrobust glcm SumEntropy | 0.550 | 0.939 | [0.899-0.98] | 0.004 | ** | 0.557 | 1.054 | [0.934-1.188] | 0.399 |  |
| HD nonrobust glcm SumSquares | 0.535 | 0.96 | [0.919-1.003] | 0.065 |  | 0.572 | 1.051 | [0.931-1.187] | 0.426 |  |
| HD nonrobust gldm DependenceEntropy | 0.527 | 1.012 | [0.969-1.057] | 0.599 |  | 0.692 | 1.115 | [0.997-1.247] | 0.063 |  |
| HD nonrobust gldm DependenceNonUniformity | 0.540 | 1.035 | [0.991-1.081] | 0.120 |  | 0.625 | 1.071 | [0.958-1.196] | 0.233 |  |
| HD nonrobust gldm DependenceVariance | 0.592 | 1.093 | [1.047-1.141] | 0.000 | *** | 0.528 | 1.026 | [0.911-1.154] | 0.676 |  |
| HD nonrobust gldm GrayLevelVariance | 0.531 | 0.958 | [0.917-1] | 0.052 |  | 0.557 | 1.039 | [0.925-1.168] | 0.522 |  |
| HD nonrobust gldm HighGrayLevelEmphasis | 0.498 | 0.985 | [0.943-1.029] | 0.506 |  | 0.477 | 0.971 | [0.865-1.09] | 0.620 |  |
| HD nonrobust gldm LargeDependenceEmphasis | 0.587 | 1.095 | [1.049-1.143] | 0.000 | *** | 0.541 | 1.025 | [0.905-1.16] | 0.699 |  |
| HD nonrobust gldm LargeDependenceHighGrayLevelEmphasis | 0.541 | 1.038 | [0.994-1.085] | 0.090 |  | 0.475 | 1.017 | [0.902-1.147] | 0.786 |  |
| HD nonrobust gldm LargeDependenceLowGrayLevelEmphasis | 0.537 | 1.046 | [1.001-1.093] | 0.043 | * | 0.565 | 1.581 | [0.673-3.711] | 0.298 |  |
| HD nonrobust gldm LowGrayLevelEmphasis | 0.518 | 1.046 | [1.002-1.093] | 0.043 | * | 0.501 | 0.964 | [0.836-1.111] | 0.611 |  |
| HD nonrobust gldm SmallDependenceHighGrayLevelEmphasis | 0.545 | 0.953 | [0.913-0.996] | 0.032 | * | 0.647 | 0.922 | [0.824-1.033] | 0.167 |  |
| HD nonrobust gldm SmallDependenceLowGrayLevelEmphasis | 0.533 | 0.952 | [0.911-0.994] | 0.026 | * | 0.552 | 0.958 | [0.855-1.073] | 0.462 |  |
| HD nonrobust glrlm GrayLevelNonUniformityNormalized | 0.544 | 1.068 | [1.023-1.115] | 0.003 | ** | 0.530 | 0.956 | [0.849-1.078] | 0.467 |  |
| HD nonrobust glrlm GrayLevelVariance | 0.469 | 0.955 | [0.915-0.998] | 0.040 | * | 0.545 | 1.036 | [0.922-1.163] | 0.555 |  |
| HD nonrobust glrlm HighGrayLevelRunEmphasis | 0.505 | 0.984 | [0.942-1.028] | 0.465 |  | 0.461 | 0.966 | [0.861-1.084] | 0.557 |  |
| HD nonrobust glrlm LongRunEmphasis | 0.583 | 1.096 | [1.05-1.144] | 0.000 | *** | 0.568 | 1.029 | [0.904-1.173] | 0.664 |  |
| HD nonrobust glrlm LongRunHighGrayLevelEmphasis | 0.521 | 1.033 | [0.989-1.079] | 0.141 |  | 0.501 | 0.995 | [0.883-1.122] | 0.939 |  |
| HD nonrobust glrlm LongRunLowGrayLevelEmphasis | 0.505 | 1.04 | [0.995-1.086] | 0.082 |  | 0.537 | 0.926 | [0.606-1.415] | 0.723 |  |
| HD nonrobust glrlm LowGrayLevelRunEmphasis | 0.515 | 1.039 | [0.995-1.086] | 0.083 |  | 0.508 | 0.969 | [0.855-1.097] | 0.618 |  |
| HD nonrobust glrlm RunEntropy | 0.514 | 0.958 | [0.918-1.001] | 0.056 |  | 0.652 | 1.1 | [0.977-1.24] | 0.122 |  |
| HD nonrobust glrlm RunLengthNonUniformity | 0.555 | 1.046 | [1.002-1.093] | 0.043 | * | 0.612 | 1.07 | [0.957-1.197] | 0.239 |  |
| HD nonrobust glrlm RunVariance | 0.586 | 1.097 | [1.051-1.145] | 0.000 | *** | 0.563 | 1.034 | [0.896-1.193] | 0.648 |  |
| HD nonrobust glrlm ShortRunHighGrayLevelEmphasis | 0.516 | 0.972 | [0.931-1.015] | 0.204 |  | 0.554 | 0.958 | [0.855-1.074] | 0.463 |  |
| HD nonrobust glrlm ShortRunLowGrayLevelEmphasis | 0.516 | 1.015 | [0.972-1.061] | 0.493 |  | 0.503 | 0.968 | [0.856-1.093] | 0.600 |  |
| HD nonrobust glszm GrayLevelNonUniformityNormalized | 0.514 | 1.069 | [1.023-1.116] | 0.003 | ** | 0.537 | 0.988 | [0.881-1.108] | 0.837 |  |
| HD nonrobust glszm GrayLevelVariance | 0.531 | 0.965 | [0.924-1.008] | 0.108 |  | 0.452 | 0.965 | [0.861-1.082] | 0.544 |  |
| HD nonrobust glszm HighGrayLevelZoneEmphasis | 0.512 | 0.974 | [0.933-1.018] | 0.245 |  | 0.563 | 0.946 | [0.845-1.06] | 0.345 |  |
| HD nonrobust glszm LargeAreaEmphasis | 0.577 | 1.055 | [1.01-1.102] | 0.016 | * | 0.568 | 1.005 | [0.896-1.127] | 0.932 |  |
| HD nonrobust glszm LargeAreaHighGrayLevelEmphasis | 0.556 | 1.039 | [0.995-1.085] | 0.087 |  | 0.552 | 1.004 | [0.899-1.121] | 0.950 |  |
| HD nonrobust glszm LargeAreaLowGrayLevelEmphasis | 0.566 | 1.038 | [0.994-1.085] | 0.091 |  | 0.588 | 1.183 | [0.56-2.499] | 0.662 |  |
| HD nonrobust glszm LowGrayLevelZoneEmphasis | 0.508 | 1.027 | [0.983-1.073] | 0.233 |  | 0.519 | 0.982 | [0.868-1.11] | 0.769 |  |
| HD nonrobust glszm SizeZoneNonUniformity | 0.535 | 1.021 | [0.978-1.067] | 0.347 |  | 0.563 | 1.045 | [0.93-1.173] | 0.463 |  |
| HD nonrobust glszm SizeZoneNonUniformityNormalized | 0.566 | 0.941 | [0.901-0.982] | 0.006 | ** | 0.659 | 0.897 | [0.8-1.005] | 0.066 |  |
| HD nonrobust glszm SmallAreaEmphasis | 0.566 | 0.939 | [0.9-0.981] | 0.005 | ** | 0.661 | 0.9 | [0.808-1.003] | 0.062 |  |
| HD nonrobust glszm SmallAreaHighGrayLevelEmphasis | 0.516 | 0.978 | [0.936-1.021] | 0.311 |  | 0.616 | 0.932 | [0.836-1.038] | 0.204 |  |
| HD nonrobust glszm SmallAreaLowGrayLevelEmphasis | 0.506 | 1.01 | [0.967-1.055] | 0.659 |  | 0.497 | 0.974 | [0.866-1.095] | 0.663 |  |
| HD nonrobust glszm ZoneEntropy | 0.533 | 1.022 | [0.978-1.068] | 0.325 |  | 0.659 | 1.113 | [0.992-1.248] | 0.074 |  |
| HD nonrobust glszm ZoneVariance | 0.577 | 1.055 | [1.01-1.102] | 0.016 | * | 0.554 | 1.005 | [0.896-1.127] | 0.935 |  |
| HD nonrobust ngtdm Busyness | 0.567 | 1.068 | [1.023-1.115] | 0.003 | ** | 0.590 | 1.057 | [0.933-1.198] | 0.388 |  |
| HD nonrobust ngtdm Coarseness | 0.569 | 0.956 | [0.916-0.999] | 0.045 | * | 0.552 | 0.981 | [0.875-1.099] | 0.742 |  |
| HD nonrobust ngtdm Complexity | 0.554 | 0.946 | [0.906-0.988] | 0.013 | * | 0.705 | 0.885 | [0.793-0.987] | 0.033 | * |
| HD nonrobust ngtdm Contrast | 0.546 | 0.958 | [0.917-1] | 0.052 |  | 0.559 | 0.94 | [0.836-1.058] | 0.312 |  |
| LD nonrobust shape Elongation | 0.533 | 0.974 | [0.933-1.018] | 0.242 |  | 0.514 | 1.008 | [0.901-1.127] | 0.895 |  |
| LD nonrobust shape Flatness | 0.565 | 0.946 | [0.906-0.989] | 0.013 | * | 0.627 | 0.964 | [0.859-1.08] | 0.528 |  |
| LD nonrobust shape MajorAxisLength | 0.549 | 1.05 | [1.005-1.097] | 0.028 | * | 0.590 | 1.051 | [0.936-1.18] | 0.405 |  |
| LD nonrobust shape Maximum2DDiameterColumn | 0.537 | 1.044 | [0.999-1.09] | 0.054 |  | 0.563 | 1.046 | [0.933-1.173] | 0.441 |  |
| LD nonrobust shape Maximum2DDiameterRow | 0.544 | 1.05 | [1.005-1.096] | 0.029 | * | 0.559 | 1.051 | [0.936-1.18] | 0.401 |  |
| LD nonrobust shape Maximum3DDiameter | 0.539 | 1.045 | [1.001-1.092] | 0.048 | * | 0.595 | 1.073 | [0.957-1.203] | 0.231 |  |
| LD nonrobust shape MeshVolume | 0.525 | 1.055 | [1.01-1.102] | 0.016 | * | 0.581 | 1.054 | [0.94-1.182] | 0.368 |  |
| LD nonrobust shape Sphericity | 0.516 | 0.984 | [0.942-1.028] | 0.469 |  | 0.568 | 0.967 | [0.866-1.079] | 0.546 |  |
| LD nonrobust shape VoxelVolume | 0.525 | 1.055 | [1.01-1.102] | 0.016 | * | 0.581 | 1.054 | [0.94-1.182] | 0.368 |  |
| LD nonrobust firstorder 10Percentile | 0.519 | 1.015 | [0.971-1.06] | 0.508 |  | 0.528 | 1.008 | [0.9-1.129] | 0.895 |  |
| LD nonrobust firstorder 90Percentile | 0.539 | 1.006 | [0.963-1.051] | 0.796 |  | 0.543 | 0.999 | [0.859-1.162] | 0.989 |  |
| LD nonrobust firstorder Energy | 0.517 | 1.042 | [0.997-1.088] | 0.065 |  | 0.623 | 1.086 | [0.97-1.216] | 0.157 |  |
| LD nonrobust firstorder Entropy | 0.546 | 0.942 | [0.902-0.984] | 0.007 | ** | 0.532 | 0.983 | [0.878-1.101] | 0.770 |  |
| LD nonrobust firstorder InterquartileRange | 0.514 | 0.986 | [0.944-1.03] | 0.535 |  | 0.550 | 0.981 | [0.879-1.096] | 0.740 |  |
| LD nonrobust firstorder Kurtosis | 0.480 | 1.049 | [1.005-1.096] | 0.031 | * | 0.463 | 1.014 | [0.896-1.148] | 0.826 |  |
| LD nonrobust firstorder Maximum | 0.547 | 1.056 | [1.011-1.103] | 0.015 | * | 0.514 | 1.017 | [0.881-1.174] | 0.816 |  |
| LD nonrobust firstorder MeanAbsoluteDeviation | 0.499 | 0.997 | [0.954-1.041] | 0.876 |  | 0.477 | 0.989 | [0.885-1.106] | 0.848 |  |
| LD nonrobust firstorder Mean | 0.528 | 1.012 | [0.969-1.057] | 0.591 |  | 0.514 | 1.005 | [0.887-1.139] | 0.942 |  |
| LD nonrobust firstorder Median | 0.523 | 1.01 | [0.967-1.055] | 0.654 |  | 0.477 | 1.007 | [0.89-1.139] | 0.915 |  |
| LD nonrobust firstorder Minimum | 0.496 | 1.004 | [0.961-1.049] | 0.870 |  | 0.568 | 0.964 | [0.859-1.082] | 0.541 |  |
| LD nonrobust firstorder Range | 0.537 | 1.051 | [1.007-1.098] | 0.024 | * | 0.568 | 1.044 | [0.908-1.2] | 0.548 |  |
| LD nonrobust firstorder RobustMeanAbsoluteDeviation | 0.508 | 0.99 | [0.948-1.035] | 0.662 |  | 0.543 | 0.984 | [0.881-1.1] | 0.778 |  |
| LD nonrobust firstorder RootMeanSquared | 0.521 | 0.993 | [0.951-1.037] | 0.753 |  | 0.528 | 0.999 | [0.884-1.128] | 0.982 |  |
| LD nonrobust firstorder Skewness | 0.503 | 1.01 | [0.967-1.055] | 0.658 |  | 0.481 | 0.984 | [0.878-1.103] | 0.783 |  |
| LD nonrobust firstorder TotalEnergy | 0.517 | 1.042 | [0.997-1.088] | 0.065 |  | 0.623 | 1.086 | [0.97-1.216] | 0.157 |  |
| LD nonrobust firstorder Uniformity | 0.554 | 1.05 | [1.005-1.097] | 0.029 | * | 0.537 | 1.022 | [0.912-1.145] | 0.708 |  |
| LD nonrobust firstorder Variance | 0.514 | 1.01 | [0.967-1.055] | 0.659 |  | 0.497 | 0.99 | [0.884-1.108] | 0.864 |  |
| LD nonrobust glcm Autocorrelation | 0.507 | 0.982 | [0.94-1.026] | 0.409 |  | 0.486 | 1.007 | [0.893-1.137] | 0.905 |  |
| LD nonrobust glcm ClusterProminence | 0.503 | 0.999 | [0.956-1.043] | 0.947 |  | 0.459 | 0.969 | [0.862-1.09] | 0.604 |  |
| LD nonrobust glcm ClusterShade | 0.494 | 1.009 | [0.966-1.054] | 0.698 |  | 0.479 | 1.012 | [0.9-1.138] | 0.839 |  |
| LD nonrobust glcm ClusterTendency | 0.507 | 0.976 | [0.934-1.019] | 0.272 |  | 0.477 | 0.981 | [0.877-1.096] | 0.732 |  |
| LD nonrobust glcm Contrast | 0.531 | 0.967 | [0.926-1.01] | 0.133 |  | 0.594 | 0.944 | [0.847-1.053] | 0.305 |  |
| LD nonrobust glcm Correlation | 0.509 | 1.004 | [0.961-1.049] | 0.850 |  | 0.630 | 1.085 | [0.967-1.219] | 0.171 |  |
| LD nonrobust glcm DifferenceEntropy | 0.538 | 0.961 | [0.92-1.003] | 0.072 |  | 0.592 | 0.945 | [0.847-1.054] | 0.315 |  |
| LD nonrobust glcm DifferenceVariance | 0.480 | 0.981 | [0.939-1.025] | 0.396 |  | 0.608 | 0.923 | [0.816-1.044] | 0.206 |  |
| LD nonrobust glcm Idmn | 0.531 | 1.026 | [0.982-1.072] | 0.254 |  | 0.590 | 1.064 | [0.953-1.188] | 0.276 |  |
| LD nonrobust glcm Imc1 | 0.518 | 0.973 | [0.931-1.016] | 0.220 |  | 0.510 | 1.025 | [0.916-1.147] | 0.672 |  |
| LD nonrobust glcm Imc2 | 0.530 | 0.974 | [0.933-1.018] | 0.240 |  | 0.486 | 0.988 | [0.885-1.104] | 0.838 |  |
| LD nonrobust glcm JointAverage | 0.494 | 0.984 | [0.941-1.027] | 0.457 |  | 0.494 | 1.008 | [0.895-1.134] | 0.902 |  |
| LD nonrobust glcm JointEnergy | 0.566 | 1.038 | [0.994-1.084] | 0.094 |  | 0.537 | 1.041 | [0.925-1.172] | 0.509 |  |
| LD nonrobust glcm JointEntropy | 0.564 | 0.936 | [0.897-0.978] | 0.003 | ** | 0.508 | 0.993 | [0.886-1.114] | 0.909 |  |
| LD nonrobust glcm MCC | 0.527 | 1.019 | [0.976-1.065] | 0.396 |  | 0.654 | 1.102 | [0.975-1.246] | 0.126 |  |
| LD nonrobust glcm MaximumProbability | 0.562 | 1.042 | [0.997-1.088] | 0.067 |  | 0.543 | 1.045 | [0.935-1.169] | 0.440 |  |
| LD nonrobust glcm SumAverage | 0.494 | 0.984 | [0.941-1.027] | 0.457 |  | 0.494 | 1.008 | [0.895-1.134] | 0.902 |  |
| LD nonrobust glcm SumEntropy | 0.449 | 0.944 | [0.904-0.986] | 0.009 | ** | 0.483 | 0.988 | [0.884-1.103] | 0.826 |  |
| LD nonrobust glcm SumSquares | 0.507 | 0.974 | [0.933-1.018] | 0.242 |  | 0.461 | 0.972 | [0.872-1.084] | 0.613 |  |
| LD nonrobust gldm DependenceEntropy | 0.481 | 0.983 | [0.941-1.027] | 0.445 |  | 0.570 | 1.062 | [0.945-1.192] | 0.318 |  |
| LD nonrobust gldm DependenceNonUniformity | 0.512 | 1.029 | [0.985-1.075] | 0.200 |  | 0.579 | 1.075 | [0.963-1.2] | 0.204 |  |
| LD nonrobust gldm DependenceVariance | 0.545 | 1.048 | [1.003-1.095] | 0.035 | * | 0.557 | 1.059 | [0.949-1.183] | 0.311 |  |
| LD nonrobust gldm GrayLevelVariance | 0.510 | 0.974 | [0.933-1.018] | 0.242 |  | 0.443 | 0.966 | [0.865-1.078] | 0.538 |  |
| LD nonrobust gldm HighGrayLevelEmphasis | 0.507 | 0.984 | [0.942-1.028] | 0.481 |  | 0.490 | 1.013 | [0.899-1.142] | 0.831 |  |
| LD nonrobust gldm LargeDependenceEmphasis | 0.548 | 1.052 | [1.008-1.099] | 0.021 | * | 0.565 | 1.049 | [0.937-1.173] | 0.411 |  |
| LD nonrobust gldm LargeDependenceHighGrayLevelEmphasis | 0.514 | 1.039 | [0.994-1.085] | 0.088 |  | 0.554 | 1.054 | [0.945-1.175] | 0.351 |  |
| LD nonrobust gldm LargeDependenceLowGrayLevelEmphasis | 0.582 | 1.06 | [1.015-1.107] | 0.009 | ** | 0.568 | 1.019 | [0.938-1.107] | 0.660 |  |
| LD nonrobust gldm LowGrayLevelEmphasis | 0.520 | 1.063 | [1.018-1.11] | 0.006 | ** | 0.588 | 0.945 | [0.843-1.06] | 0.339 |  |
| LD nonrobust gldm SmallDependenceHighGrayLevelEmphasis | 0.543 | 0.949 | [0.908-0.991] | 0.018 | * | 0.588 | 0.954 | [0.842-1.081] | 0.464 |  |
| LD nonrobust gldm SmallDependenceLowGrayLevelEmphasis | 0.512 | 1 | [0.957-1.045] | 0.996 |  | 0.614 | 0.934 | [0.838-1.042] | 0.227 |  |
| LD nonrobust glrlm GrayLevelNonUniformityNormalized | 0.548 | 1.055 | [1.01-1.102] | 0.016 | * | 0.472 | 1.007 | [0.896-1.131] | 0.908 |  |
| LD nonrobust glrlm GrayLevelVariance | 0.507 | 0.997 | [0.954-1.041] | 0.880 |  | 0.437 | 0.953 | [0.853-1.064] | 0.396 |  |
| LD nonrobust glrlm HighGrayLevelRunEmphasis | 0.509 | 0.981 | [0.939-1.025] | 0.394 |  | 0.506 | 1.009 | [0.895-1.137] | 0.885 |  |
| LD nonrobust glrlm LongRunEmphasis | 0.548 | 1.047 | [1.003-1.094] | 0.038 | * | 0.570 | 1.051 | [0.933-1.183] | 0.419 |  |
| LD nonrobust glrlm LongRunHighGrayLevelEmphasis | 0.495 | 1.026 | [0.982-1.072] | 0.251 |  | 0.537 | 1.044 | [0.935-1.166] | 0.449 |  |
| LD nonrobust glrlm LongRunLowGrayLevelEmphasis | 0.535 | 1.063 | [1.017-1.11] | 0.006 | ** | 0.574 | 0.952 | [0.86-1.053] | 0.346 |  |
| LD nonrobust glrlm LowGrayLevelRunEmphasis | 0.523 | 1.062 | [1.017-1.109] | 0.007 | ** | 0.588 | 0.94 | [0.833-1.061] | 0.319 |  |
| LD nonrobust glrlm RunEntropy | 0.470 | 0.949 | [0.909-0.991] | 0.019 | * | 0.550 | 1.017 | [0.904-1.144] | 0.783 |  |
| LD nonrobust glrlm RunLengthNonUniformity | 0.520 | 1.044 | [1-1.091] | 0.051 |  | 0.583 | 1.061 | [0.947-1.187] | 0.312 |  |
| LD nonrobust glrlm RunVariance | 0.549 | 1.044 | [1-1.091] | 0.052 |  | 0.572 | 1.058 | [0.941-1.19] | 0.349 |  |
| LD nonrobust glrlm ShortRunHighGrayLevelEmphasis | 0.521 | 0.98 | [0.938-1.024] | 0.363 |  | 0.514 | 1.001 | [0.89-1.126] | 0.987 |  |
| LD nonrobust glrlm ShortRunLowGrayLevelEmphasis | 0.521 | 1.058 | [1.013-1.105] | 0.011 | * | 0.585 | 0.94 | [0.833-1.059] | 0.314 |  |
| LD nonrobust glszm GrayLevelNonUniformityNormalized | 0.484 | 1.048 | [1.003-1.094] | 0.036 | * | 0.457 | 1.017 | [0.897-1.153] | 0.791 |  |
| LD nonrobust glszm GrayLevelVariance | 0.502 | 0.996 | [0.953-1.04] | 0.848 |  | 0.576 | 0.951 | [0.851-1.062] | 0.375 |  |
| LD nonrobust glszm HighGrayLevelZoneEmphasis | 0.528 | 0.966 | [0.924-1.009] | 0.116 |  | 0.492 | 1.011 | [0.898-1.139] | 0.853 |  |
| LD nonrobust glszm LargeAreaEmphasis | 0.547 | 1.018 | [0.975-1.064] | 0.415 |  | 0.561 | 1.013 | [0.903-1.135] | 0.832 |  |
| LD nonrobust glszm LargeAreaHighGrayLevelEmphasis | 0.534 | 1.043 | [0.998-1.089] | 0.060 |  | 0.554 | 1.031 | [0.869-1.224] | 0.725 |  |
| LD nonrobust glszm LargeAreaLowGrayLevelEmphasis | 0.570 | 1.014 | [0.971-1.06] | 0.525 |  | 0.570 | 1.006 | [0.859-1.178] | 0.938 |  |
| LD nonrobust glszm LowGrayLevelZoneEmphasis | 0.518 | 1.047 | [1.002-1.093] | 0.040 | * | 0.621 | 0.926 | [0.824-1.04] | 0.199 |  |
| LD nonrobust glszm SizeZoneNonUniformity | 0.506 | 0.999 | [0.956-1.043] | 0.947 |  | 0.576 | 1.057 | [0.936-1.194] | 0.378 |  |
| LD nonrobust glszm SizeZoneNonUniformityNormalized | 0.511 | 0.98 | [0.938-1.024] | 0.365 |  | 0.627 | 0.932 | [0.83-1.046] | 0.235 |  |
| LD nonrobust glszm SmallAreaEmphasis | 0.512 | 0.971 | [0.929-1.014] | 0.184 |  | 0.625 | 0.935 | [0.818-1.069] | 0.330 |  |
| LD nonrobust glszm SmallAreaHighGrayLevelEmphasis | 0.522 | 0.966 | [0.925-1.009] | 0.121 |  | 0.532 | 0.997 | [0.887-1.121] | 0.962 |  |
| LD nonrobust glszm SmallAreaLowGrayLevelEmphasis | 0.498 | 1.022 | [0.979-1.068] | 0.322 |  | 0.641 | 0.923 | [0.828-1.029] | 0.154 |  |
| LD nonrobust glszm ZoneEntropy | 0.512 | 0.987 | [0.945-1.031] | 0.569 |  | 0.601 | 1.066 | [0.942-1.207] | 0.316 |  |
| LD nonrobust glszm ZoneVariance | 0.547 | 1.018 | [0.975-1.064] | 0.417 |  | 0.554 | 1.012 | [0.903-1.135] | 0.833 |  |
| LD nonrobust ngtdm Busyness | 0.533 | 1.051 | [1.006-1.097] | 0.027 | * | 0.579 | 1.052 | [0.932-1.187] | 0.418 |  |
| LD nonrobust ngtdm Coarseness | 0.528 | 0.98 | [0.938-1.024] | 0.372 |  | 0.543 | 0.968 | [0.858-1.092] | 0.598 |  |
| LD nonrobust ngtdm Complexity | 0.531 | 0.968 | [0.927-1.012] | 0.150 |  | 0.603 | 0.93 | [0.825-1.047] | 0.236 |  |
| LD nonrobust ngtdm Contrast | 0.475 | 0.976 | [0.935-1.02] | 0.286 |  | 0.596 | 0.939 | [0.839-1.051] | 0.279 |  |

Supplementary Table S5a - Univariate analysis of radiomic features to predict regional or distant recurrence.

|  | **Center A** | | | | | **Center B** | | | | |
| --- | --- | --- | --- | --- | --- | --- | --- | --- | --- | --- |
| **feature** | **AUC** | **OR** | **CI** | **p-val** | **sign** | **AUC** | **OR** | **CI** | **p-val** | **sign** |
| Robust shape LeastAxisLength | 0.598 | 1.077 | [1.034-1.122] | 0.000 | *** | 0.571 | 1.039 | [0.929-1.161] | 0.504 |  |
| Robust shape Maximum2DDiameterSlice | 0.587 | 1.068 | [1.025-1.113] | 0.002 | ** | 0.555 | 1.057 | [0.946-1.181] | 0.330 |  |
| Robust shape MinorAxisLength | 0.598 | 1.079 | [1.036-1.124] | 0.000 | *** | 0.550 | 1.026 | [0.921-1.144] | 0.639 |  |
| Robust shape SurfaceArea | 0.589 | 1.071 | [1.028-1.116] | 0.001 | ** | 0.574 | 1.046 | [0.936-1.17] | 0.430 |  |
| Robust shape SurfaceVolumeRatio | 0.607 | 0.918 | [0.882-0.957] | 0.000 | *** | 0.564 | 0.960 | [0.859-1.074] | 0.480 |  |
| Robust glcm DifferenceAverage | 0.603 | 0.917 | [0.88-0.955] | 0.000 | *** | 0.590 | 0.938 | [0.836-1.053] | 0.284 |  |
| Robust glcm Id | 0.617 | 1.111 | [1.067-1.157] | 0.000 | *** | 0.567 | 1.042 | [0.929-1.169] | 0.489 |  |
| Robust glcm Idm | 0.618 | 1.112 | [1.067-1.158] | 0.000 | *** | 0.571 | 1.036 | [0.923-1.163] | 0.550 |  |
| Robust glcm Idn | 0.606 | 1.093 | [1.049-1.138] | 0.000 | *** | 0.595 | 1.064 | [0.948-1.195] | 0.297 |  |
| Robust glcm InverseVariance | 0.600 | 1.087 | [1.044-1.133] | 0.000 | *** | 0.557 | 1.035 | [0.926-1.158] | 0.544 |  |
| Robust gldm DependenceNonUniformityNormalized | 0.617 | 0.913 | [0.876-0.951] | 0.000 | *** | 0.569 | 0.954 | [0.85-1.07] | 0.422 |  |
| Robust gldm GrayLevelNonUniformity | 0.610 | 1.082 | [1.039-1.128] | 0.000 | *** | 0.548 | 1.022 | [0.876-1.193] | 0.782 |  |
| Robust gldm SmallDependenceEmphasis | 0.615 | 0.909 | [0.873-0.947] | 0.000 | *** | 0.600 | 0.943 | [0.841-1.057] | 0.317 |  |
| Robust glrlm GrayLevelNonUniformity | 0.606 | 1.08 | [1.037-1.126] | 0.000 | *** | 0.543 | 1.013 | [0.902-1.137] | 0.830 |  |
| Robust glrlm RunLengthNonUniformityNormalized | 0.618 | 0.901 | [0.865-0.938] | 0.000 | *** | 0.560 | 0.969 | [0.862-1.09] | 0.604 |  |
| Robust glrlm RunPercentage | 0.626 | 0.893 | [0.858-0.93] | 0.000 | *** | 0.562 | 0.967 | [0.859-1.088] | 0.580 |  |
| Robust glrlm ShortRunEmphasis | 0.618 | 0.899 | [0.863-0.936] | 0.000 | *** | 0.560 | 0.971 | [0.864-1.091] | 0.621 |  |
| Robust glszm GrayLevelNonUniformity | 0.575 | 1.06 | [1.017-1.105] | 0.006 | ** | 0.564 | 1.040 | [0.929-1.165] | 0.496 |  |
| Robust glszm ZonePercentage | 0.615 | 0.907 | [0.871-0.945] | 0.000 | *** | 0.588 | 0.953 | [0.851-1.067] | 0.411 |  |
| HD nonrobust shape Elongation | 0.470 | 1.033 | [0.991-1.077] | 0.126 |  | 0.583 | 0.952 | [0.854-1.062] | 0.382 |  |
| HD nonrobust shape Flatness | 0.512 | 1.018 | [0.977-1.061] | 0.399 |  | 0.524 | 0.977 | [0.874-1.091] | 0.676 |  |
| HD nonrobust shape MajorAxisLength | 0.573 | 1.061 | [1.018-1.105] | 0.005 | ** | 0.626 | 1.057 | [0.946-1.18] | 0.332 |  |
| HD nonrobust shape Maximum2DDiameterColumn | 0.582 | 1.066 | [1.023-1.111] | 0.002 | ** | 0.567 | 1.044 | [0.934-1.167] | 0.452 |  |
| HD nonrobust shape Maximum2DDiameterRow | 0.576 | 1.059 | [1.016-1.104] | 0.006 | ** | 0.577 | 1.041 | [0.928-1.168] | 0.494 |  |
| HD nonrobust shape Maximum3DDiameter | 0.572 | 1.057 | [1.015-1.102] | 0.008 | ** | 0.593 | 1.044 | [0.933-1.17] | 0.455 |  |
| HD nonrobust shape MeshVolume | 0.597 | 1.079 | [1.035-1.124] | 0.000 | *** | 0.564 | 1.045 | [0.935-1.168] | 0.442 |  |
| HD nonrobust shape Sphericity | 0.518 | 1.014 | [0.973-1.057] | 0.518 |  | 0.476 | 0.97 | [0.86-1.093] | 0.619 |  |
| HD nonrobust shape VoxelVolume | 0.597 | 1.079 | [1.035-1.124] | 0.000 | *** | 0.564 | 1.045 | [0.935-1.168] | 0.442 |  |
| HD nonrobust firstorder 10Percentile | 0.590 | 1.063 | [1.02-1.108] | 0.004 | ** | 0.569 | 0.953 | [0.848-1.071] | 0.424 |  |
| HD nonrobust firstorder 90Percentile | 0.499 | 1.02 | [0.978-1.063] | 0.351 |  | 0.462 | 0.976 | [0.874-1.09] | 0.666 |  |
| HD nonrobust firstorder Energy | 0.540 | 1.029 | [0.987-1.073] | 0.177 |  | 0.612 | 1.085 | [0.967-1.217] | 0.170 |  |
| HD nonrobust firstorder Entropy | 0.612 | 0.906 | [0.87-0.944] | 0.000 | *** | 0.524 | 1.024 | [0.912-1.149] | 0.694 |  |
| HD nonrobust firstorder InterquartileRange | 0.574 | 0.94 | [0.902-0.979] | 0.003 | ** | 0.536 | 1.033 | [0.92-1.161] | 0.583 |  |
| HD nonrobust firstorder Kurtosis | 0.589 | 1.1 | [1.056-1.145] | 0.000 | *** | 0.474 | 0.949 | [0.839-1.073] | 0.409 |  |
| HD nonrobust firstorder Maximum | 0.574 | 1.088 | [1.045-1.134] | 0.000 | *** | 0.593 | 0.935 | [0.801-1.091] | 0.400 |  |
| HD nonrobust firstorder MeanAbsoluteDeviation | 0.556 | 0.949 | [0.91-0.989] | 0.013 | * | 0.548 | 1.027 | [0.913-1.156] | 0.659 |  |
| HD nonrobust firstorder Mean | 0.598 | 1.065 | [1.022-1.11] | 0.003 | ** | 0.457 | 0.969 | [0.861-1.092] | 0.611 |  |
| HD nonrobust firstorder Median | 0.593 | 1.066 | [1.023-1.111] | 0.003 | ** | 0.536 | 0.978 | [0.868-1.103] | 0.720 |  |
| HD nonrobust firstorder Minimum | 0.481 | 1.026 | [0.984-1.07] | 0.223 |  | 0.567 | 0.979 | [0.871-1.1] | 0.720 |  |
| HD nonrobust firstorder Range | 0.536 | 1.078 | [1.035-1.123] | 0.000 | *** | 0.510 | 0.977 | [0.856-1.115] | 0.729 |  |
| HD nonrobust firstorder RobustMeanAbsoluteDeviation | 0.566 | 0.944 | [0.905-0.983] | 0.006 | ** | 0.457 | 1.034 | [0.919-1.164] | 0.579 |  |
| HD nonrobust firstorder RootMeanSquared | 0.588 | 0.935 | [0.898-0.975] | 0.002 | ** | 0.557 | 1.041 | [0.929-1.167] | 0.489 |  |
| HD nonrobust firstorder Skewness | 0.555 | 1.009 | [0.968-1.052] | 0.683 |  | 0.531 | 1.028 | [0.914-1.156] | 0.644 |  |
| HD nonrobust firstorder TotalEnergy | 0.540 | 1.029 | [0.987-1.073] | 0.177 |  | 0.612 | 1.085 | [0.967-1.217] | 0.170 |  |
| HD nonrobust firstorder Uniformity | 0.618 | 1.108 | [1.064-1.154] | 0.000 | *** | 0.495 | 0.975 | [0.869-1.095] | 0.675 |  |
| HD nonrobust firstorder Variance | 0.537 | 0.968 | [0.929-1.009] | 0.131 |  | 0.538 | 1.028 | [0.909-1.163] | 0.661 |  |
| HD nonrobust glcm Autocorrelation | 0.521 | 0.997 | [0.956-1.039] | 0.870 |  | 0.483 | 0.981 | [0.878-1.097] | 0.743 |  |
| HD nonrobust glcm ClusterProminence | 0.556 | 0.963 | [0.924-1.004] | 0.074 |  | 0.583 | 1.064 | [0.951-1.191] | 0.282 |  |
| HD nonrobust glcm ClusterShade | 0.515 | 0.988 | [0.948-1.03] | 0.583 |  | 0.500 | 0.971 | [0.871-1.082] | 0.596 |  |
| HD nonrobust glcm ClusterTendency | 0.578 | 0.939 | [0.901-0.978] | 0.003 | ** | 0.574 | 1.067 | [0.947-1.201] | 0.294 |  |
| HD nonrobust glcm Contrast | 0.590 | 0.929 | [0.892-0.968] | 0.001 | *** | 0.588 | 0.934 | [0.835-1.046] | 0.243 |  |
| HD nonrobust glcm Correlation | 0.510 | 1.005 | [0.964-1.048] | 0.805 |  | 0.669 | 1.1 | [0.988-1.225] | 0.089 |  |
| HD nonrobust glcm DifferenceEntropy | 0.601 | 0.909 | [0.873-0.947] | 0.000 | *** | 0.576 | 0.95 | [0.846-1.065] | 0.381 |  |
| HD nonrobust glcm DifferenceVariance | 0.562 | 0.943 | [0.905-0.983] | 0.006 | ** | 0.593 | 0.94 | [0.838-1.054] | 0.293 |  |
| HD nonrobust glcm Idmn | 0.593 | 1.078 | [1.035-1.123] | 0.000 | *** | 0.588 | 1.07 | [0.955-1.2] | 0.246 |  |
| HD nonrobust glcm Imc1 | 0.533 | 1.032 | [0.99-1.076] | 0.135 |  | 0.598 | 0.966 | [0.864-1.079] | 0.540 |  |
| HD nonrobust glcm Imc2 | 0.602 | 0.924 | [0.887-0.963] | 0.000 | *** | 0.619 | 1.063 | [0.943-1.199] | 0.322 |  |
| HD nonrobust glcm JointAverage | 0.523 | 1.011 | [0.969-1.053] | 0.622 |  | 0.474 | 0.975 | [0.873-1.09] | 0.663 |  |
| HD nonrobust glcm JointEnergy | 0.626 | 1.111 | [1.067-1.157] | 0.000 | *** | 0.457 | 0.986 | [0.876-1.11] | 0.817 |  |
| HD nonrobust glcm JointEntropy | 0.604 | 0.911 | [0.874-0.948] | 0.000 | *** | 0.505 | 1.019 | [0.908-1.145] | 0.750 |  |
| HD nonrobust glcm MCC | 0.503 | 0.998 | [0.958-1.041] | 0.939 |  | 0.664 | 1.101 | [0.985-1.231] | 0.098 |  |
| HD nonrobust glcm MaximumProbability | 0.622 | 1.119 | [1.075-1.165] | 0.000 | *** | 0.524 | 1.008 | [0.897-1.131] | 0.898 |  |
| HD nonrobust glcm SumAverage | 0.523 | 1.011 | [0.969-1.053] | 0.622 |  | 0.474 | 0.975 | [0.873-1.09] | 0.663 |  |
| HD nonrobust glcm SumEntropy | 0.609 | 0.908 | [0.872-0.945] | 0.000 | *** | 0.488 | 1.036 | [0.922-1.164] | 0.551 |  |
| HD nonrobust glcm SumSquares | 0.582 | 0.935 | [0.897-0.974] | 0.001 | ** | 0.564 | 1.045 | [0.929-1.175] | 0.466 |  |
| HD nonrobust gldm DependenceEntropy | 0.528 | 1.012 | [0.971-1.055] | 0.575 |  | 0.655 | 1.084 | [0.971-1.21] | 0.156 |  |
| HD nonrobust gldm DependenceNonUniformity | 0.553 | 1.044 | [1.002-1.088] | 0.042 | * | 0.586 | 1.041 | [0.934-1.159] | 0.473 |  |
| HD nonrobust gldm DependenceVariance | 0.629 | 1.122 | [1.077-1.168] | 0.000 | *** | 0.536 | 1.036 | [0.924-1.16] | 0.548 |  |
| HD nonrobust gldm GrayLevelVariance | 0.573 | 0.934 | [0.896-0.973] | 0.001 | ** | 0.552 | 1.035 | [0.925-1.159] | 0.549 |  |
| HD nonrobust gldm HighGrayLevelEmphasis | 0.527 | 1.004 | [0.963-1.047] | 0.845 |  | 0.493 | 0.984 | [0.88-1.1] | 0.775 |  |
| HD nonrobust gldm LargeDependenceEmphasis | 0.629 | 1.125 | [1.081-1.171] | 0.000 | *** | 0.543 | 1.032 | [0.916-1.164] | 0.605 |  |
| HD nonrobust gldm LargeDependenceHighGrayLevelEmphasis | 0.575 | 1.068 | [1.025-1.113] | 0.002 | ** | 0.531 | 1.027 | [0.914-1.153] | 0.661 |  |
| HD nonrobust gldm LargeDependenceLowGrayLevelEmphasis | 0.556 | 1.053 | [1.01-1.097] | 0.015 | * | 0.562 | 1.578 | [0.693-3.593] | 0.283 |  |
| HD nonrobust gldm LowGrayLevelEmphasis | 0.555 | 1.049 | [1.006-1.093] | 0.025 | * | 0.498 | 0.968 | [0.844-1.111] | 0.649 |  |
| HD nonrobust gldm SmallDependenceHighGrayLevelEmphasis | 0.594 | 0.926 | [0.889-0.965] | 0.000 | *** | 0.612 | 0.941 | [0.844-1.051] | 0.287 |  |
| HD nonrobust gldm SmallDependenceLowGrayLevelEmphasis | 0.565 | 0.948 | [0.909-0.988] | 0.011 | * | 0.457 | 0.966 | [0.865-1.078] | 0.534 |  |
| HD nonrobust glrlm GrayLevelNonUniformityNormalized | 0.604 | 1.099 | [1.055-1.145] | 0.000 | *** | 0.502 | 0.967 | [0.861-1.085] | 0.570 |  |
| HD nonrobust glrlm GrayLevelVariance | 0.570 | 0.934 | [0.896-0.973] | 0.001 | ** | 0.545 | 1.033 | [0.923-1.155] | 0.574 |  |
| HD nonrobust glrlm HighGrayLevelRunEmphasis | 0.524 | 1.003 | [0.962-1.045] | 0.904 |  | 0.483 | 0.979 | [0.876-1.094] | 0.704 |  |
| HD nonrobust glrlm LongRunEmphasis | 0.629 | 1.124 | [1.079-1.17] | 0.000 | *** | 0.564 | 1.034 | [0.912-1.172] | 0.604 |  |
| HD nonrobust glrlm LongRunHighGrayLevelEmphasis | 0.561 | 1.062 | [1.019-1.107] | 0.004 | ** | 0.507 | 1.006 | [0.896-1.129] | 0.920 |  |
| HD nonrobust glrlm LongRunLowGrayLevelEmphasis | 0.534 | 1.046 | [1.003-1.09] | 0.035 | * | 0.531 | 0.937 | [0.622-1.412] | 0.758 |  |
| HD nonrobust glrlm LowGrayLevelRunEmphasis | 0.549 | 1.033 | [0.991-1.077] | 0.126 |  | 0.505 | 0.973 | [0.863-1.097] | 0.658 |  |
| HD nonrobust glrlm RunEntropy | 0.559 | 0.933 | [0.895-0.972] | 0.001 | *** | 0.605 | 1.075 | [0.957-1.208] | 0.227 |  |
| HD nonrobust glrlm RunLengthNonUniformity | 0.583 | 1.065 | [1.022-1.11] | 0.003 | ** | 0.571 | 1.044 | [0.936-1.164] | 0.443 |  |
| HD nonrobust glrlm RunVariance | 0.631 | 1.123 | [1.078-1.169] | 0.000 | *** | 0.560 | 1.041 | [0.907-1.195] | 0.574 |  |
| HD nonrobust glrlm ShortRunHighGrayLevelEmphasis | 0.507 | 0.985 | [0.945-1.027] | 0.489 |  | 0.467 | 0.971 | [0.87-1.084] | 0.604 |  |
| HD nonrobust glrlm ShortRunLowGrayLevelEmphasis | 0.549 | 1.003 | [0.962-1.045] | 0.892 |  | 0.500 | 0.972 | [0.864-1.094] | 0.641 |  |
| HD nonrobust glszm GrayLevelNonUniformityNormalized | 0.556 | 1.083 | [1.039-1.128] | 0.000 | *** | 0.550 | 0.995 | [0.89-1.111] | 0.924 |  |
| HD nonrobust glszm GrayLevelVariance | 0.564 | 0.948 | [0.91-0.988] | 0.012 | * | 0.557 | 0.966 | [0.865-1.079] | 0.543 |  |
| HD nonrobust glszm HighGrayLevelZoneEmphasis | 0.504 | 0.987 | [0.947-1.029] | 0.540 |  | 0.529 | 0.962 | [0.862-1.073] | 0.492 |  |
| HD nonrobust glszm LargeAreaEmphasis | 0.620 | 1.067 | [1.023-1.111] | 0.002 | ** | 0.564 | 1.012 | [0.906-1.13] | 0.837 |  |
| HD nonrobust glszm LargeAreaHighGrayLevelEmphasis | 0.594 | 1.048 | [1.005-1.092] | 0.027 | * | 0.548 | 1.01 | [0.908-1.123] | 0.857 |  |
| HD nonrobust glszm LargeAreaLowGrayLevelEmphasis | 0.604 | 1.045 | [1.003-1.089] | 0.037 | * | 0.574 | 1.239 | [0.602-2.548] | 0.563 |  |
| HD nonrobust glszm LowGrayLevelZoneEmphasis | 0.522 | 1.026 | [0.985-1.07] | 0.222 |  | 0.490 | 0.984 | [0.874-1.107] | 0.786 |  |
| HD nonrobust glszm SizeZoneNonUniformity | 0.556 | 1.033 | [0.991-1.077] | 0.126 |  | 0.529 | 1.023 | [0.914-1.144] | 0.698 |  |
| HD nonrobust glszm SizeZoneNonUniformityNormalized | 0.580 | 0.932 | [0.894-0.971] | 0.001 | *** | 0.619 | 0.921 | [0.824-1.029] | 0.152 |  |
| HD nonrobust glszm SmallAreaEmphasis | 0.580 | 0.93 | [0.893-0.969] | 0.001 | *** | 0.621 | 0.925 | [0.832-1.028] | 0.153 |  |
| HD nonrobust glszm SmallAreaHighGrayLevelEmphasis | 0.507 | 0.988 | [0.948-1.03] | 0.574 |  | 0.421 | 0.949 | [0.854-1.054] | 0.329 |  |
| HD nonrobust glszm SmallAreaLowGrayLevelEmphasis | 0.480 | 1.012 | [0.971-1.055] | 0.573 |  | 0.493 | 0.979 | [0.875-1.097] | 0.722 |  |
| HD nonrobust glszm ZoneEntropy | 0.525 | 1.019 | [0.978-1.062] | 0.376 |  | 0.614 | 1.075 | [0.961-1.203] | 0.213 |  |
| HD nonrobust glszm ZoneVariance | 0.619 | 1.066 | [1.023-1.111] | 0.002 | ** | 0.550 | 1.012 | [0.906-1.13] | 0.839 |  |
| HD nonrobust ngtdm Busyness | 0.586 | 1.086 | [1.042-1.131] | 0.000 | *** | 0.552 | 1.027 | [0.909-1.159] | 0.675 |  |
| HD nonrobust ngtdm Coarseness | 0.602 | 0.948 | [0.909-0.988] | 0.011 | * | 0.531 | 0.994 | [0.891-1.11] | 0.918 |  |
| HD nonrobust ngtdm Complexity | 0.594 | 0.921 | [0.884-0.959] | 0.000 | *** | 0.669 | 0.907 | [0.815-1.01] | 0.081 |  |
| HD nonrobust ngtdm Contrast | 0.594 | 0.929 | [0.892-0.968] | 0.001 | *** | 0.467 | 0.954 | [0.851-1.07] | 0.425 |  |
| LD nonrobust shape Elongation | 0.478 | 1.021 | [0.979-1.064] | 0.333 |  | 0.576 | 0.978 | [0.878-1.09] | 0.689 |  |
| LD nonrobust shape Flatness | 0.516 | 0.989 | [0.948-1.03] | 0.587 |  | 0.648 | 0.958 | [0.858-1.07] | 0.451 |  |
| LD nonrobust shape MajorAxisLength | 0.567 | 1.066 | [1.023-1.111] | 0.002 | ** | 0.443 | 1.029 | [0.92-1.152] | 0.615 |  |
| LD nonrobust shape Maximum2DDiameterColumn | 0.570 | 1.071 | [1.028-1.116] | 0.001 | ** | 0.467 | 1.026 | [0.919-1.146] | 0.646 |  |
| LD nonrobust shape Maximum2DDiameterRow | 0.568 | 1.067 | [1.024-1.112] | 0.002 | ** | 0.479 | 1.023 | [0.914-1.144] | 0.695 |  |
| LD nonrobust shape Maximum3DDiameter | 0.566 | 1.067 | [1.024-1.112] | 0.002 | ** | 0.444 | 1.045 | [0.936-1.168] | 0.437 |  |
| LD nonrobust shape MeshVolume | 0.568 | 1.083 | [1.039-1.128] | 0.000 | *** | 0.533 | 1.019 | [0.912-1.139] | 0.744 |  |
| LD nonrobust shape Sphericity | 0.510 | 0.994 | [0.954-1.036] | 0.785 |  | 0.450 | 0.976 | [0.878-1.086] | 0.662 |  |
| LD nonrobust shape VoxelVolume | 0.568 | 1.083 | [1.039-1.128] | 0.000 | *** | 0.533 | 1.019 | [0.912-1.139] | 0.744 |  |
| LD nonrobust firstorder 10Percentile | 0.563 | 1.044 | [1.001-1.088] | 0.043 | * | 0.531 | 1.013 | [0.908-1.13] | 0.822 |  |
| LD nonrobust firstorder 90Percentile | 0.574 | 1.015 | [0.974-1.058] | 0.474 |  | 0.510 | 1.005 | [0.868-1.162] | 0.951 |  |
| LD nonrobust firstorder Energy | 0.537 | 1.059 | [1.016-1.103] | 0.007 | ** | 0.574 | 1.029 | [0.921-1.149] | 0.615 |  |
| LD nonrobust firstorder Entropy | 0.594 | 0.917 | [0.88-0.955] | 0.000 | *** | 0.486 | 0.985 | [0.883-1.099] | 0.789 |  |
| LD nonrobust firstorder InterquartileRange | 0.543 | 0.967 | [0.928-1.008] | 0.114 |  | 0.552 | 0.978 | [0.88-1.088] | 0.687 |  |
| LD nonrobust firstorder Kurtosis | 0.550 | 1.064 | [1.021-1.109] | 0.003 | ** | 0.471 | 1.02 | [0.905-1.15] | 0.744 |  |
| LD nonrobust firstorder Maximum | 0.555 | 1.055 | [1.012-1.099] | 0.012 | * | 0.557 | 1.041 | [0.907-1.196] | 0.570 |  |
| LD nonrobust firstorder MeanAbsoluteDeviation | 0.527 | 0.978 | [0.938-1.02] | 0.298 |  | 0.533 | 0.984 | [0.884-1.096] | 0.768 |  |
| LD nonrobust firstorder Mean | 0.573 | 1.041 | [0.999-1.085] | 0.057 |  | 0.510 | 1.002 | [0.888-1.131] | 0.973 |  |
| LD nonrobust firstorder Median | 0.571 | 1.043 | [1.001-1.087] | 0.048 | * | 0.488 | 0.998 | [0.886-1.124] | 0.975 |  |
| LD nonrobust firstorder Minimum | 0.499 | 1.01 | [0.969-1.053] | 0.637 |  | 0.571 | 0.965 | [0.863-1.078] | 0.531 |  |
| LD nonrobust firstorder Range | 0.534 | 1.048 | [1.006-1.093] | 0.026 | * | 0.595 | 1.056 | [0.923-1.207] | 0.433 |  |
| LD nonrobust firstorder RobustMeanAbsoluteDeviation | 0.536 | 0.972 | [0.933-1.014] | 0.188 |  | 0.552 | 0.979 | [0.88-1.09] | 0.700 |  |
| LD nonrobust firstorder RootMeanSquared | 0.567 | 0.964 | [0.925-1.005] | 0.085 |  | 0.524 | 1.001 | [0.89-1.126] | 0.987 |  |
| LD nonrobust firstorder Skewness | 0.551 | 0.98 | [0.94-1.022] | 0.344 |  | 0.500 | 0.988 | [0.885-1.104] | 0.837 |  |
| LD nonrobust firstorder TotalEnergy | 0.537 | 1.059 | [1.016-1.103] | 0.007 | ** | 0.574 | 1.029 | [0.921-1.149] | 0.615 |  |
| LD nonrobust firstorder Uniformity | 0.606 | 1.075 | [1.032-1.12] | 0.001 | *** | 0.512 | 1.02 | [0.914-1.138] | 0.728 |  |
| LD nonrobust firstorder Variance | 0.486 | 0.993 | [0.953-1.036] | 0.757 |  | 0.514 | 0.987 | [0.885-1.1] | 0.811 |  |
| LD nonrobust glcm Autocorrelation | 0.537 | 1.012 | [0.971-1.055] | 0.573 |  | 0.505 | 0.996 | [0.886-1.119] | 0.948 |  |
| LD nonrobust glcm ClusterProminence | 0.514 | 0.994 | [0.954-1.037] | 0.789 |  | 0.586 | 0.95 | [0.849-1.063] | 0.377 |  |
| LD nonrobust glcm ClusterShade | 0.536 | 0.978 | [0.938-1.019] | 0.295 |  | 0.579 | 1.043 | [0.932-1.167] | 0.465 |  |
| LD nonrobust glcm ClusterTendency | 0.532 | 0.96 | [0.921-1] | 0.053 |  | 0.455 | 0.972 | [0.873-1.082] | 0.600 |  |
| LD nonrobust glcm Contrast | 0.566 | 0.945 | [0.907-0.985] | 0.008 | ** | 0.579 | 0.952 | [0.857-1.058] | 0.366 |  |
| LD nonrobust glcm Correlation | 0.529 | 1.015 | [0.974-1.058] | 0.475 |  | 0.412 | 1.058 | [0.946-1.185] | 0.328 |  |
| LD nonrobust glcm DifferenceEntropy | 0.574 | 0.939 | [0.901-0.978] | 0.003 | ** | 0.581 | 0.954 | [0.858-1.061] | 0.386 |  |
| LD nonrobust glcm DifferenceVariance | 0.538 | 0.973 | [0.934-1.014] | 0.198 |  | 0.602 | 0.931 | [0.826-1.048] | 0.241 |  |
| LD nonrobust glcm Idmn | 0.567 | 1.048 | [1.005-1.092] | 0.027 | * | 0.576 | 1.054 | [0.947-1.172] | 0.341 |  |
| LD nonrobust glcm Imc1 | 0.500 | 0.988 | [0.947-1.03] | 0.558 |  | 0.517 | 1.018 | [0.913-1.135] | 0.752 |  |
| LD nonrobust glcm Imc2 | 0.567 | 0.953 | [0.914-0.993] | 0.022 | * | 0.498 | 0.991 | [0.891-1.102] | 0.869 |  |
| LD nonrobust glcm JointAverage | 0.539 | 1.015 | [0.974-1.058] | 0.480 |  | 0.512 | 0.998 | [0.891-1.118] | 0.974 |  |
| LD nonrobust glcm JointEnergy | 0.619 | 1.06 | [1.017-1.105] | 0.006 | ** | 0.507 | 1.036 | [0.924-1.162] | 0.546 |  |
| LD nonrobust glcm JointEntropy | 0.609 | 0.914 | [0.877-0.952] | 0.000 | *** | 0.505 | 0.993 | [0.89-1.109] | 0.906 |  |
| LD nonrobust glcm MCC | 0.560 | 1.04 | [0.998-1.084] | 0.061 |  | 0.614 | 1.072 | [0.951-1.208] | 0.263 |  |
| LD nonrobust glcm MaximumProbability | 0.608 | 1.068 | [1.025-1.113] | 0.002 | ** | 0.502 | 1.027 | [0.921-1.145] | 0.632 |  |
| LD nonrobust glcm SumAverage | 0.539 | 1.015 | [0.974-1.058] | 0.480 |  | 0.512 | 0.998 | [0.891-1.118] | 0.974 |  |
| LD nonrobust glcm SumEntropy | 0.602 | 0.918 | [0.881-0.956] | 0.000 | *** | 0.502 | 0.988 | [0.888-1.099] | 0.824 |  |
| LD nonrobust glcm SumSquares | 0.534 | 0.957 | [0.918-0.997] | 0.037 | * | 0.555 | 0.966 | [0.87-1.073] | 0.526 |  |
| LD nonrobust gldm DependenceEntropy | 0.504 | 1.001 | [0.96-1.044] | 0.962 |  | 0.521 | 1.029 | [0.919-1.152] | 0.621 |  |
| LD nonrobust gldm DependenceNonUniformity | 0.546 | 1.054 | [1.011-1.098] | 0.014 | * | 0.529 | 1.024 | [0.919-1.14] | 0.668 |  |
| LD nonrobust gldm DependenceVariance | 0.593 | 1.078 | [1.035-1.123] | 0.000 | *** | 0.510 | 1.031 | [0.926-1.148] | 0.577 |  |
| LD nonrobust gldm GrayLevelVariance | 0.527 | 0.961 | [0.922-1.002] | 0.060 |  | 0.417 | 0.957 | [0.86-1.064] | 0.418 |  |
| LD nonrobust gldm HighGrayLevelEmphasis | 0.539 | 1.016 | [0.975-1.06] | 0.442 |  | 0.510 | 1.002 | [0.893-1.124] | 0.974 |  |
| LD nonrobust gldm LargeDependenceEmphasis | 0.599 | 1.082 | [1.039-1.127] | 0.000 | *** | 0.529 | 1.03 | [0.924-1.148] | 0.601 |  |
| LD nonrobust gldm LargeDependenceHighGrayLevelEmphasis | 0.563 | 1.068 | [1.025-1.113] | 0.002 | ** | 0.517 | 1.034 | [0.93-1.149] | 0.543 |  |
| LD nonrobust gldm LargeDependenceLowGrayLevelEmphasis | 0.567 | 1.068 | [1.025-1.113] | 0.002 | ** | 0.462 | 1.012 | [0.934-1.096] | 0.773 |  |
| LD nonrobust gldm LowGrayLevelEmphasis | 0.526 | 1.05 | [1.007-1.094] | 0.022 | * | 0.564 | 0.956 | [0.855-1.068] | 0.429 |  |
| LD nonrobust gldm SmallDependenceHighGrayLevelEmphasis | 0.557 | 0.941 | [0.903-0.981] | 0.004 | ** | 0.576 | 0.963 | [0.854-1.086] | 0.542 |  |
| LD nonrobust gldm SmallDependenceLowGrayLevelEmphasis | 0.557 | 0.973 | [0.933-1.014] | 0.191 |  | 0.595 | 0.944 | [0.85-1.05] | 0.294 |  |
| LD nonrobust glrlm GrayLevelNonUniformityNormalized | 0.595 | 1.079 | [1.036-1.125] | 0.000 | *** | 0.488 | 1.01 | [0.903-1.13] | 0.863 |  |
| LD nonrobust glrlm GrayLevelVariance | 0.521 | 0.987 | [0.947-1.029] | 0.539 |  | 0.405 | 0.944 | [0.849-1.05] | 0.292 |  |
| LD nonrobust glrlm HighGrayLevelRunEmphasis | 0.536 | 1.013 | [0.971-1.056] | 0.555 |  | 0.524 | 1.001 | [0.892-1.123] | 0.991 |  |
| LD nonrobust glrlm LongRunEmphasis | 0.599 | 1.075 | [1.032-1.12] | 0.001 | *** | 0.536 | 1.034 | [0.922-1.16] | 0.571 |  |
| LD nonrobust glrlm LongRunHighGrayLevelEmphasis | 0.559 | 1.06 | [1.017-1.104] | 0.006 | ** | 0.500 | 1.029 | [0.924-1.145] | 0.607 |  |
| LD nonrobust glrlm LongRunLowGrayLevelEmphasis | 0.507 | 1.063 | [1.02-1.107] | 0.004 | ** | 0.562 | 0.961 | [0.871-1.06] | 0.427 |  |
| LD nonrobust glrlm LowGrayLevelRunEmphasis | 0.521 | 1.044 | [1.002-1.088] | 0.041 | * | 0.567 | 0.951 | [0.846-1.069] | 0.400 |  |
| LD nonrobust glrlm RunEntropy | 0.550 | 0.937 | [0.9-0.977] | 0.002 | ** | 0.507 | 0.998 | [0.891-1.119] | 0.979 |  |
| LD nonrobust glrlm RunLengthNonUniformity | 0.560 | 1.072 | [1.029-1.117] | 0.001 | *** | 0.536 | 1.019 | [0.913-1.137] | 0.737 |  |
| LD nonrobust glrlm RunVariance | 0.599 | 1.071 | [1.027-1.116] | 0.001 | ** | 0.533 | 1.039 | [0.927-1.165] | 0.512 |  |
| LD nonrobust glrlm ShortRunHighGrayLevelEmphasis | 0.519 | 1.01 | [0.968-1.052] | 0.652 |  | 0.524 | 0.997 | [0.889-1.117] | 0.953 |  |
| LD nonrobust glrlm ShortRunLowGrayLevelEmphasis | 0.523 | 1.034 | [0.992-1.078] | 0.112 |  | 0.564 | 0.95 | [0.846-1.067] | 0.394 |  |
| LD nonrobust glszm GrayLevelNonUniformityNormalized | 0.531 | 1.056 | [1.014-1.101] | 0.009 | ** | 0.579 | 1.031 | [0.913-1.163] | 0.627 |  |
| LD nonrobust glszm GrayLevelVariance | 0.520 | 0.984 | [0.943-1.025] | 0.433 |  | 0.598 | 0.946 | [0.85-1.052] | 0.308 |  |
| LD nonrobust glszm HighGrayLevelZoneEmphasis | 0.509 | 0.991 | [0.951-1.033] | 0.675 |  | 0.514 | 1.01 | [0.901-1.133] | 0.860 |  |
| LD nonrobust glszm LargeAreaEmphasis | 0.599 | 1.029 | [0.988-1.073] | 0.171 |  | 0.526 | 1.011 | [0.905-1.129] | 0.850 |  |
| LD nonrobust glszm LargeAreaHighGrayLevelEmphasis | 0.585 | 1.053 | [1.01-1.098] | 0.014 | * | 0.519 | 1.027 | [0.87-1.212] | 0.753 |  |
| LD nonrobust glszm LargeAreaLowGrayLevelEmphasis | 0.594 | 1.024 | [0.982-1.067] | 0.270 |  | 0.536 | 1.005 | [0.863-1.171] | 0.946 |  |
| LD nonrobust glszm LowGrayLevelZoneEmphasis | 0.511 | 1.031 | [0.989-1.074] | 0.154 |  | 0.598 | 0.937 | [0.837-1.048] | 0.261 |  |
| LD nonrobust glszm SizeZoneNonUniformity | 0.541 | 1.023 | [0.981-1.066] | 0.282 |  | 0.479 | 1.016 | [0.903-1.144] | 0.793 |  |
| LD nonrobust glszm SizeZoneNonUniformityNormalized | 0.534 | 0.964 | [0.925-1.005] | 0.087 |  | 0.619 | 0.938 | [0.839-1.049] | 0.267 |  |
| LD nonrobust glszm SmallAreaEmphasis | 0.534 | 0.955 | [0.916-0.995] | 0.028 | * | 0.617 | 0.94 | [0.826-1.07] | 0.354 |  |
| LD nonrobust glszm SmallAreaHighGrayLevelEmphasis | 0.506 | 0.984 | [0.944-1.026] | 0.448 |  | 0.536 | 0.998 | [0.891-1.118] | 0.971 |  |
| LD nonrobust glszm SmallAreaLowGrayLevelEmphasis | 0.528 | 1.004 | [0.963-1.047] | 0.837 |  | 0.617 | 0.933 | [0.84-1.037] | 0.207 |  |
| LD nonrobust glszm ZoneEntropy | 0.489 | 1.007 | [0.966-1.05] | 0.735 |  | 0.564 | 1.041 | [0.923-1.174] | 0.517 |  |
| LD nonrobust glszm ZoneVariance | 0.598 | 1.029 | [0.987-1.073] | 0.173 |  | 0.519 | 1.011 | [0.905-1.129] | 0.851 |  |
| LD nonrobust ngtdm Busyness | 0.556 | 1.07 | [1.027-1.115] | 0.001 | ** | 0.538 | 1.019 | [0.907-1.146] | 0.749 |  |
| LD nonrobust ngtdm Coarseness | 0.570 | 0.951 | [0.912-0.991] | 0.018 | * | 0.500 | 0.993 | [0.883-1.115] | 0.900 |  |
| LD nonrobust ngtdm Complexity | 0.565 | 0.947 | [0.909-0.987] | 0.011 | * | 0.590 | 0.942 | [0.839-1.057] | 0.312 |  |
| LD nonrobust ngtdm Contrast | 0.561 | 0.953 | [0.914-0.993] | 0.023 | * | 0.598 | 0.944 | [0.846-1.053] | 0.304 |  |

Supplementary Table S5b - Univariate analysis of radiomic features to predict regional or distant recurrence at 3 years

|  | **Center A** | | | | | **Center B** | | | | |
| --- | --- | --- | --- | --- | --- | --- | --- | --- | --- | --- |
| **feature** | **Concordance** | **HR** | **CI** | **p-val** | **sign** | **Concordance** | **HR** | **CI** | **p-val** | **sign** |
| Robust shape LeastAxisLength | 0.593 | 1.253 | [1.085-1.448] | 0.002 | ** | 0.614 | 1.326 | [1.085-1.448] | 0.362 |  |
| Robust shape Maximum2DDiameterSlice | 0.584 | 1.243 | [1.08-1.429] | 0.002 | ** | 0.6 | 1.422 | [1.08-1.429] | 0.235 |  |
| Robust shape MinorAxisLength | 0.598 | 1.298 | [1.119-1.506] | 0.001 | *** | 0.59 | 1.259 | [1.119-1.506] | 0.435 |  |
| Robust shape SurfaceArea | 0.588 | 1.26 | [1.088-1.459] | 0.002 | ** | 0.6 | 1.396 | [1.088-1.459] | 0.303 |  |
| Robust shape SurfaceVolumeRatio | 0.604 | 0.749 | [0.644-0.87] | 0.000 | *** | 0.612 | 0.74 | [0.644-0.87] | 0.350 |  |
| Robust glcm DifferenceAverage | 0.593 | 0.781 | [0.677-0.902] | 0.001 | *** | 0.595 | 0.675 | [0.677-0.902] | 0.313 |  |
| Robust glcm Id | 0.608 | 1.389 | [1.215-1.588] | 0.000 | *** | 0.587 | 1.237 | [1.215-1.588] | 0.492 |  |
| Robust glcm Idm | 0.61 | 1.395 | [1.222-1.594] | 0.000 | *** | 0.592 | 1.2 | [1.222-1.594] | 0.548 |  |
| Robust glcm Idn | 0.596 | 1.291 | [1.119-1.49] | 0.000 | *** | 0.597 | 1.46 | [1.119-1.49] | 0.324 |  |
| Robust glcm InverseVariance | 0.595 | 1.27 | [1.11-1.453] | 0.001 | *** | 0.58 | 1.213 | [1.11-1.453] | 0.532 |  |
| Robust gldm DependenceNonUniformityNormalized | 0.607 | 0.757 | [0.652-0.878] | 0.000 | *** | 0.592 | 0.752 | [0.652-0.878] | 0.403 |  |
| Robust gldm GrayLevelNonUniformity | 0.605 | 1.259 | [1.167-1.359] | 0.000 | *** | 0.58 | 1.107 | [1.167-1.359] | 0.786 |  |
| Robust gldm SmallDependenceEmphasis | 0.603 | 0.755 | [0.654-0.871] | 0.000 | *** | 0.619 | 0.695 | [0.654-0.871] | 0.304 |  |
| Robust glrlm GrayLevelNonUniformity | 0.602 | 1.3 | [1.183-1.429] | 0.000 | *** | 0.575 | 1.08 | [1.183-1.429] | 0.791 |  |
| Robust glrlm RunLengthNonUniformityNormalized | 0.609 | 0.716 | [0.628-0.817] | 0.000 | *** | 0.585 | 0.849 | [0.628-0.817] | 0.587 |  |
| Robust glrlm RunPercentage | 0.615 | 0.69 | [0.608-0.784] | 0.000 | *** | 0.585 | 0.846 | [0.608-0.784] | 0.573 |  |
| Robust glrlm ShortRunEmphasis | 0.609 | 0.71 | [0.625-0.806] | 0.000 | *** | 0.585 | 0.859 | [0.625-0.806] | 0.608 |  |
| Robust glszm GrayLevelNonUniformity | 0.578 | 1.211 | [1.047-1.401] | 0.010 | ** | 0.59 | 1.351 | [1.047-1.401] | 0.358 |  |
| Robust glszm ZonePercentage | 0.604 | 0.753 | [0.656-0.865] | 0.000 | *** | 0.617 | 0.748 | [0.656-0.865] | 0.375 |  |
| HD nonrobust shape Elongation | 0.532 | 1.058 | [0.926-1.208] | 0.408 |  | 0.555 | 0.876 | [0.926-1.208] | 0.629 |  |
| HD nonrobust shape Flatness | 0.506 | 1.027 | [0.899-1.174] | 0.694 |  | 0.505 | 0.922 | [0.899-1.174] | 0.790 |  |
| HD nonrobust shape MajorAxisLength | 0.575 | 1.233 | [1.066-1.425] | 0.005 | ** | 0.627 | 1.471 | [1.066-1.425] | 0.266 |  |
| HD nonrobust shape Maximum2DDiameterColumn | 0.585 | 1.238 | [1.069-1.434] | 0.004 | ** | 0.591 | 1.387 | [1.069-1.434] | 0.315 |  |
| HD nonrobust shape Maximum2DDiameterRow | 0.576 | 1.248 | [1.079-1.444] | 0.003 | ** | 0.586 | 1.35 | [1.079-1.444] | 0.389 |  |
| HD nonrobust shape Maximum3DDiameter | 0.574 | 1.243 | [1.079-1.432] | 0.003 | ** | 0.602 | 1.37 | [1.079-1.432] | 0.317 |  |
| HD nonrobust shape MeshVolume | 0.595 | 1.292 | [1.116-1.497] | 0.001 | *** | 0.597 | 1.38 | [1.116-1.497] | 0.314 |  |
| HD nonrobust shape Sphericity | 0.517 | 1.046 | [0.905-1.209] | 0.541 |  | 0.515 | 0.803 | [0.905-1.209] | 0.530 |  |
| HD nonrobust shape VoxelVolume | 0.595 | 1.292 | [1.116-1.497] | 0.001 | *** | 0.597 | 1.38 | [1.116-1.497] | 0.314 |  |
| HD nonrobust firstorder 10Percentile | 0.585 | 1.212 | [1.048-1.401] | 0.010 | ** | 0.57 | 0.732 | [1.048-1.401] | 0.418 |  |
| HD nonrobust firstorder 90Percentile | 0.513 | 1.116 | [0.955-1.303] | 0.167 |  | 0.498 | 1.001 | [0.955-1.303] | 0.997 |  |
| HD nonrobust firstorder Energy | 0.54 | 1.131 | [1.002-1.276] | 0.047 | * | 0.639 | 1.566 | [1.002-1.276] | 0.073 |  |
| HD nonrobust firstorder Entropy | 0.608 | 0.744 | [0.656-0.844] | 0.000 | *** | 0.507 | 1.234 | [0.656-0.844] | 0.585 |  |
| HD nonrobust firstorder InterquartileRange | 0.569 | 0.833 | [0.72-0.963] | 0.013 | * | 0.57 | 1.441 | [0.72-0.963] | 0.315 |  |
| HD nonrobust firstorder Kurtosis | 0.585 | 1.248 | [1.138-1.368] | 0.000 | *** | 0.575 | 0.529 | [1.138-1.368] | 0.392 |  |
| HD nonrobust firstorder Maximum | 0.551 | 1.195 | [1.091-1.309] | 0.000 | *** | 0.555 | 0.841 | [1.091-1.309] | 0.635 |  |
| HD nonrobust firstorder MeanAbsoluteDeviation | 0.551 | 0.873 | [0.755-1.009] | 0.067 |  | 0.595 | 1.397 | [0.755-1.009] | 0.366 |  |
| HD nonrobust firstorder Mean | 0.601 | 1.257 | [1.076-1.469] | 0.004 | ** | 0.515 | 0.903 | [1.076-1.469] | 0.760 |  |
| HD nonrobust firstorder Median | 0.603 | 1.261 | [1.08-1.471] | 0.003 | ** | 0.507 | 0.944 | [1.08-1.471] | 0.865 |  |
| HD nonrobust firstorder Minimum | 0.489 | 1.046 | [0.902-1.213] | 0.554 |  | 0.597 | 0.768 | [0.902-1.213] | 0.456 |  |
| HD nonrobust firstorder Range | 0.534 | 1.195 | [1.085-1.316] | 0.000 | *** | 0.555 | 1.03 | [1.085-1.316] | 0.936 |  |
| HD nonrobust firstorder RobustMeanAbsoluteDeviation | 0.56 | 0.849 | [0.734-0.981] | 0.026 | * | 0.582 | 1.452 | [0.734-0.981] | 0.310 |  |
| HD nonrobust firstorder RootMeanSquared | 0.587 | 0.804 | [0.695-0.931] | 0.004 | ** | 0.53 | 1.219 | [0.695-0.931] | 0.562 |  |
| HD nonrobust firstorder Skewness | 0.427 | 1.009 | [0.862-1.18] | 0.915 |  | 0.502 | 1.122 | [0.862-1.18] | 0.738 |  |
| HD nonrobust firstorder TotalEnergy | 0.54 | 1.131 | [1.002-1.276] | 0.047 | * | 0.639 | 1.566 | [1.002-1.276] | 0.073 |  |
| HD nonrobust firstorder Uniformity | 0.614 | 1.381 | [1.234-1.546] | 0.000 | *** | 0.52 | 0.801 | [1.234-1.546] | 0.608 |  |
| HD nonrobust firstorder Variance | 0.54 | 0.939 | [0.81-1.09] | 0.409 |  | 0.587 | 1.425 | [0.81-1.09] | 0.354 |  |
| HD nonrobust glcm Autocorrelation | 0.559 | 1.037 | [0.896-1.199] | 0.626 |  | 0.478 | 0.972 | [0.896-1.199] | 0.926 |  |
| HD nonrobust glcm ClusterProminence | 0.538 | 0.922 | [0.792-1.073] | 0.295 |  | 0.647 | 1.623 | [0.792-1.073] | 0.132 |  |
| HD nonrobust glcm ClusterShade | 0.534 | 0.92 | [0.796-1.063] | 0.255 |  | 0.532 | 0.763 | [0.796-1.063] | 0.395 |  |
| HD nonrobust glcm ClusterTendency | 0.569 | 0.838 | [0.725-0.969] | 0.017 | * | 0.632 | 1.704 | [0.725-0.969] | 0.166 |  |
| HD nonrobust glcm Contrast | 0.582 | 0.823 | [0.71-0.955] | 0.010 | * | 0.59 | 0.63 | [0.71-0.955] | 0.293 |  |
| HD nonrobust glcm Correlation | 0.517 | 1.012 | [0.877-1.168] | 0.869 |  | 0.694 | 1.947 | [0.877-1.168] | 0.073 |  |
| HD nonrobust glcm DifferenceEntropy | 0.593 | 0.76 | [0.668-0.864] | 0.000 | *** | 0.577 | 0.746 | [0.668-0.864] | 0.396 |  |
| HD nonrobust glcm DifferenceVariance | 0.556 | 0.868 | [0.752-1.001] | 0.051 |  | 0.587 | 0.674 | [0.752-1.001] | 0.345 |  |
| HD nonrobust glcm Idmn | 0.583 | 1.222 | [1.055-1.417] | 0.008 | ** | 0.59 | 1.577 | [1.055-1.417] | 0.294 |  |
| HD nonrobust glcm Imc1 | 0.53 | 1.088 | [0.935-1.267] | 0.275 |  | 0.6 | 0.847 | [0.935-1.267] | 0.554 |  |
| HD nonrobust glcm Imc2 | 0.592 | 0.799 | [0.703-0.909] | 0.001 | *** | 0.617 | 1.618 | [0.703-0.909] | 0.319 |  |
| HD nonrobust glcm JointAverage | 0.561 | 1.079 | [0.935-1.246] | 0.299 |  | 0.493 | 0.917 | [0.935-1.246] | 0.784 |  |
| HD nonrobust glcm JointEnergy | 0.617 | 1.368 | [1.243-1.506] | 0.000 | *** | 0.455 | 0.888 | [1.243-1.506] | 0.752 |  |
| HD nonrobust glcm JointEntropy | 0.603 | 0.747 | [0.656-0.85] | 0.000 | *** | 0.512 | 1.202 | [0.656-0.85] | 0.628 |  |
| HD nonrobust glcm MCC | 0.494 | 0.993 | [0.86-1.148] | 0.929 |  | 0.689 | 2.049 | [0.86-1.148] | 0.085 |  |
| HD nonrobust glcm MaximumProbability | 0.61 | 1.428 | [1.284-1.587] | 0.000 | *** | 0.535 | 1.014 | [1.284-1.587] | 0.965 |  |
| HD nonrobust glcm SumAverage | 0.561 | 1.079 | [0.935-1.246] | 0.299 |  | 0.493 | 0.917 | [0.935-1.246] | 0.784 |  |
| HD nonrobust glcm SumEntropy | 0.607 | 0.744 | [0.655-0.846] | 0.000 | *** | 0.537 | 1.37 | [0.655-0.846] | 0.467 |  |
| HD nonrobust glcm SumSquares | 0.571 | 0.832 | [0.721-0.96] | 0.012 | * | 0.617 | 1.435 | [0.721-0.96] | 0.295 |  |
| HD nonrobust gldm DependenceEntropy | 0.519 | 1.01 | [0.875-1.165] | 0.891 |  | 0.704 | 1.705 | [0.875-1.165] | 0.099 |  |
| HD nonrobust gldm DependenceNonUniformity | 0.557 | 1.158 | [1.002-1.338] | 0.046 | * | 0.612 | 1.356 | [1.002-1.338] | 0.307 |  |
| HD nonrobust gldm DependenceVariance | 0.611 | 1.483 | [1.31-1.678] | 0.000 | *** | 0.572 | 1.176 | [1.31-1.678] | 0.564 |  |
| HD nonrobust gldm GrayLevelVariance | 0.56 | 0.837 | [0.727-0.963] | 0.013 | * | 0.604 | 1.346 | [0.727-0.963] | 0.361 |  |
| HD nonrobust gldm HighGrayLevelEmphasis | 0.566 | 1.06 | [0.916-1.227] | 0.432 |  | 0.47 | 0.984 | [0.916-1.227] | 0.957 |  |
| HD nonrobust gldm LargeDependenceEmphasis | 0.615 | 1.469 | [1.31-1.648] | 0.000 | *** | 0.575 | 1.153 | [1.31-1.648] | 0.625 |  |
| HD nonrobust gldm LargeDependenceHighGrayLevelEmphasis | 0.591 | 1.295 | [1.133-1.479] | 0.000 | *** | 0.585 | 1.129 | [1.133-1.479] | 0.667 |  |
| HD nonrobust gldm LargeDependenceLowGrayLevelEmphasis | 0.548 | 1.188 | [1.09-1.295] | 0.000 | *** | 0.59 | 23.979 | [1.09-1.295] | 0.184 |  |
| HD nonrobust gldm LowGrayLevelEmphasis | 0.427 | 1.191 | [1.078-1.316] | 0.001 | *** | 0.505 | 0.751 | [1.078-1.316] | 0.531 |  |
| HD nonrobust gldm SmallDependenceHighGrayLevelEmphasis | 0.572 | 0.835 | [0.725-0.961] | 0.012 | * | 0.592 | 0.721 | [0.725-0.961] | 0.390 |  |
| HD nonrobust gldm SmallDependenceLowGrayLevelEmphasis | 0.585 | 0.801 | [0.667-0.961] | 0.017 | * | 0.597 | 0.646 | [0.667-0.961] | 0.371 |  |
| HD nonrobust glrlm GrayLevelNonUniformityNormalized | 0.602 | 1.334 | [1.185-1.502] | 0.000 | *** | 0.532 | 0.721 | [1.185-1.502] | 0.510 |  |
| HD nonrobust glrlm GrayLevelVariance | 0.555 | 0.842 | [0.734-0.967] | 0.015 | * | 0.602 | 1.319 | [0.734-0.967] | 0.385 |  |
| HD nonrobust glrlm HighGrayLevelRunEmphasis | 0.565 | 1.055 | [0.912-1.22] | 0.472 |  | 0.48 | 0.958 | [0.912-1.22] | 0.889 |  |
| HD nonrobust glrlm LongRunEmphasis | 0.616 | 1.429 | [1.293-1.578] | 0.000 | *** | 0.592 | 1.163 | [1.293-1.578] | 0.619 |  |
| HD nonrobust glrlm LongRunHighGrayLevelEmphasis | 0.584 | 1.286 | [1.131-1.461] | 0.000 | *** | 0.552 | 1.059 | [1.131-1.461] | 0.849 |  |
| HD nonrobust glrlm LongRunLowGrayLevelEmphasis | 0.444 | 1.164 | [1.073-1.262] | 0.000 | *** | 0.47 | 0.543 | [1.073-1.262] | 0.632 |  |
| HD nonrobust glrlm LowGrayLevelRunEmphasis | 0.43 | 1.135 | [0.993-1.299] | 0.064 |  | 0.5 | 0.779 | [0.993-1.299] | 0.532 |  |
| HD nonrobust glrlm RunEntropy | 0.564 | 0.828 | [0.731-0.939] | 0.003 | ** | 0.659 | 2.393 | [0.731-0.939] | 0.145 |  |
| HD nonrobust glrlm RunLengthNonUniformity | 0.582 | 1.229 | [1.062-1.423] | 0.006 | ** | 0.604 | 1.375 | [1.062-1.423] | 0.306 |  |
| HD nonrobust glrlm RunVariance | 0.617 | 1.42 | [1.292-1.562] | 0.000 | *** | 0.59 | 1.19 | [1.292-1.562] | 0.598 |  |
| HD nonrobust glrlm ShortRunHighGrayLevelEmphasis | 0.452 | 0.999 | [0.867-1.151] | 0.987 |  | 0.5 | 0.928 | [0.867-1.151] | 0.810 |  |
| HD nonrobust glrlm ShortRunLowGrayLevelEmphasis | 0.43 | 1.019 | [0.885-1.173] | 0.795 |  | 0.507 | 0.771 | [0.885-1.173] | 0.513 |  |
| HD nonrobust glszm GrayLevelNonUniformityNormalized | 0.554 | 1.202 | [1.082-1.334] | 0.001 | *** | 0.495 | 0.883 | [1.082-1.334] | 0.719 |  |
| HD nonrobust glszm GrayLevelVariance | 0.548 | 0.887 | [0.769-1.024] | 0.101 |  | 0.507 | 0.869 | [0.769-1.024] | 0.663 |  |
| HD nonrobust glszm HighGrayLevelZoneEmphasis | 0.548 | 1.005 | [0.871-1.16] | 0.942 |  | 0.5 | 0.878 | [0.871-1.16] | 0.677 |  |
| HD nonrobust glszm LargeAreaEmphasis | 0.611 | 1.198 | [1.116-1.286] | 0.000 | *** | 0.595 | 1.036 | [1.116-1.286] | 0.897 |  |
| HD nonrobust glszm LargeAreaHighGrayLevelEmphasis | 0.602 | 1.168 | [1.081-1.261] | 0.000 | *** | 0.592 | 1.028 | [1.081-1.261] | 0.917 |  |
| HD nonrobust glszm LargeAreaLowGrayLevelEmphasis | 0.593 | 1.159 | [1.071-1.254] | 0.000 | *** | 0.609 | 2.243 | [1.071-1.254] | 0.616 |  |
| HD nonrobust glszm LowGrayLevelZoneEmphasis | 0.446 | 1.055 | [0.93-1.198] | 0.406 |  | 0.52 | 0.782 | [0.93-1.198] | 0.531 |  |
| HD nonrobust glszm SizeZoneNonUniformity | 0.565 | 1.135 | [0.978-1.318] | 0.096 |  | 0.555 | 1.253 | [0.978-1.318] | 0.470 |  |
| HD nonrobust glszm SizeZoneNonUniformityNormalized | 0.55 | 0.849 | [0.745-0.968] | 0.014 | * | 0.614 | 0.586 | [0.745-0.968] | 0.155 |  |
| HD nonrobust glszm SmallAreaEmphasis | 0.55 | 0.848 | [0.747-0.963] | 0.011 | * | 0.617 | 0.611 | [0.747-0.963] | 0.146 |  |
| HD nonrobust glszm SmallAreaHighGrayLevelEmphasis | 0.54 | 1.017 | [0.885-1.168] | 0.817 |  | 0.552 | 0.754 | [0.885-1.168] | 0.427 |  |
| HD nonrobust glszm SmallAreaLowGrayLevelEmphasis | 0.448 | 1.007 | [0.875-1.159] | 0.924 |  | 0.565 | 0.716 | [0.875-1.159] | 0.437 |  |
| HD nonrobust glszm ZoneEntropy | 0.521 | 1.036 | [0.907-1.183] | 0.604 |  | 0.649 | 1.616 | [0.907-1.183] | 0.147 |  |
| HD nonrobust glszm ZoneVariance | 0.611 | 1.198 | [1.115-1.286] | 0.000 | *** | 0.59 | 1.036 | [1.115-1.286] | 0.900 |  |
| HD nonrobust ngtdm Busyness | 0.576 | 1.263 | [1.165-1.369] | 0.000 | *** | 0.58 | 1.237 | [1.165-1.369] | 0.502 |  |
| HD nonrobust ngtdm Coarseness | 0.601 | 0.825 | [0.697-0.976] | 0.025 | * | 0.562 | 0.88 | [0.697-0.976] | 0.699 |  |
| HD nonrobust ngtdm Complexity | 0.581 | 0.804 | [0.696-0.929] | 0.003 | ** | 0.677 | 0.461 | [0.696-0.929] | 0.086 |  |
| HD nonrobust ngtdm Contrast | 0.583 | 0.82 | [0.709-0.949] | 0.008 | ** | 0.517 | 0.785 | [0.709-0.949] | 0.529 |  |
| LD nonrobust shape Elongation | 0.484 | 0.973 | [0.848-1.118] | 0.702 |  | 0.55 | 0.931 | [0.848-1.118] | 0.810 |  |
| LD nonrobust shape Flatness | 0.513 | 0.875 | [0.763-1.004] | 0.056 |  | 0.597 | 0.801 | [0.763-1.004] | 0.478 |  |
| LD nonrobust shape MajorAxisLength | 0.577 | 1.295 | [1.122-1.493] | 0.000 | *** | 0.58 | 1.243 | [1.122-1.493] | 0.472 |  |
| LD nonrobust shape Maximum2DDiameterColumn | 0.582 | 1.307 | [1.133-1.507] | 0.000 | *** | 0.56 | 1.198 | [1.133-1.507] | 0.541 |  |
| LD nonrobust shape Maximum2DDiameterRow | 0.58 | 1.293 | [1.123-1.489] | 0.000 | *** | 0.544 | 1.198 | [1.123-1.489] | 0.540 |  |
| LD nonrobust shape Maximum3DDiameter | 0.575 | 1.275 | [1.106-1.471] | 0.001 | *** | 0.588 | 1.358 | [1.106-1.471] | 0.307 |  |
| LD nonrobust shape MeshVolume | 0.576 | 1.376 | [1.222-1.55] | 0.000 | *** | 0.57 | 1.158 | [1.222-1.55] | 0.584 |  |
| LD nonrobust shape Sphericity | 0.528 | 0.951 | [0.829-1.092] | 0.475 |  | 0.547 | 0.831 | [0.829-1.092] | 0.544 |  |
| LD nonrobust shape VoxelVolume | 0.576 | 1.376 | [1.222-1.55] | 0.000 | *** | 0.57 | 1.158 | [1.222-1.55] | 0.584 |  |
| LD nonrobust firstorder 10Percentile | 0.55 | 1.104 | [0.964-1.264] | 0.154 |  | 0.535 | 1.077 | [0.964-1.264] | 0.816 |  |
| LD nonrobust firstorder 90Percentile | 0.57 | 1.052 | [0.909-1.218] | 0.495 |  | 0.512 | 1.055 | [0.909-1.218] | 0.897 |  |
| LD nonrobust firstorder Energy | 0.552 | 1.246 | [1.108-1.401] | 0.000 | *** | 0.575 | 1.248 | [1.108-1.401] | 0.398 |  |
| LD nonrobust firstorder Entropy | 0.577 | 0.824 | [0.736-0.921] | 0.001 | *** | 0.552 | 0.892 | [0.736-0.921] | 0.703 |  |
| LD nonrobust firstorder InterquartileRange | 0.533 | 0.906 | [0.787-1.043] | 0.168 |  | 0.542 | 0.884 | [0.787-1.043] | 0.685 |  |
| LD nonrobust firstorder Kurtosis | 0.545 | 1.086 | [0.984-1.199] | 0.102 |  | 0.54 | 1.173 | [0.984-1.199] | 0.625 |  |
| LD nonrobust firstorder Maximum | 0.551 | 1.114 | [1.007-1.232] | 0.036 | * | 0.562 | 1.261 | [1.007-1.232] | 0.538 |  |
| LD nonrobust firstorder MeanAbsoluteDeviation | 0.513 | 0.963 | [0.838-1.108] | 0.601 |  | 0.515 | 0.923 | [0.838-1.108] | 0.798 |  |
| LD nonrobust firstorder Mean | 0.567 | 1.119 | [0.966-1.296] | 0.133 |  | 0.532 | 1.045 | [0.966-1.296] | 0.900 |  |
| LD nonrobust firstorder Median | 0.569 | 1.127 | [0.973-1.304] | 0.110 |  | 0.547 | 1.035 | [0.973-1.304] | 0.919 |  |
| LD nonrobust firstorder Minimum | 0.518 | 0.994 | [0.857-1.153] | 0.941 |  | 0.55 | 0.754 | [0.857-1.153] | 0.381 |  |
| LD nonrobust firstorder Range | 0.536 | 1.116 | [1.004-1.24] | 0.042 | * | 0.572 | 1.419 | [1.004-1.24] | 0.341 |  |
| LD nonrobust firstorder RobustMeanAbsoluteDeviation | 0.524 | 0.931 | [0.809-1.07] | 0.314 |  | 0.537 | 0.893 | [0.809-1.07] | 0.715 |  |
| LD nonrobust firstorder RootMeanSquared | 0.559 | 0.911 | [0.788-1.054] | 0.210 |  | 0.537 | 0.982 | [0.788-1.054] | 0.957 |  |
| LD nonrobust firstorder Skewness | 0.557 | 0.959 | [0.84-1.094] | 0.532 |  | 0.54 | 0.891 | [0.84-1.094] | 0.713 |  |
| LD nonrobust firstorder TotalEnergy | 0.552 | 1.246 | [1.108-1.401] | 0.000 | *** | 0.575 | 1.248 | [1.108-1.401] | 0.398 |  |
| LD nonrobust firstorder Uniformity | 0.59 | 1.167 | [1.053-1.294] | 0.003 | ** | 0.56 | 1.126 | [1.053-1.294] | 0.682 |  |
| LD nonrobust firstorder Variance | 0.496 | 1.013 | [0.875-1.173] | 0.860 |  | 0.507 | 0.95 | [0.875-1.173] | 0.871 |  |
| LD nonrobust glcm Autocorrelation | 0.544 | 1.022 | [0.887-1.178] | 0.763 |  | 0.527 | 1.023 | [0.887-1.178] | 0.944 |  |
| LD nonrobust glcm ClusterProminence | 0.508 | 1.024 | [0.88-1.19] | 0.762 |  | 0.58 | 0.712 | [0.88-1.19] | 0.316 |  |
| LD nonrobust glcm ClusterShade | 0.547 | 0.928 | [0.8-1.076] | 0.320 |  | 0.532 | 1.187 | [0.8-1.076] | 0.567 |  |
| LD nonrobust glcm ClusterTendency | 0.516 | 0.903 | [0.785-1.038] | 0.150 |  | 0.555 | 0.817 | [0.785-1.038] | 0.507 |  |
| LD nonrobust glcm Contrast | 0.559 | 0.854 | [0.741-0.983] | 0.028 | * | 0.595 | 0.702 | [0.741-0.983] | 0.272 |  |
| LD nonrobust glcm Correlation | 0.543 | 1.066 | [0.921-1.233] | 0.393 |  | 0.582 | 1.497 | [0.921-1.233] | 0.255 |  |
| LD nonrobust glcm DifferenceEntropy | 0.566 | 0.827 | [0.717-0.954] | 0.009 | ** | 0.597 | 0.719 | [0.717-0.954] | 0.299 |  |
| LD nonrobust glcm DifferenceVariance | 0.532 | 0.918 | [0.795-1.061] | 0.245 |  | 0.6 | 0.591 | [0.795-1.061] | 0.189 |  |
| LD nonrobust glcm Idmn | 0.559 | 1.145 | [0.988-1.328] | 0.072 |  | 0.595 | 1.522 | [0.988-1.328] | 0.254 |  |
| LD nonrobust glcm Imc1 | 0.496 | 0.928 | [0.814-1.057] | 0.261 |  | 0.517 | 1.171 | [0.814-1.057] | 0.644 |  |
| LD nonrobust glcm Imc2 | 0.558 | 0.926 | [0.822-1.044] | 0.211 |  | 0.537 | 0.918 | [0.822-1.044] | 0.773 |  |
| LD nonrobust glcm JointAverage | 0.545 | 1.029 | [0.894-1.186] | 0.688 |  | 0.52 | 1.035 | [0.894-1.186] | 0.917 |  |
| LD nonrobust glcm JointEnergy | 0.6 | 1.127 | [1.022-1.242] | 0.016 | * | 0.552 | 1.203 | [1.022-1.242] | 0.511 |  |
| LD nonrobust glcm JointEntropy | 0.581 | 0.807 | [0.72-0.903] | 0.000 | *** | 0.525 | 0.944 | [0.72-0.903] | 0.854 |  |
| LD nonrobust glcm MCC | 0.556 | 1.108 | [0.955-1.284] | 0.175 |  | 0.619 | 1.586 | [0.955-1.284] | 0.195 |  |
| LD nonrobust glcm MaximumProbability | 0.599 | 1.163 | [1.045-1.294] | 0.006 | ** | 0.55 | 1.184 | [1.045-1.294] | 0.523 |  |
| LD nonrobust glcm SumAverage | 0.545 | 1.029 | [0.894-1.186] | 0.688 |  | 0.52 | 1.035 | [0.894-1.186] | 0.917 |  |
| LD nonrobust glcm SumEntropy | 0.581 | 0.827 | [0.738-0.926] | 0.001 | *** | 0.532 | 0.91 | [0.738-0.926] | 0.750 |  |
| LD nonrobust glcm SumSquares | 0.52 | 0.895 | [0.779-1.027] | 0.114 |  | 0.567 | 0.79 | [0.779-1.027] | 0.430 |  |
| LD nonrobust gldm DependenceEntropy | 0.473 | 0.999 | [0.869-1.15] | 0.992 |  | 0.562 | 1.315 | [0.869-1.15] | 0.399 |  |
| LD nonrobust gldm DependenceNonUniformity | 0.562 | 1.245 | [1.083-1.431] | 0.002 | ** | 0.552 | 1.208 | [1.083-1.431] | 0.464 |  |
| LD nonrobust gldm DependenceVariance | 0.589 | 1.248 | [1.101-1.415] | 0.001 | *** | 0.552 | 1.225 | [1.101-1.415] | 0.428 |  |
| LD nonrobust gldm GrayLevelVariance | 0.514 | 0.901 | [0.786-1.033] | 0.136 |  | 0.585 | 0.752 | [0.786-1.033] | 0.343 |  |
| LD nonrobust gldm HighGrayLevelEmphasis | 0.547 | 1.036 | [0.899-1.195] | 0.623 |  | 0.525 | 1.06 | [0.899-1.195] | 0.860 |  |
| LD nonrobust gldm LargeDependenceEmphasis | 0.593 | 1.226 | [1.097-1.37] | 0.000 | *** | 0.575 | 1.202 | [1.097-1.37] | 0.487 |  |
| LD nonrobust gldm LargeDependenceHighGrayLevelEmphasis | 0.572 | 1.249 | [1.095-1.425] | 0.001 | *** | 0.56 | 1.226 | [1.095-1.425] | 0.429 |  |
| LD nonrobust gldm LargeDependenceLowGrayLevelEmphasis | 0.55 | 1.119 | [1.032-1.214] | 0.007 | ** | 0.545 | 1.043 | [1.032-1.214] | 0.843 |  |
| LD nonrobust gldm LowGrayLevelEmphasis | 0.46 | 1.126 | [1.018-1.244] | 0.021 | * | 0.6 | 0.642 | [1.018-1.244] | 0.315 |  |
| LD nonrobust gldm SmallDependenceHighGrayLevelEmphasis | 0.559 | 0.835 | [0.732-0.953] | 0.008 | ** | 0.59 | 0.781 | [0.732-0.953] | 0.460 |  |
| LD nonrobust gldm SmallDependenceLowGrayLevelEmphasis | 0.568 | 0.93 | [0.803-1.076] | 0.328 |  | 0.629 | 0.5 | [0.803-1.076] | 0.224 |  |
| LD nonrobust glrlm GrayLevelNonUniformityNormalized | 0.579 | 1.165 | [1.051-1.293] | 0.004 | ** | 0.552 | 1.072 | [1.051-1.293] | 0.823 |  |
| LD nonrobust glrlm GrayLevelVariance | 0.506 | 0.968 | [0.838-1.117] | 0.656 |  | 0.592 | 0.669 | [0.838-1.117] | 0.228 |  |
| LD nonrobust glrlm HighGrayLevelRunEmphasis | 0.544 | 1.023 | [0.888-1.177] | 0.754 |  | 0.505 | 1.046 | [0.888-1.177] | 0.891 |  |
| LD nonrobust glrlm LongRunEmphasis | 0.593 | 1.175 | [1.064-1.298] | 0.001 | ** | 0.582 | 1.217 | [1.064-1.298] | 0.477 |  |
| LD nonrobust glrlm LongRunHighGrayLevelEmphasis | 0.566 | 1.217 | [1.06-1.398] | 0.005 | ** | 0.54 | 1.203 | [1.06-1.398] | 0.500 |  |
| LD nonrobust glrlm LongRunLowGrayLevelEmphasis | 0.477 | 1.119 | [1.027-1.219] | 0.010 | ** | 0.59 | 0.672 | [1.027-1.219] | 0.313 |  |
| LD nonrobust glrlm LowGrayLevelRunEmphasis | 0.464 | 1.121 | [1.006-1.249] | 0.039 | * | 0.6 | 0.608 | [1.006-1.249] | 0.294 |  |
| LD nonrobust glrlm RunEntropy | 0.527 | 0.883 | [0.789-0.987] | 0.028 | * | 0.495 | 0.997 | [0.789-0.987] | 0.993 |  |
| LD nonrobust glrlm RunLengthNonUniformity | 0.571 | 1.353 | [1.185-1.545] | 0.000 | *** | 0.577 | 1.173 | [1.185-1.545] | 0.548 |  |
| LD nonrobust glrlm RunVariance | 0.592 | 1.165 | [1.056-1.287] | 0.002 | ** | 0.582 | 1.233 | [1.056-1.287] | 0.422 |  |
| LD nonrobust glrlm ShortRunHighGrayLevelEmphasis | 0.528 | 1.005 | [0.878-1.151] | 0.940 |  | 0.502 | 1.01 | [0.878-1.151] | 0.975 |  |
| LD nonrobust glrlm ShortRunLowGrayLevelEmphasis | 0.461 | 1.109 | [0.982-1.253] | 0.094 |  | 0.597 | 0.606 | [0.982-1.253] | 0.290 |  |
| LD nonrobust glszm GrayLevelNonUniformityNormalized | 0.513 | 1.106 | [0.996-1.228] | 0.060 |  | 0.577 | 1.205 | [0.996-1.228] | 0.543 |  |
| LD nonrobust glszm GrayLevelVariance | 0.505 | 0.974 | [0.844-1.126] | 0.725 |  | 0.6 | 0.685 | [0.844-1.126] | 0.230 |  |
| LD nonrobust glszm HighGrayLevelZoneEmphasis | 0.483 | 0.957 | [0.837-1.093] | 0.513 |  | 0.507 | 1.101 | [0.837-1.093] | 0.774 |  |
| LD nonrobust glszm LargeAreaEmphasis | 0.594 | 1.077 | [0.968-1.199] | 0.173 |  | 0.57 | 1.056 | [0.968-1.199] | 0.845 |  |
| LD nonrobust glszm LargeAreaHighGrayLevelEmphasis | 0.585 | 1.173 | [1.087-1.265] | 0.000 | *** | 0.557 | 1.143 | [1.087-1.265] | 0.742 |  |
| LD nonrobust glszm LargeAreaLowGrayLevelEmphasis | 0.582 | 1.032 | [0.923-1.154] | 0.582 |  | 0.577 | 1.028 | [0.923-1.154] | 0.945 |  |
| LD nonrobust glszm LowGrayLevelZoneEmphasis | 0.469 | 1.067 | [0.946-1.204] | 0.292 |  | 0.619 | 0.492 | [0.946-1.204] | 0.193 |  |
| LD nonrobust glszm SizeZoneNonUniformity | 0.563 | 1.076 | [0.927-1.247] | 0.336 |  | 0.565 | 1.175 | [0.927-1.247] | 0.610 |  |
| LD nonrobust glszm SizeZoneNonUniformityNormalized | 0.532 | 0.901 | [0.784-1.037] | 0.145 |  | 0.622 | 0.669 | [0.784-1.037] | 0.208 |  |
| LD nonrobust glszm SmallAreaEmphasis | 0.532 | 0.887 | [0.778-1.01] | 0.071 |  | 0.619 | 0.691 | [0.778-1.01] | 0.282 |  |
| LD nonrobust glszm SmallAreaHighGrayLevelEmphasis | 0.492 | 0.938 | [0.821-1.072] | 0.346 |  | 0.483 | 1.006 | [0.821-1.072] | 0.986 |  |
| LD nonrobust glszm SmallAreaLowGrayLevelEmphasis | 0.455 | 1.002 | [0.874-1.149] | 0.977 |  | 0.624 | 0.416 | [0.874-1.149] | 0.157 |  |
| LD nonrobust glszm ZoneEntropy | 0.539 | 1.025 | [0.892-1.178] | 0.728 |  | 0.577 | 1.341 | [0.892-1.178] | 0.381 |  |
| LD nonrobust glszm ZoneVariance | 0.593 | 1.077 | [0.968-1.199] | 0.173 |  | 0.565 | 1.056 | [0.968-1.199] | 0.846 |  |
| LD nonrobust ngtdm Busyness | 0.562 | 1.18 | [1.071-1.3] | 0.001 | *** | 0.56 | 1.142 | [1.071-1.3] | 0.646 |  |
| LD nonrobust ngtdm Coarseness | 0.579 | 0.867 | [0.748-1.006] | 0.059 |  | 0.55 | 0.89 | [0.748-1.006] | 0.716 |  |
| LD nonrobust ngtdm Complexity | 0.563 | 0.86 | [0.745-0.992] | 0.038 | * | 0.602 | 0.624 | [0.745-0.992] | 0.233 |  |
| LD nonrobust ngtdm Contrast | 0.551 | 0.878 | [0.76-1.014] | 0.077 |  | 0.597 | 0.665 | [0.76-1.014] | 0.237 |  |

Supplementary Table S5c – Univariate analysis of radiomic features to predict regional or distant progression free survival.

| **feat. group** | **formula** | **AUC train** | **AUC int. val.** | **AUC ext. val.** |
| --- | --- | --- | --- | --- |
| all | rdr ~ LD shape Flatness+  LD gldm SmallDependenceLowGrayLevelEmphasis | 0.59 | 0.55 | 0.68 |
|  | rdr ~ HD firstorder 90Percentile+  LD glszm LargeAreaLowGrayLevelEmphasis | 0.56 | 0.53 | 0.64 |
|  | rdr ~ LD firstorder Minimum+  HD glcm ClusterProminence | 0.57 | 0.56 | 0.65 |
|  | rdr ~ LD glszm LargeAreaLowGrayLevelEmphasis+  HD firstorder Minimum | 0.47 | 0.57 | 0.52 |
|  | rdr ~ HD firstorder 90Percentile+  LD firstorder 90Percentile | 0.58 | 0.50 | 0.53 |
|  | rdr ~ HD firstorder Median | 0.59 | 0.49 | 0.61 |
|  | rdr ~ HD firstorder 90Percentile+  LD glcm Imc2 | 0.56 | 0.51 | 0.50 |
|  | rdr ~ HD gldm SmallDependenceLowGrayLevelEmphasis+  LD shape Flatness | 0.57 | 0.62 | 0.61 |
|  | rdr ~ HD firstorder Median+  HD firstorder Mean | 0.60 | 0.49 | 0.58 |
|  | rdr ~ LD glszm LargeAreaLowGrayLevelEmphasis+  HD glszm ZoneEntropy+LD firstorder 90Percentile+  LD shape Flatness | 0.62 | 0.53 | 0.55 |
| HD | rdr ~ HD glszm SmallAreaHighGrayLevelEmphasis+  HD firstorder Median | 0.56 | 0.59 | 0.72 |
|  | rdr ~ HD firstorder Median+  HD shape Elongation | 0.58 | 0.52 | 0.60 |
|  | rdr ~ HD firstorder 90Percentile+  HD glszm SizeZoneNonUniformityNormalized+  HD glszm SmallAreaHighGrayLevelEmphasis | 0.60 | 0.54 | 0.81 |
|  | rdr ~ HD firstorder Median+  HD glszm SmallAreaHighGrayLevelEmphasis | 0.61 | 0.52 | 0.56 |
|  | rdr ~ HD gldm SmallDependenceLowGrayLevelEmphasis+  HD gldm DependenceEntropy | 0.56 | 0.51 | 0.54 |
|  | rdr ~ HD gldm SmallDependenceLowGrayLevelEmphasis+  HD glszm ZoneEntropy | 0.57 | 0.56 | 0.68 |
|  | rdr ~ HD glcm ClusterProminence+  HD gldm DependenceNonUniformity | 0.60 | 0.51 | 0.57 |
|  | rdr ~ HD firstorder Variance+HD glcm ClusterProminence+  HD firstorder RobustMeanAbsoluteDeviation | 0.61 | 0.54 | 0.62 |
|  | rdr ~ HD firstorder 90Percentile+  HD firstorder Variance+  HD shape Sphericity+  HD glszm SmallAreaHighGrayLevelEmphasis | 0.59 | 0.58 | 0.59 |
|  | rdr ~ HD glcm ClusterProminence+  HD firstorder Variance | 0.56 | 0.56 | 0.65 |
| LD | rdr ~ LD shape Flatness+  LD glszm SizeZoneNonUniformityNormalized+  LD firstorder Minimum | 0.60 | 0.50 | 0.64 |
|  | rdr ~ LD glszm ZoneVariance+  LD firstorder 90Percentile+  LD firstorder Minimum | 0.54 | 0.55 | 0.62 |
|  | rdr ~ LD firstorder Energy+  LD shape Sphericity+LD shape Flatness+  LD firstorder Median | 0.66 | 0.57 | 0.66 |
|  | rdr ~ LD firstorder Energy+  LD firstorder 90Percentile | 0.56 | 0.63 | 0.43 |
|  | rdr ~ LD glszm LargeAreaLowGrayLevelEmphasis+  LD shape Flatness | 0.56 | 0.57 | 0.62 |
|  | rdr ~ LD glszm LargeAreaLowGrayLevelEmphasis+  LD shape Elongation | 0.55 | 0.48 | 0.52 |
|  | rdr ~ LD shape Elongation+  LD glrlm ShortRunHighGrayLevelEmphasis+  LD glszm SizeZoneNonUniformityNormalized | 0.54 | 0.58 | 0.73 |
|  | rdr ~ LD firstorder 90Percentile+  LD shape Sphericity+  LD shape Flatness | 0.62 | 0.52 | 0.59 |
|  | rdr ~ LD glszm LargeAreaLowGrayLevelEmphasis+  LD gldm DependenceVariance | 0.54 | 0.66 | 0.68 |
|  | rdr ~ LD shape Elongation+  LD firstorder 90Percentile | 0.54 | 0.56 | 0.49 |
| robust | rdr ~ robust gldm DependenceNonUniformityNormalized+  robust gldm SmallDependenceEmphasis+  robust glcm Idn | 0.59 | 0.52 | 0.78 |
|  | rdr ~ robust ngtdm Coarseness+  robust shape LeastAxisLength+  robust glcm InverseVariance | 0.63 | 0.54 | 0.62 |
|  | rdr ~ robust ngtdm Coarseness+  robust shape Maximum2DDiameterSlice+  robust glszm ZonePercentage | 0.63 | 0.58 | 0.68 |
|  | rdr ~ robust ngtdm Coarseness+  robust glszm GrayLevelNonUniformity+  robust shape SurfaceVolumeRatio+  robust shape MinorAxisLength | 0.66 | 0.50 | 0.69 |
|  | rdr ~ robust gldm DependenceNonUniformityNormalized+  robust shape Maximum2DDiameterSlice+  robust gldm SmallDependenceEmphasis | 0.61 | 0.52 | 0.70 |
|  | rdr ~ robust ngtdm Coarseness+  robust shape SurfaceVolumeRatio+  robust glcm Idn | 0.62 | 0.59 | 0.80 |
|  | rdr ~ robust gldm DependenceNonUniformityNormalized+  robust shape SurfaceVolumeRatio+  robust shape LeastAxisLength+  robust glszm ZonePercentage | 0.61 | 0.51 | 0.66 |
|  | rdr ~ robust ngtdm Coarseness+  robust gldm SmallDependenceEmphasis+  robust glszm ZonePercentage+  robust glcm Idn | 0.67 | 0.54 | 0.77 |
|  | rdr ~ robust ngtdm Coarseness+  robust glszm ZonePercentage+  robust glcm Idn | 0.64 | 0.55 | 0.73 |
|  | rdr ~ robust ngtdm Coarseness+  robust gldm DependenceNonUniformityNormalized+  robust glszm GrayLevelNonUniformity+  robust shape SurfaceVolumeRatio+  robust gldm SmallDependenceEmphasis | 0.59 | 0.58 | 0.77 |

Supplementary Table S6a - Individual predictive performance of the selected model per repetition on the regional or distant recurrence endpoint

| feat. group | formula | AUC train | AUC int. val. | AUC ext. val. |
| --- | --- | --- | --- | --- |
| all | rdr3y ~ LD firstorder 90Percentile+  HD shape Elongation | 0.59 | 0.53 | 0.59 |
|  | rdr3y ~ HD firstorder Mean | 0.61 | 0.55 | 0.58 |
|  | rdr3y ~ LD glszm LargeAreaEmphasis+  HD firstorder 90Percentile+  HD glszm ZoneEntropy | 0.51 | 0.50 | 0.71 |
|  | rdr3y ~ LD glszm LargeAreaLowGrayLevelEmphasis+  HD shape Elongation+  LD shape Flatness | 0.67 | 0.51 | 0.53 |
|  | rdr3y ~ HD firstorder 90Percentile+  LD shape Flatness+  HD gldm SmallDependenceHighGrayLevelEmphasis | 0.64 | 0.48 | 0.71 |
|  | rdr3y ~ HD firstorder 90Percentile+  LD glszm LargeAreaLowGrayLevelEmphasis+  LD firstorder 90Percentile | 0.61 | 0.66 | 0.62 |
|  | rdr3y ~ HD gldm SmallDependenceLowGrayLevelEmphasis+  HD shape Sphericity | 0.57 | 0.49 | 0.42 |
|  | rdr3y ~ HD firstorder 90Percentile+  HD gldm SmallDependenceLowGrayLevelEmphasis | 0.59 | 0.54 | 0.57 |
|  | rdr3y ~ HD gldm SmallDependenceLowGrayLevelEmphasis+  LD shape Elongation+  HD glszm SmallAreaHighGrayLevelEmphasis+  HD glszm ZoneEntropy | 0.56 | 0.49 | 0.56 |
|  | rdr3y ~ HD firstorder 90Percentile+  HD shape Sphericity | 0.53 | 0.54 | 0.58 |
| HD | rdr3y ~ HD gldm SmallDependenceLowGrayLevelEmphasis+  HD glcm Imc1 | 0.61 | 0.50 | 0.67 |
|  | rdr3y ~ HD shape Elongation+  HD gldm SmallDependenceLowGrayLevelEmphasis | 0.58 | 0.52 | 0.55 |
|  | rdr3y ~ HD firstorder Mean | 0.56 | 0.66 | 0.58 |
|  | rdr3y ~ HD firstorder 90Percentile+  HD glszm SizeZoneNonUniformityNormalized | 0.61 | 0.54 | 0.70 |
|  | rdr3y ~ HD firstorder Energy+  HD glszm LargeAreaLowGrayLevelEmphasis+  HD firstorder 90Percentile | 0.48 | 0.46 | 0.66 |
|  | rdr3y ~ HD firstorder 90Percentile+  HD firstorder Energy+HD shape Flatness+  HD firstorder Mean | 0.62 | 0.54 | 0.62 |
|  | rdr3y ~ HD firstorder 90Percentile+  HD gldm SmallDependenceLowGrayLevelEmphasis | 0.59 | 0.50 | 0.57 |
|  | rdr3y ~ HD shape Elongation+  HD shape Sphericity+  HD firstorder Mean | 0.61 | 0.54 | 0.57 |
|  | rdr3y ~ HD firstorder 90Percentile+  HD firstorder Mean | 0.63 | 0.53 | 0.61 |
|  | rdr3y ~ HD glszm ZoneEntropy+  HD firstorder Variance | 0.61 | 0.50 | 0.62 |
| LD | rdr3y ~ LD firstorder 90Percentile | 0.59 | 0.63 | 0.49 |
|  | rdr3y ~ LD shape Sphericity+  LD shape Flatness | 0.54 | 0.46 | 0.62 |
|  | rdr3y ~ LD firstorder 90Percentile+  LD glszm LargeAreaLowGrayLevelEmphasis | 0.64 | 0.50 | 0.53 |
|  | rdr3y ~ LD firstorder 90Percentile+ LD shape Flatness+  LD glszm LargeAreaLowGrayLevelEmphasis | 0.63 | 0.49 | 0.57 |
|  | rdr3y ~ LD glszm LargeAreaLowGrayLevelEmphasis+  LD firstorder 90Percentile+ LD shape Elongation+  LD shape Flatness | 0.66 | 0.54 | 0.47 |
|  | rdr3y ~ LD glszm LargeAreaEmphasis | 0.63 | 0.60 | 0.60 |
|  | rdr3y ~ LD shape Flatness+  LD firstorder Mean | 0.63 | 0.55 | 0.58 |
|  | rdr3y ~ LD glcm MCC+  LD shape Sphericity | 0.57 | 0.51 | 0.65 |
|  | rdr3y ~ LD glszm SmallAreaHighGrayLevelEmphasis+  LD glrlm ShortRunHighGrayLevelEmphasis | 0.59 | 0.45 | 0.62 |
|  | rdr3y ~ LD shape Sphericity+  LD glszm LargeAreaLowGrayLevelEmphasis | 0.57 | 0.54 | 0.47 |
| robust | rdr3y ~ robust glszm ZonePercentage+  robust gldm SmallDependenceEmphasis | 0.64 | 0.46 | 0.74 |
|  | rdr3y ~ robust gldm DependenceNonUniformityNormalized+  robust shape LeastAxisLength+robust ngtdm Coarseness+  robust glcm InverseVariance | 0.66 | 0.54 | 0.70 |
|  | rdr3y ~ robust gldm SmallDependenceEmphasis+  robust ngtdm Coarseness+  robust shape LeastAxisLength+  robust shape SurfaceVolumeRatio | 0.70 | 0.52 | 0.71 |
|  | rdr3y ~ robust gldm DependenceNonUniformityNormalized+  robust ngtdm Coarseness+  robust shape LeastAxisLength+  robust shape SurfaceVolumeRatio+  robust glszm GrayLevelNonUniformity | 0.67 | 0.60 | 0.84 |
|  | rdr3y ~ robust ngtdm Coarseness+  robust glcm InverseVariance+  robust shape SurfaceVolumeRatio | 0.67 | 0.56 | 0.74 |
|  | rdr3y ~ robust ngtdm Coarseness+  robust glszm ZonePercentage+  robust glcm Idn+  robust shape SurfaceVolumeRatio | 0.70 | 0.50 | 0.74 |
|  | rdr3y ~ robust ngtdm Coarseness+  robust shape LeastAxisLength+  robust glszm ZonePercentage+  robust shape SurfaceVolumeRatio | 0.68 | 0.54 | 0.75 |
|  | rdr3y ~ robust ngtdm Coarseness+  robust glcm InverseVariance+  robust gldm SmallDependenceEmphasis+  robust shape SurfaceVolumeRatio | 0.63 | 0.68 | 0.77 |
|  | rdr3y ~ robust gldm SmallDependenceEmphasis+  robust ngtdm Coarseness+robust glcm Idn | 0.65 | 0.63 | 0.74 |
|  | rdr3y ~ robust gldm SmallDependenceEmphasis+  robust glcm Idn | 0.58 | 0.65 | 0.71 |

Supplementary Table S6b - Individual predictive performance of the selected model per repetition on the 3-year post-RT regional or distant recurrence endpoint

| **feat. group** | **formula** | **concordance train** | **concordance int. val.** | **concordance ext. val.** |
| --- | --- | --- | --- | --- |
| all | Surv(rdr) ~HD gldm GrayLevelNonUniformity+  HD glcm JointEnergy | 0.61 | 0.62 | 0.58 |
|  | Surv(rdr) ~LD shape Flatness | 0.53 | 0.53 | 0.59 |
|  | Surv(rdr) ~HD gldm LargeDependenceLowGrayLevelEmphasis | 0.56 | 0.51 | 0.73 |
|  | Surv(rdr) ~HD glrlm LowGrayLevelRunEmphasis+  LD shape VoxelVolume | 0.62 | 0.55 | 0.62 |
|  | Surv(rdr) ~LD glcm ClusterProminence | 0.51 | 0.53 | 0.43 |
|  | Surv(rdr) ~HD glrlm RunVariance+  LD shape Flatness | 0.62 | 0.61 | 0.61 |
|  | Surv(rdr) ~HD glrlm LongRunEmphasis+  LD shape Flatness | 0.68 | 0.50 | 0.61 |
|  | Surv(rdr) ~LD shape MeshVolume | 0.63 | 0.57 | 0.60 |
|  | Surv(rdr) ~LD ngtdm Busyness+  LD firstorder Median | 0.66 | 0.57 | 0.58 |
|  | Surv(rdr) ~HD glrlm GrayLevelNonUniformity+  LD gldm LargeDependenceLowGrayLevelEmphasis | 0.62 | 0.56 | 0.64 |
| HD | Surv(rdr) ~HD glcm JointEnergy+  HD gldm GrayLevelNonUniformity | 0.61 | 0.61 | 0.55 |
|  | Surv(rdr) ~HD glcm MaximumProbability | 0.60 | 0.63 | 0.53 |
|  | Surv(rdr) ~HD gldm DependenceVariance+  HD shape MeshVolume | 0.65 | 0.50 | 0.66 |
|  | Surv(rdr) ~HD glcm MaximumProbability | 0.61 | 0.58 | 0.53 |
|  | Surv(rdr) ~HD gldm LargeDependenceEmphasis | 0.62 | 0.56 | 0.63 |
|  | Surv(rdr) ~HD firstorder Uniformity | 0.65 | 0.49 | 0.45 |
|  | Surv(rdr) ~HD glszm LargeAreaEmphasis | 0.61 | 0.58 | 0.67 |
|  | Surv(rdr) ~HD glrlm LongRunEmphasis+  HD glcm JointEnergy+  HD glcm MaximumProbability | 0.62 | 0.58 | 0.63 |
|  | Surv(rdr) ~HD glrlm RunVariance+  HD gldm LowGrayLevelEmphasis | 0.61 | 0.59 | 0.63 |
|  | Surv(rdr) ~HD glcm MaximumProbability | 0.61 | 0.58 | 0.53 |
| LD | Surv(rdr) ~LD gldm DependenceNonUniformity | 0.61 | 0.58 | 0.58 |
|  | Surv(rdr) ~LD gldm DependenceVariance | 0.62 | 0.59 | 0.62 |
|  | Surv(rdr) ~LD glrlm GrayLevelNonUniformity+  LD shape MeshVolume | 0.60 | 0.66 | 0.60 |
|  | Surv(rdr) ~LD shape Flatness | 0.53 | 0.52 | 0.59 |
|  | Surv(rdr) ~LD shape MeshVolume+  LD shape Flatness+  LD firstorder Energy+  LD shape SurfaceArea | 0.64 | 0.55 | 0.60 |
|  | Surv(rdr) ~LD firstorder Energy | 0.58 | 0.58 | 0.56 |
|  | Surv(rdr) ~LD ngtdm Busyness+  LD shape MeshVolume | 0.66 | 0.47 | 0.58 |
|  | Surv(rdr) ~LD ngtdm Busyness+  LD gldm LargeDependenceLowGrayLevelEmphasis | 0.59 | 0.66 | 0.57 |
|  | Surv(rdr) ~LD glcm JointEntropy | 0.63 | 0.55 | 0.58 |
|  | Surv(rdr) ~LD shape MeshVolume | 0.64 | 0.55 | 0.60 |
| robust | Surv(rdr) ~robust shape MinorAxisLength | 0.61 | 0.57 | 0.67 |
|  | Surv(rdr) ~robust glrlm RunPercentage+  robust shape MeshVolume | 0.58 | 0.67 | 0.67 |
|  | Surv(rdr) ~robust glrlm RunPercentage+  robust shape VoxelVolume+  robust glrlm ShortRunEmphasis | 0.62 | 0.58 | 0.66 |
|  | Surv(rdr) ~robust glrlm RunPercentage+  robust shape MeshVolume | 0.63 | 0.53 | 0.67 |
|  | Surv(rdr) ~robust glrlm GrayLevelNonUniformity | 0.60 | 0.59 | 0.63 |
|  | Surv(rdr) ~robust glrlm RunPercentage+  robust glrlm GrayLevelNonUniformity | 0.64 | 0.50 | 0.66 |
|  | Surv(rdr) ~robust glrlm RunPercentage+  robust gldm GrayLevelNonUniformity+  robust glrlm ShortRunEmphasis | 0.64 | 0.51 | 0.64 |
|  | Surv(rdr) ~robust glrlm RunPercentage+  robust shape MeshVolume | 0.62 | 0.55 | 0.67 |
|  | Surv(rdr) ~robust glrlm ShortRunEmphasis+  robust shape SurfaceArea | 0.59 | 0.62 | 0.68 |
|  | Surv(rdr) ~robust gldm GrayLevelNonUniformity+  robust glrlm GrayLevelNonUniformity | 0.59 | 0.59 | 0.64 |

Supplementary Table S6c - Individual predictive performance of the selected model per repetition on regional or distant recurrence free survival endpoint

| **set** | **group1** | **group2** | **n1** | **n2** | **statistic** | **p** | **p.adj** | **p.adj.signif** |
| --- | --- | --- | --- | --- | --- | --- | --- | --- |
| train | all | HD | 10 | 10 | 44 | 0.684 | 0.786 | ns |
|  | all | LD | 10 | 10 | 62 | 0.393 | 0.786 | ns |
|  | all | robust | 10 | 10 | 9 | 0.001 | 0.006 | ** |
|  | HD | LD | 10 | 10 | 68 | 0.19 | 0.57 | ns |
|  | HD | robust | 10 | 10 | 11 | 0.002 | 0.01 | * |
|  | LD | robust | 10 | 10 | 16 | 0.009 | 0.036 | * |
| Int. val. | all | HD | 10 | 10 | 38 | 0.393 | 1 | ns |
|  | all | LD | 10 | 10 | 34 | 0.247 | 1 | ns |
|  | all | robust | 10 | 10 | 41 | 0.529 | 1 | ns |
|  | HD | LD | 10 | 10 | 39 | 0.436 | 1 | ns |
|  | HD | robust | 10 | 10 | 48 | 0.912 | 1 | ns |
|  | LD | robust | 10 | 10 | 59 | 0.529 | 1 | ns |
| Ext. val. | all | HD | 10 | 10 | 34.5 | 0.257 | 0.771 | ns |
|  | all | LD | 10 | 10 | 44 | 0.677 | 1 | ns |
|  | all | robust | 10 | 10 | 4.5 | 0.000667 | 0.004 | ** |
|  | HD | LD | 10 | 10 | 57 | 0.623 | 1 | ns |
|  | HD | robust | 10 | 10 | 19 | 0.018 | 0.074 | ns |
|  | LD | robust | 10 | 10 | 11.5 | 0.004 | 0.02 | * |

Supplementary Table S7a – Pairwise Wilcoxon-Mann-Whitney test with Holm correction between predictive performance distributions on regional or distant recurrence endpoint.

| **set** | **group1** | **group2** | **n1** | **n2** | **statistic** | **p** | **p.adj** | **p.adj.signif** |
| --- | --- | --- | --- | --- | --- | --- | --- | --- |
| train | all | HD | 10 | 10 | 44 | 0.677 | 1 | ns |
|  | all | LD | 10 | 10 | 41 | 0.52 | 1 | ns |
|  | all | robust | 10 | 10 | 13 | 0.006 | 0.026 | * |
|  | HD | LD | 10 | 10 | 39 | 0.436 | 1 | ns |
|  | HD | robust | 10 | 10 | 8 | 0.000725 | 0.004 | ** |
|  | LD | robust | 10 | 10 | 14 | 0.005 | 0.026 | * |
| test | all | HD | 10 | 10 | 47 | 0.853 | 1 | ns |
|  | all | LD | 10 | 10 | 48 | 0.912 | 1 | ns |
|  | all | robust | 10 | 10 | 32 | 0.19 | 1 | ns |
|  | HD | LD | 10 | 10 | 52 | 0.912 | 1 | ns |
|  | HD | robust | 10 | 10 | 33.5 | 0.226 | 1 | ns |
|  | LD | robust | 10 | 10 | 32 | 0.19 | 1 | ns |
| ext | all | HD | 10 | 10 | 40.5 | 0.495 | 0.99 | ns |
|  | all | LD | 10 | 10 | 58.5 | 0.544 | 0.99 | ns |
|  | all | robust | 10 | 10 | 3 | 0.00042 | 0.002 | ** |
|  | HD | LD | 10 | 10 | 74 | 0.075 | 0.226 | ns |
|  | HD | robust | 10 | 10 | 0 | 0.000177 | 0.001 | ** |
|  | LD | robust | 10 | 10 | 0 | 0.000177 | 0.001 | ** |

Supplementary Table S7b – Pairwise Wilcoxon-Mann-Whitney test with Holm correction between predictive performance distributions on 3-year post-RT regional or distant recurrence endpoint.

| **set** | **group1** | **group2** | **n1** | **n2** | **statistic** | **p** | **p.adj** | **p.adj.signif** |
| --- | --- | --- | --- | --- | --- | --- | --- | --- |
| train | all | HD | 10 | 10 | 53 | 0.853 | 1 | ns |
|  | all | LD | 10 | 10 | 49 | 0.971 | 1 | ns |
|  | all | robust | 10 | 10 | 49 | 0.971 | 1 | ns |
|  | HD | LD | 10 | 10 | 55 | 0.739 | 1 | ns |
|  | HD | robust | 10 | 10 | 55 | 0.739 | 1 | ns |
|  | LD | robust | 10 | 10 | 51 | 0.971 | 1 | ns |
| test | all | HD | 10 | 10 | 37 | 0.353 | 1 | ns |
|  | all | LD | 10 | 10 | 42 | 0.579 | 1 | ns |
|  | all | robust | 10 | 10 | 42 | 0.579 | 1 | ns |
|  | HD | LD | 10 | 10 | 57 | 0.631 | 1 | ns |
|  | HD | robust | 10 | 10 | 52 | 0.912 | 1 | ns |
|  | LD | robust | 10 | 10 | 48 | 0.912 | 1 | ns |
| ext | all | HD | 10 | 10 | 53 | 0.85 | 1 | ns |
|  | all | LD | 10 | 10 | 66 | 0.237 | 0.711 | ns |
|  | all | robust | 10 | 10 | 12.5 | 0.005 | 0.02 | * |
|  | HD | LD | 10 | 10 | 50 | 1 | 1 | ns |
|  | HD | robust | 10 | 10 | 11.5 | 0.004 | 0.019 | * |
|  | LD | robust | 10 | 10 | 0 | 0.000173 | 0.001 | ** |

Supplementary Table S7c – Pairwise Wilcoxon-Mann-Whitney test with Holm correction between predictive performance distributions on regional or distant recurrence free survival endpoint.
